# Stereoselective Synthesis of Bisfuranoxide (Aurochrome, Auroxanthin) and Monofuranoxide (Equinenone 5′,8′-Epoxide) Carotenoids by Double Horner–Wadsworth–Emmons Reaction

**DOI:** 10.1021/acs.jnatprod.2c00475

**Published:** 2022-09-19

**Authors:** Aurea Rivas, Marta Castiñeira, Rosana Álvarez, Belén Vaz, Angel R. de Lera

**Affiliations:** CINBIO, Universidade de Vigo, Department of Organic Chemistry, Galicia Sur Health Research Institute (IIS Galicia Sur),, 36310 Vigo, Spain

## Abstract

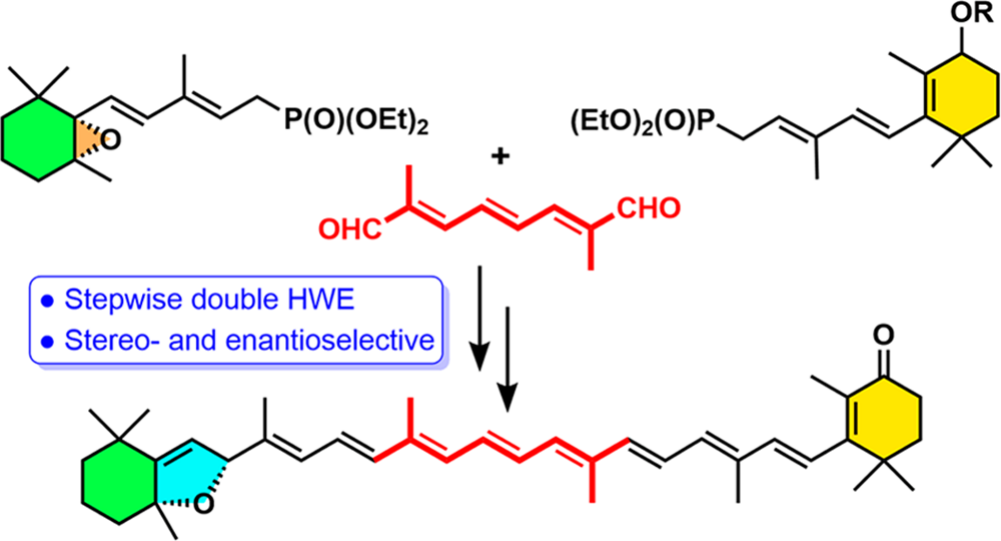

The stereoselective synthesis of C_40_-all-*trans*-carotenoids with the formal hexahydrobenzofuran skeletons aurochrome, auroxanthin, and equinenone-5′,8′-epoxide is reported. The synthesis is based on a one-pot or stepwise double Horner–Wadsworth–Emmons (HWE) reaction of a terminal enantiopure C_15_-5,6-epoxycyclohexadienylphosphonate and a central C_10_-trienedial. The ring expansion of the epoxycyclohexadienylphosphonate, generated by a Stille cross-coupling reaction, to the hexahydrobenzofuran skeleton was promoted by the reaction conditions of the HWE reaction prior to double-bond formation.

Carotenoids^1,2^ are a group of numerous naturally occurring polyenic pigments ubiquitously present in the plant kingdom and other photosynthetic organisms, for which more than 750 compounds have been reported.^3,4^ Being components of the photosynthetic^5^ and photoprotective structural arrangements in these species,^6^ carotenoids play fundamental roles in maintaining life.^7^ Carotenoids are also responsible for the color and stability of some fruits, vegetables, flowers, and birds.^8^ These polyenes hold potential as chemopreventive agents in humans by acting as antioxidants due to the radical-stabilizing ability of their conjugated unsaturated chains.^6,9^ In addition, a plethora of bioactivities have been reported for carotenoids,^10^ including anti-inflammatory, anticancer,^11^ antimetabolic disorders,^12^ and inhibition of lipid peroxidation.^13^ Since the great majority of natural carotenoids are geometrically homogeneous all-*trans* polyene isomers, rather than differing in the double-bond geometries, they show large structural variability at the cyclohexenyl ring and also at the proximal double bonds.^14^

Recent work in this field is focused on exploring the production of large quantities of these polyenic natural products by engineering a variety of carotenoid biosynthetic genes.^15^ For example, the marine-bacterial carotenoid 4,4′-ketolase (4,4′-oxygenase) gene *crtW* has been shown to promote the biogenesis of some 4-ketocarotenoids. Expression of the ketolase *crtW* gene in tubers of sweet potatoes [*Iponomea batatas* (L.) Lam] under the control of the CaMV promoter allowed the generation of novel carotenoids with furanoxide and cyclohexenone functional fragments, for example, echinenone 5′,8′-epoxide (**2a** and **2b**, Scheme 1).^16^ A 60:40 mixture of diastereomers of **2** was obtained when the sweet potato was extracted under normal conditions,^16^ which suggested that these compounds might have been formed through the rearrangement of the putative precursor echinenone 5′,6′-epoxide (**1**, Scheme 1).^16^

**Scheme 1:**
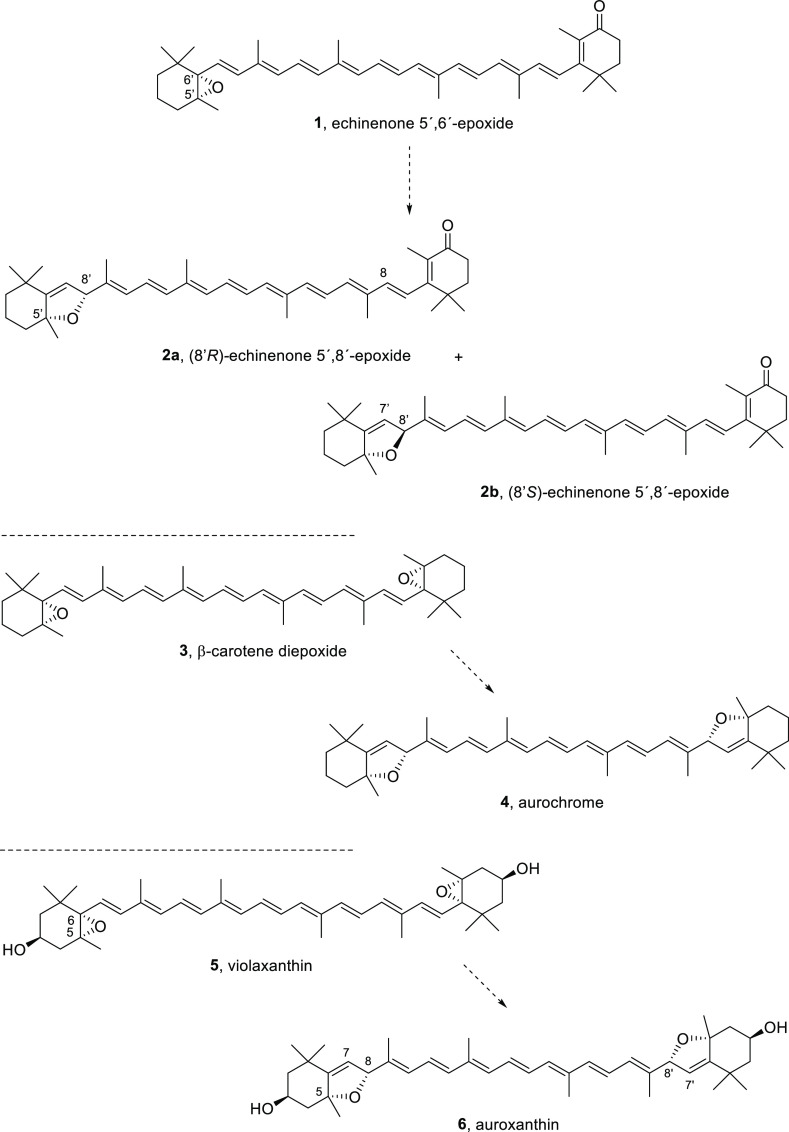
Putative Biogenetic Relationships between Carotenoid 5′,8′-Epoxides/Furanoxides (**2a**, **2b**, **4**, and **6**) and Carotenoid 5′,6′-Epoxides (**1**, **3**, and **5**)

Echinenone-5′,8′-epoxides (**2**) belong to the small group of carotenoids termed epoxycarotenoids, which also include symmetrical members such as aurochrome and auroxanthin (**4** and **6**, respectively, Scheme 1), for which some promising biological activities have been reported.^17^

The bis-furanoxide aurochrome (**4**, Scheme 1) has previously been isolated in very small amounts from green leaves of several Kenyan clones,^18,19^ although its natural occurrence and biogenetic connection to β-carotene diepoxide (**3**, Scheme 1) have not been proven. Auroxanthin (**6**) has been isolated, together with its putative biogenetic precursor, the 5,6-epoxycarotenoid violaxanthin (**5**, Scheme 1),^3,4^ from petals of the yellow *Rosa fetidu* HERRM,^20^ eggs of hens seaweed meal,^21^ and microalga *Chlorella pyrenoidosa* mutant G44.^22,23^ From the former, auroxanthin (**6**) was isolated as a mixture of four diastereomers that differed by the configuration of the dihydrofuran ring (namely, 8*R*,8′*S*, 8*S*,8′*S* and 8*R*,8′*R*) and the geometry of one of the proximal double bonds (9′*Z*,8*R*,8′*R*).^20,24^

The general assumption that the entire subfamily of carotenoids with furanoxide rings fused to the trimethylcyclohexane originates from the rearrangement of structurally related analogues containing 5′,6′-epoxide subunits upon exposure of these functionalities (Scheme 1) to acid media during the isolation and purification protocols^25^ led to the consideration of these natural products as artifacts.^26−28^ However, after careful control experiments, upon subjecting one of the putative butene monoepoxide precursors, i.e., peridinin (not shown), to these conditions, the furanoxide derivatives were not present in the reaction mixture.^29^ Therefore, although peridinin also contains a γ-butenolide substructure, it is currently considered that the furanoxides might indeed be true natural products and not artifacts.

Since the synthesis of nonsymmetrical echinenone 5′,8′-epoxide (**2**, Scheme 1)^16^ has not been reported, we addressed its preparation as a followup^30,31^ of our work on the bidirectional approach to carotenoids (a C_15_ + C_10_ + C_15_ = C_40_ synthetic condensation scheme)^1,32^ using the Horner–Wadsworth–Emmons (HWE) reaction.^33−38^ The condensation of anions of two C_15_ phosphonates (namely, **8** and **11**, Scheme 2) with the common C_10_ linchpin reagent 2,7-dimethyl-2,4,6-triene-1,8-dial (**7**), which has been shown to proceed with high *E*-selectivity in the newly formed olefins,^30,31^ was then considered. In order to establish the protocol, the diastereoselective synthesis of enantiopure aurochrome (**4**) and auroxanthin (**6**) was envisioned, using complementary phosphonates **8**–**10** (Scheme 2) and the same C_10_ central linchpin dialdehyde (**7**).^30−32,39−43,44^ This strategy was previously explored by Acemoglu and Eugster for the racemic material C_15_-phosphonate **8**,^42^ which was reported to provide a complex mixture of four racemic diastereomers and the two *meso*-aurochrome isomers.^45^

**Scheme 2:**
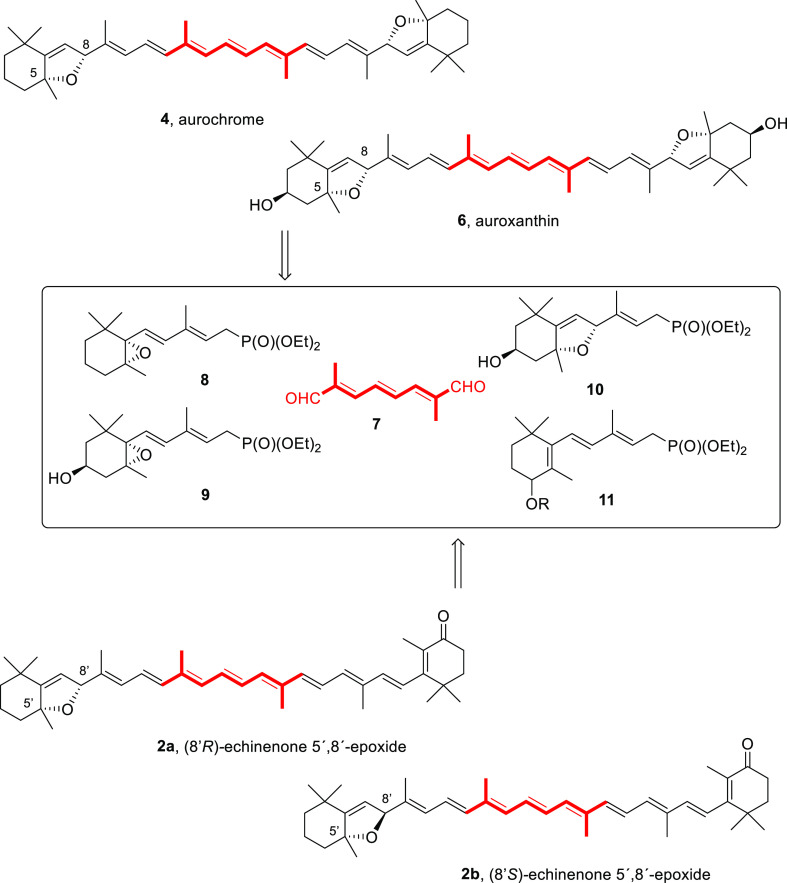
Bis-HWE Condensation of C_10_-Dialdehyde **7** and the Corresponding Phosphonates (**8**–**11**) for the Synthesis of Enantiopure Aurochrome (**4**), Auroxanthin (**6**), and Echinenone 5′,8′-Epoxide (**2a** and **2b**)

## Results and Discussion

**Scheme 3:**
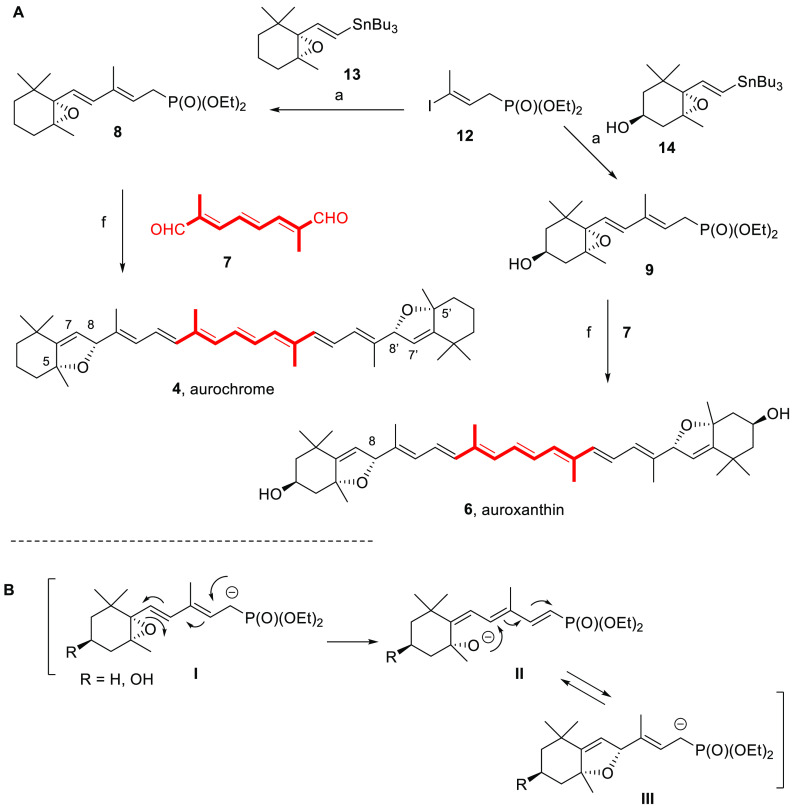
(A) Double HWE Reaction for the Total Synthesis of Enantiopure (5*R*,8*R*,5′*R*,8′*R*)-Aurochrome (**4**) and (3*S*,5*R*,8*R*,3′*S*,5′*R*,8′*R*)-Auroxanthin (**6**); (B) Proposed Reaction Pathway from the Dienylphosphonate Conditions: (a) Pd(PPh_3_)_4_, CuTC, [Ph_2_PO_2_][NBu_4_], DMF, 25 °C, 98% for **8**; 52% for **9**. (b) *i.* KO*t*Bu (2.2 molar equiv for **8**; 4.6 molar equiv for **9**), THF, −30 °C, 30 min; *ii*. −30 to 0 °C, 1 h, 53% for **4**; 65% for **6**.

Following the bidirectional approach to carotenoid synthesis, the doubly functionalized iodo-butenylphosphonate **12** was needed to accomplish the orthogonal Stille cross-coupling and HWE reaction steps of the planned synthesis. This fragment was prepared in 60% overall yield as previously described (Scheme 3).^31^

On route to aurochrome (**4**), the palladium-catalyzed Stille cross-coupling reaction promoted by the cocatalytic effect of Cu(I)^46^ of alkenyl iodide **12** and alkenylstannane **13**(47) afforded epoxypentadienylphosphonate **8** in almost quantitative yield. Reaction conditions for carotenoid formation were first explored by following the procedure described for the racemic material.^28,45^ Upon treatment of **8** with KO*t*Bu (tetrahydrofuran (THF), −30 °C) and reaction of the anion with **7**, the predominant formation of the thermodynamically favored^48^ all-*trans* isomer of the polyene skeleton of aurochrome (**4**) was generated as a 3:1 mixture of diastereomers (Scheme 3A).^45^ A complex reaction mechanism has already been proposed under the reaction conditions to provide aurochrome (**4**, Scheme 3A) following formation of the phosphonate anion **I** stabilized through conjugation, namely, (i) ring-opening of the 5,6-epoxide; (ii) ring-closure by conjugate addition of the generated alkoxide to the trienylphosphonate intermediate **II** (Scheme 3B) to afford the reacting alkenyl-5,8-epoxide phosphonate anion **III**;^45,49^ and (iii) the 2-fold condensation with C_10_-trienedial **7**.^28,45^ Under basic conditions, the **I** to **III** rearrangement was expected to lead predominantly to the formation of the most stable furanoxide phosphonate anion isomer (**III**) and, therefore, to the all-*trans* isomer of the major diastereomer (53% yield), namely, (5*R*,8*R*,5′*R*,8′*R*)-aurochrome (**4**) (Scheme 3).^49^^1^H NMR data confirmed this assumption, since it has been shown that Δδ_H7–H8_ for this diastereomer is very small (0.02 ppm) and the signal for H7 appears as a broad singlet.^27,28^ The 8*R*/8′*R* configuration of the newly formed C8 and C8′ stereocenters for the major diastereomer was further confirmed by the NOE effect observed between the methyl groups at C5/C5′ and proton signals at C8/C8′.

A similar approach was followed for the synthesis of the carotenoid 5,8-furanoxide auroxanthin (**6**). Combination of alkenyl iodide **12** and the previously described alkenylstannane **14**(49,50) also using the Stille reaction performed under Fürstner’s conditions^46^ provided the epoxydienyl phosphonate **9** (Scheme 3). With fragments (C_15_ and C_10_) in hand, the stereoselective synthesis of auroxanthin (**6**) followed the same procedure described for aurochrome (**4**). Thus, the reaction of **7** with the anion generated upon treatment of phosphonate-5,6-epoxide **9** with an excess of base (4.6 equiv of KO*t*Bu) in THF afforded, after allowing the mixture to react from −30 to 0 °C, auroxanthin (**6**) in 65% yield in a 3:1 diastereoisomeric ratio (Scheme 3). As indicated for the synthesis of aurochrome (**4**, Scheme 3B), the conjugate addition of the alkoxide anion to C8 of the trienyl phosphonate intermediate should afford the corresponding 5,8-furanoxide allyl phosphonate, itself involved as intermediate in the HWE condensation with triene dialdehyde **7** to provide auroxanthin (**6**).

Alternative protocols were explored for the synthesis of enantiopure auroxanthin (**6**), by changing the reaction components and by using phosphonate **10** (Scheme 2) and also acid-promoted rearrangement of the epoxydiene fragment **9**. However, the outcome of these alternative procedures was disappointing (see the SI for a detailed study).

Auroxanthin (**6**) was purified by column chromatography on nitrile-functionalized silica gel, and its spectroscopic data fully matched those described in the literature.^20^ Similarly to the characterization of aurochrome (**4**), the ^1^H NMR data of the major diastereomer of synthetic auroxanthin (**6**) showed characteristic chemical shift values for H7 (δ ≈ 5.25 ppm) and H8 (δ ≈ 5.16 ppm). The data allowed confirming the relative configuration of the dihydrofuran fragments for this diastereomer and the *R* configuration at the newly created stereocenters at C8 and C8′ (Scheme 3).^28^

**Scheme 4:**
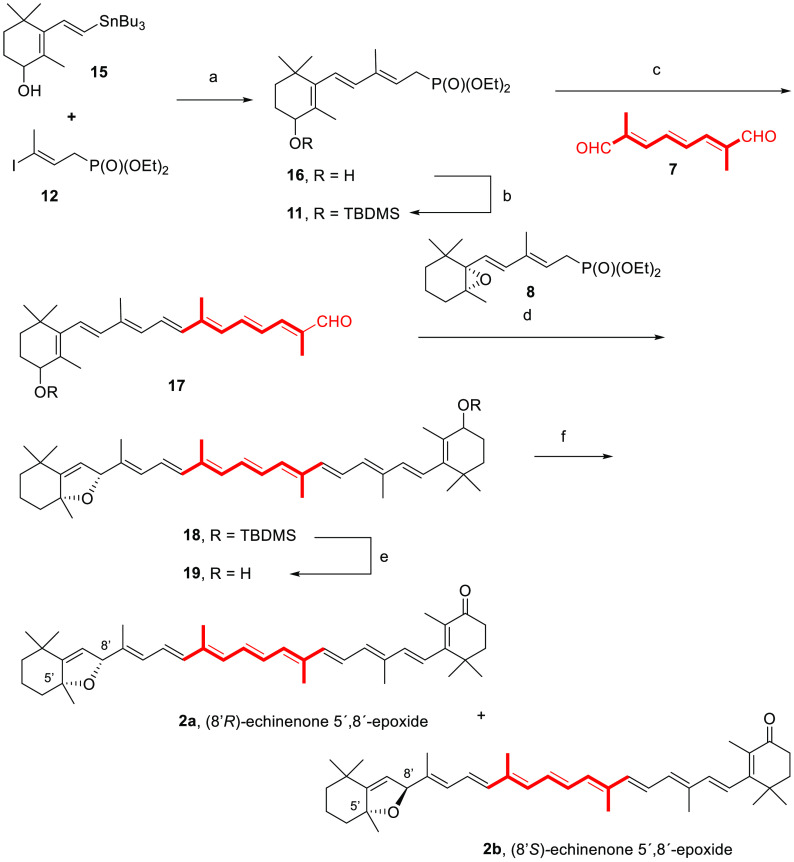
Double HWE Reaction for the Synthesis of Echinenone 5′,8′-Epoxide (**2a/2b**) Reagents and reaction conditions: (a) Pd(PPh_3_)_4_, CuTC, [Ph_2_PO_2_][NBu_4_], DMF, 25 °C, 99%; (b) TBDMSCl, imidazole, DMF, 0 to 25 °C, 82%; (c) NaHMDS, THF, −78 to −30 °C, 1 h, 89%; (d) *i.* KO*t*Bu THF, −30 °C, 30 min; *ii*. −30 to 0 °C, 1 h; (e) TBAF, THF, 25 °C, 47% (combined yield); (f) IBX, DMSO, 25 °C, 30 h, 74%.

Based on the previous results, a similar approach was next applied to the synthesis of enantiopure echinenone 5′,8′-epoxide (**2a**, Scheme 4). The choice of reacting components and construction ordering was entertained by first assaying the mono-HWE condensation of **8** and **7**. The selective HWE condensation (KO*t*Bu, THF, −30 °C) was not feasible in this case, and dimeric aurochrome (**4**) was mainly generated, accompanied by small amounts of the desired interrupted HWE (monocondensation) product. Alternatively, heptatrienyl phosphonate **16** was obtained by Stille cross-coupling reaction of dienylstannane **15**(51) and alkenyliodide **12** under the conditions described above. The allylic hydroxyl group of **16** was protected as silyl ether (TBDMSCl, imidazole, dimethylformamide (DMF), 82%), and the resulting trienylphosphonate **11** was deprotonated by using sodium bis(trimethylsilyl)amide (NaHMDS) in THF (from −78 to −30 °C). Mono-HWE condensation^52^ of the phosphonate anion with **7** afforded the conjugated heptaenal **17** in 89% yield. Further treatment with epoxypentadienylphosphonate **8** under the conditions previously optimized (KO*t*Bu, THF, −30 °C) for auroxanthin (**6**) gave rise to the carotenoid skeleton. Subsequent alcohol protection provided the mixture of protected diastereomeric 5′,8′-epoxides **18** in 47% yield (combined yield for both steps). The alternative condensation of the conjugated anion of phosphonate **11** (KO*t*Bu, THF, −30 °C) with triene dialdehyde **7** under Barbier conditions (addition of **7**, from −30 to 0 °C, 1 h) provided a mixture of conjugated products (**18**) in similar yield but with a lower selectivity at the newly formed C8′ stereocenter. Without separation, the mixture of diastereomeric 5′,8′-epoxides **18** was deprotected (tetra-*n*-butylammonium fluoride (TBAF), THF) to afford the diastereomeric allylic alcohols **19**. The latter were oxidized with freshly prepared 2-iodoxybenzoic acid (IBX) in DMSO^53^ at 25 °C to afford a 4:1 mixture of epimeric echinenone-5′,8′-epoxides (**2a/2b**) in a 74% combined yield (Scheme 4). These epimers were separated by HPLC and spectroscopically characterized. In order to compare the data with those reported,^16^ the ^1^H NMR spectra were recorded in CDCl_3_, showing full consistency with those of the 8*R* and 8*S* epimers. However, due to the lability of these compounds, full NMR characterization was performed in C_6_D_6_. As described above for aurochrome (**4**) and auroxanthin (**6**), whereas the identification of the 8*R* diastereomer of echinenone 5′,8′-epoxide (**2a**) relied on the similar ^1^H NMR chemical shift values reported for H7′ (δ ≈ 5.17 ppm) and H8′ (δ ≈ 5.16 ppm),^16^ the 8*S* diastereomer (**2b**) showed different ^1^H NMR chemical shift values for these hydrogen atoms (H7′, δ ≈ 5.23 ppm; H8′, δ ≈ 5.07 ppm).^28^ Analysis of NOE data proved that the major isomer synthetically prepared featured the relative configuration of the furanoxide ring as expected, thus matching the results just described for aurochrome (**4**) and auroxanthin (**6**).

## Conclusions

In summary, the HWE condensation reaction using a synthetic scheme based on a C_15_ + C_10_ + C_15_ = C_40_ pattern has been demonstrated to be a powerful tool for the stereocontrolled synthesis of the major 5*R*,8*R* diastereomers of enantiopure aurochrome (**4**), auroxanthin (**6**), and recently reported echinenone 5′,8′-epoxide (**2a**). This strategy makes use of a central C_10_-dialdehyde **7** and terminal enantiopure C_15_-dienylphosphonates having a C5,C6-epoxide as a common functionality. An HWE reaction and stereoretentive C5,C6 epoxide ring expansion to the C5,C8 dihydrofuran catalyzed by the basic media concomitantly took place and provided the 5,8-dihydrofuranoxide skeletons of aurochrome (**4**) and auroxanthin (**6**) in a 3:1 diastereomeric ratio. Moreover, for echinenone 5′,8′-epoxide (**2a/b**) the process was performed in a stepwise manner, with the second HWE reaction conditions promoting both C5,C6 epoxide ring expansion to C5,C8 dihydrofuran and double-bond formation and affording the nonsymmetrical carotenoid in a 4:1 diastereomeric ratio. The low efficiency of the putative biogenesis of echinenone 5′,8′-epoxide (**2a/2b**) from *crtW* gene-expressed sweet potato tubers (0.1 mg was isolated from 100 g), which prevented the separation of these diastereomers,^16^ further supports the relevance of the chemical synthesis to access the required amounts of these substrates for further analysis and biological evaluation.

## Experimental Section

### General Experimental Procedures

Solvents were dried according to published methods and distilled before use except THF, CH_2_Cl_2_, CH_3_CN, MeOH, Et_2_O, and DMF, which were dried using a Puresolv solvent purification system. All other reagents were commercial compounds of the highest purity available. All reactions were carried out under an argon atmosphere, and those not involving aqueous reagents were carried out in oven-dried glassware. All solvents and anhydrous solutions were transferred through syringes and cannulae, previously dried in the oven for at least 12 h and kept in a desiccator with KOH. Et_3_N, acetone, diisopropylamine, *N*,*N*-diisopropylethylamine (DIPEA), and pyridine were dried by distillation from CaH_2_. Distillations were carried out in a Büchi GKR-50 Kügelrohr, and in that case the boiling points indicate the external temperature. For fractional distillations a microstill was used with an internal thermometer in the distillation head. The *n*BuLi concentration was determined by titration in triplicate with diphenyl acetic acid or *N*-pivaloyl-*o*-toluidine in THF at 0 °C. For reactions at low temperature, ice/water or CO_2_/acetone systems were used. For different temperatures, a HaaKe EK90 immersion cooler (−90 to −15 °C) was used. Analytical thin-layer chromatography (TLC) was performed on aluminum plates with silica gel Merk Kiesegel 60F_254_ or in glass plates with silica gel 60 RP-18 F_254_s or silica gel 60 CN F_254_s and visualized by UV irradiation (254 or 365 nm) or by staining with a solution of phosphomolybdic acid, KMnO_4_, DNP (2,4-dinitrophenylhydrazine), or anisaldehyde. Flash column chromatography was carried out using Merck Kieselgel 60 (230–400 mesh) or Silicycle SilicaFlash P60 (230–400 mesh), Merck Preparative C_18_ (125 Å, 55–105 μm), or Redisep Rf CN (100 Å, 400–632 mesh) under pressure. Alternatively, an AnaLogix Intelliflash 310 HPFC flash collector system was used. IR spectra were obtained with a JASCO FTIR 4200 spectrophotometer, from a thin film deposited onto NaCl glass. Specific rotations were measured on a JASCO P-1020 polarimeter with a Na lamp. HPLC was performed using a Waters instrument by using a dual-wavelength detector and a 3.5 × 100 mm glass cell. UV were developed in a Cary C BIO spectrometer in methanol as solvent. HRMS (ESI^+^) were measured with an Apex III FTICR mass spectrometer (Bruker Daltonics). ^1^H NMR spectra and ^13^C NMR spectra were recorded in CDCl_3_, C_6_D_6_, CD_3_OD, and (CD_3_)_2_CO at ambient temperature on a Bruker AMX-400 spectrometer operating at 400.16 and 100.62 MHz with residual protic solvent as the internal reference (CDCl_3_, δ = 7.26 ppm; C_6_D_6_, δ = 7.16 ppm; (CD_3_)_2_CO, δ = 2.05 ppm; and CD_3_OD, δ = 4.87 ppm) for the former and CDCl_3_ (δ_C_ = 77.2 ppm), C_6_D_6_ (δ_C_ = 128.0 ppm), (CD_3_)_2_CO (δ_C_ = 29.8 ppm), and CD_3_OD (δ_C_ = 49.0 ppm) as the internal reference for the latter. Chemical shifts (δ) are given in parts per million (ppm), and coupling constants (*J*) are given in hertz (Hz). The proton spectra are reported as follows: δ (multiplicity, coupling constant *J*, number of protons). A DEPT-135 pulse sequence was used to aid in the assignment of signals in the ^13^C NMR spectra. Multiplicity in the ^13^C NMR spectral data refers to the attached hydrogens.

### Synthesis of (5*R*,8*R*,5*R′*,8*R′*)-Aurochrome (**4**)

#### Diethyl (2*E*,4*E*)-3-Methyl-5-((1*S*,6*R*)-2,2,6-trimethyl-7-oxabicyclo[4.1.0]heptan-1-yl)penta-2,4-dien-1-yl]phosphonate (**8**)

A degassed solution of diethyl (*E*)-(3-iodobut-2-en-1-yl)phosphonate (**12**) (90.0 mg, 0.28 mmol) and tributyl ((*E*)-2-((1*S*,6*R*)-2,6,6-trimethyl-7-oxabicyclo[4.1.0]heptan-1-yl)vinyl)stannane (**13**) (148.1 mg, 0.32 mmol) in DMF (4.7 mL) was added to a flask containing flamed-dried [NBu_4_][Ph_2_PO_2_] (156.1 mg, 0.34 mmol) at 25 °C. Then, CuTC (80.9 mg, 0.42 mmol) was added, followed by Pd(PPh_3_)_4_ (16.3 mg, 0.014 mmol), and the resulting mixture was stirred for 2 h at 25 °C. Water was then added, the layers were separated, and the aqueous layer was extracted with EtOAc (3×). The combined organic layers were dried (Na_2_SO_4_), and the solvent was evaporated. The residue was purified by flash-column chromatography (silica gel, gradient from 60:40 v/v hexane/EtOAc to EtOAc) to afford 82.5 mg (98%) of a pale yellow oil identified as diethyl (2*E*,4*E*)-3-methyl-5-((1*S*,6*R*)-2,2,6-trimethyl-7-oxabicyclo[4.1.0]heptan-1-yl)-penta-2,4-dien-1-yl]phosphonate (**8**): [α]^21^_D_ −17 (*c* 1.02, CHCl_3_). ^1^H NMR (400.13 MHz, CD_3_OD): δ 6.21 (d, *J* = 15.7 Hz, 1H, H_4_), 5.92 (dd, *J* = 15.7, 2.5 Hz, 1H, H_5_), 5.45 (q, *J* = 7.7 Hz, 1H, H_2_), 4.16–4.04 (m, 4H, 2×OCH_2_CH_3_), 2.81 (dd, ^2^*J*_H*–*P_ = 23.0 Hz, *J*_H–H_ = 8.1 Hz, 2H, 2H_1_), 1.89–1.77 (m, 2H, 2H_5′_), 1.83 (d, ^5^*J*_H–P_ = 4.2 Hz, 3H, CH_3_), 1.49–1.38 (m, 2H, 2H_4′_), 1.31 (t, *J* = 7.0 Hz, 6H, 2×OCH_2_CH_3_), 1.15–1.07 (m, 2H, 2H_3′_), 1.13 (s, 3H, C6′-CH_3_), 1.11 (s, 3H, C2′-CH_3_), 0.91 (s, 3H, C2′-CH_3_) ppm. ^13^C NMR (100.62 MHz, CD_3_OD): δ 138.7 (s, *J*_C–P_ = 14.6 Hz), 137.7 (d, *J*_C–P_ = 5.4 Hz), 125.1 (d, *J*_C–P_ = 4.4 Hz), 120.5 (d, ^2^*J*_C–P_ = 12.7 Hz), 72.8 (s, *J*_C–P_ = 1.6 Hz), 67.0 (s, *J*_C–P_ = 1.6 Hz), 63.6 (t, 2×, *J*_C–P_ = 6.9 Hz), 36.8 (t, Hz), 34.7 (s), 31.0 (t), 27.0 (t, ^1^*J*_C–P_ = 139.6 Hz), 26.3 (q), 26.2 (q), 21.3 (q), 18.1 (t), 16.7 (q, *J*_C–P_ = 5.9 Hz, 2×), 12.9 (q, *J*_C–P_ = 2.6 Hz) ppm. IR (NaCl): ν 2955 (m, C–H), 1220 (s, P=O), 1032 (s, P–O) cm^–1^. HRMS (ESI^+^): calcd for C_19_H_34_O_4_P ([M + H]^+^), 357.2181; found, 357.2189.

#### (5*R*,8*R*,5*R′*,8*R′*)-Aurochrome (**4**)

To a cooled (−30 °C) solution of diethyl (2*E*,4*E*)-3-methyl-5-((1*S*,6*R*)-2,2,6-trimethyl-7-oxabicyclo[4.1.0]heptan-1-yl)-penta-2,4-dien-1-yl]phosphonate(**8**) (25.4 mg, 0.085 mmol) in THF (0.1 mL) was added KO*t*Bu (0.081 mL, 1 M in hexane, 0.081 mmol). After stirring for 30 min, a solution of (2*E*,4*E*,6*E*)-2,7-dimethylocta-2,4,6-triene-1,8-dial (**7**) (6 mg, 0.037 mmol) in THF (0.1 mL) was added. The mixture was stirred from −30 to 0 °C for 1 h. Then, a saturated aqueous solution of NH_4_Cl was added, and the mixture was extracted with a 90:10 v/v EtOAc/CH_2_Cl_2_ mixture. The combined organic layers were washed with a saturated aqueous solution of NaHCO_3_ and dried (Na_2_SO_4_), and the solvent was evaporated. Purification by flash-column chromatography (CN-silica gel, from 90:10 to 60:40 v/v hexane/EtOAc) afforded 13 mg (53%) of an orange solid identified as (5*R*,8*R*,5′*R*,8′*R*)-aurochrome (**4**) and 4 mg (17%) of (5*R*,8*S*,5′*R*,8′*S*)-aurochrome (**4**).

#### Data for (5*R*,8*R*,5′*R*,8′*R*)-aurochrome (**4**)

^1^H NMR (400.13 MHz, CDCl_3_): δ 6.60 (app dd, *J* = 7.8, 2.9 Hz, 2H, H_15_ + H_15′_), 6.49 (dd, *J* = 15.0, 11.0 Hz, 2H, H_11_ + H_11′_), 6.31 (d, *J* = 15.0 Hz, H_12_ + H_12′_), 6.24 (d, *J* = 12.1 Hz, H_14_ + H_14′_), 6.18 (d, *J* = 11.0 Hz, H_10_ + H_10′_), 5.17 (s, 2H, H_8_ + H_8′_), 5.15 (s, 2H, H_7_ + H_7′_), 1.94 (s, 6H, 2×CH_3_), 1.98–1.92 (m, 2H, H_4a_ + H_4a′_), 1.73 (s, 6H, 2×CH_3_), 1.68–1.60 (m, 2H, H_4b_ + H_4b′_), 1.56–1.48 (m, 6H, H_3_ + H_3′_ + H_2_ + H_2′_), 1.42 (s, 6H, 2×CH_3_), 1.25–1.20 (m, 2H, H_2_ + H_2′_), 1.15 (s, 6H, 2×CH_3_), 1.10 (s, 6H, 2×CH_3_) ppm. ^1^H NMR (400.13 MHz, C_6_D_6_): δ 6.70 (dd, *J* = 15.0, 11.0 Hz, H_11_ + H_11′_), 6.68–6.66 (m, 2H, H_15_ + H_15′_), 6.48 (d, *J* = 14.9 Hz, 2H, H_12_ + H_12′_), 6.45 (d, *J* = 11.1 Hz, 2H, H_10_ + H_10′_) 6.28 (d, *J* = 9.4 Hz, H_14_ + H_14′_), 5.33 (s, 2H, H_8_ + H_8′_), 5.11 (s, 2H, H_7_ + H_7′_), 2.00 (d, *J* = 12.2 Hz, 2H, H_4a_ + H_4a′_), 1.86 (s, 6H, 2×CH_3_), 1.85 (s, 6H, 2×CH_3_), 1.74–1.64 (m, 2H, H_4b_ + H_4b′_), 1.49 (s, 6H, 2×CH_3_), 1.48–1.38 (m, 4H, H_2_ + H_2′_), 1.34–1.25 (m, 2H, H_3a_ + H_3a′_), 1.15–1.07 (m, 2H, H_3b_ + H_3b′_), 1.05 (s, 6H, 2×CH_3_), 0.98 (s, 6H, 2×CH_3_) ppm. ^13^C NMR (101 MHz, C_6_D_6_): δ 154.3 (s, C6 + C6′), 138.8 (s, C9 + C9′), 137.5 (d, C12 + C12′), 136.0 (s, C13 + C13′), 132.5 (d, C14 + C14′), 130.1 (d, C15 + C15′), 126.9 (d, C10 + C10′), 124.7 (d, C11 + C11′), 119.4 (d, C7 + C7′), 87.7 (d, C8 + C8′), 87.2 (s, C5 + C5′), 41.4 (t, C4 + C4′), 41.3 (t, C2 + C2′), 34.2 (s, C1 + C1′), 30.5 (q, C16 + C16′), 26.0 (q, C18 + C18′), 25.7 (q, C17 + C17′), 20.4 (t, C3 + C3′), 12.7 (q, C20 + C20′), 12.6 (q, C19 + C19′) ppm. HRMS (ESI^+^): calcd for C_40_H_57_O_2_ ([M + H]^+^), 569.4341; found, 569.4353. UV (MeOH): λ_max_ 380, 401, 426 nm.

#### Data for (5*R*,8*R*,5′*R*,8′*S*)-aurochrome (**4**)

The data matched those described previously.^27,28^

### Synthesis of (3*S*,5*R*,8*R*,3′*S*,5′*R*,8′*R*)-Auroxanthin (**6**)

#### Diethyl (2*E*,4*E*,1′*S*,4′*S*,6′*R*)-[5-(4-Hydroxy-2,2,6-trimethyl-7-oxabicyclo[4.1.0]heptan-1-yl)-3-methylpenta-2,4-dien-1-yl]phosphonate (**9**)

Following the described procedure for the Stille coupling, the reaction of diethyl (*E*)-(3-iodobut-2-en-1-yl)phosphonate (**12**) (147.9 mg, 0.5 mmol), (1*R*,3*S*,6*S*,1′*E*)-6-[2-tributylstannylethen-1-yl]-1,5,5-trimethyl-7-oxabicyclo[4.1.0]heptan-3-ol (**14**) (233.3 mg, 0.58 mmol), [NBu_4_][Ph_2_PO_2_] (273.1 mg, 0.59 mmol), CuTC (141.6 mg, 0.74 mmol), and Pd(PPh_3_)_4_ (28.6 mg, 0.03 mmol) in DMF (6.4 mL) at 25 °C for 1 h afforded, after purification by flash-column chromatography (C-18 silica gel, from 50:50 to 75:25 v/v CH_3_CN/H_2_O), 92.3 mg (52%) of a pale yellow solid identified as diethyl (2*E*,4*E*,1′*S*, 4′*S*,6′*R*)-[5-(4-hydroxy-2,2,6-trimethyl-7-oxabicyclo[4.1.0]heptan-1-yl)-3-methylpenta-2,4-dien-1-yl]phosphonate (**9**): [α]^24^_D_ −50 (*c* 1.19, MeOH). ^1^H NMR (400.13 MHz, CD_3_OD): δ 6.20 (d, *J* = 15.6 Hz, 1H, H_4_), 5.93 (d, *J* = 15.6 Hz, 1H, H_5_), 5.44 (app q, *J* = 8.0 Hz, ^3^*J*_H*–*P_ = 8.0 Hz, 1H, H_2_), 4.13–4.03 (m, 4H, 2×OCH_2_CH_3_), 3.79–3.69 (m, 1H, H_4′_), 2.78 (d, *J* = 8.2 Hz, ^2^*J*_H*–*P_ = 23.0 Hz, 2H, 2H_1_), 2.27 (dd, *J* = 13.9, 5.3 Hz, 1H, H_5′A_), 1.81 (d, ^5^*J*_H*–*P_ = 4.1 Hz, 3H, C_3_–CH_3_), 1.70–1.51 (m, 2H, H_3′A_ + H_5′B_), 1.41–1.32 (m, 1H, H_3′B_), 1.29 (t, *J* = 7.0 Hz, 6H, 2×OCH_2_CH_3_), 1.14 (s, 3H, CH_3_), 1.11 (s, 3H, CH_3_), 0.93 (s, 3H, CH_3_) ppm. HRMS (ESI^+^): calcd for C_19_H_34_O_5_P ([M + H]^+^), 373.2138; found, 373.2136. ^13^C NMR (100.62 MHz, CD_3_OD): δ 138.6 (s, ^3^*J*_C–P_ = 14.8 Hz), 137.6 (d, *J*_C–P_ = 5.5 Hz), 125.5 (d, *J*_C–P_ = 4.5 Hz), 120.7 (d, ^2^*J*_C–P_ = 12.8 Hz), 71.5 (s, *J*_C–P_ = 1.5 Hz), 68.3 (s, *J*_C–P_ = 1.5 Hz), 64.5 (d), 63.6 (t, ^2^*J*_C–P_ = 7.0 Hz), 48.0 (t), 41.6 (t), 36.1 (s), 30.1 (q), 27.0 (t, ^1^*J*_C–P_ = 139.8 Hz), 25.1 (q), 20.2 (q), 16.7 (q, ^5^*J*_C–P_ = 6.3 Hz), 12.9 (q, ^4^*J*_C–P_ = 2.9 Hz) ppm. IR (NaCl): ν 3600–3100 (br, O–H), 2960 (s, C–H), 2927 (s, C–H), 1245 (m, P=O), 1051 (s, P–O–C) cm^–1^.

#### (3*S*,5*R*,8*R*,3′*S*,5′*R*,8′*R*)-Auroxanthin (**6**)

To a cooled (−30 °C) solution of diethyl (2*E*,4*E*,1′*S*,4′*S*,6′*R*)-[5-(4-hydroxy-2,2,6-trimethyl-7-oxabicyclo[4.1.0]heptan-1-yl)-3-methylpenta-2,4-dien-1-yl]phosphonate (**9**) (24.4 mg, 0.026 mmol) in THF (0.5 mL) was added *t*BuOK (0.079 mL, 20% in hexane, 0.131 mmol). After stirring for 30 min, a solution of (2*E*,4*E*,6*E*)-2,7-dimethylocta-2,4,6-triene-1,8-dial (**7**) (4.3 mg, 0.026 mmol) in THF (0.5 mL) was added. The mixture was stirred from −30 to 0 °C for 1 h. Then, a saturated aqueous solution of NH_4_Cl was added, and the mixture was extracted with a 50:50 v/v Et_2_O/CH_2_Cl_2_ mixture. The combined organic layers were washed with a saturated aqueous solution of NaHCO_3_ and dried (Na_2_SO_4_), and the solvent was evaporated. Purification by column chromatography (C18-silica gel, gradient from MeOH to 70:30 v/v MeOH/Et_2_O) afforded 10.2 mg (65%) of an orange solid identified as auroxanthin (**6**) as a 3:1 mixture of 8*R*,8′*R*/8*R*,8′*S* diastereoisomers. This mixture was further purified by HPLC (C30 Develosil-Nomura chemical column, MeOH, 3.5 mL/min, detection at 430 and 400 nm), to provide the expected pure product.

#### Data for (3*S*,5*R*,8*R*,3′*S*,5′*R*,8′*R*)-auroxanthin (**6**)

^1^H NMR (400.13 MHz, CDCl_3_): δ 6.60 (app dd, *J* = 7.6, 3.1 Hz, 2H, H_15_ + H_15′_), 6.48 (dd, *J* = 15.1, 11.0 Hz, 2H, H_11_ + H_11′_), 6.31 (d, *J* = 15.1 Hz, 2H, H_12_ + H_12′_), 6.24 (d, *J* = 12.3 Hz, 2H, H_14_ + H_14′_), 6.18 (d, *J* = 11.8 Hz, 2H, H_10_ + H_10′_), 5.25 (s, 2H, H_8_ + H_8′_), 5.16 (s, 2H, H_7_ + H_7′_), 4.28–4.22 (m, 2H, H_3_ + H_3′_), 2.13 (dd, *J* = 13.8, 4.2 Hz, 2H, H_4A_ + H_4′A_), 1.98 (dd, *J* = 13.8, 4.4 Hz, 2H, H_4B_ + H_4′B_), 1.94 (s, 6H, H_20_ + H_20′_), 1.79–1.93 (m, 2H, H_2A_ + H_2′A_), 1.71 (s, 6H, H_19_ + H_19′_), 1.62 (s, 6H, H_18_ + H_18′_), 1.51 (dd, *J* = 14.1 Hz, 3.4 Hz, 2H, H_2B_ + H_2′B_), 1.33 (s, 6H, H_16_ + H_16′_), 1.17 (s, 6H, H_17_ + H_17′_) ppm. ^1^H NMR (400.13 MHz, C_6_D_6_): δ 6.71 (dd, *J* = 15.0, 11.1 Hz, 2H, H_11_ + H_11′_), 6.66 (app dd, *J* = 7.9, 2.9 Hz, 2H, H_15_ + H_15′_), 6.49 (d, *J* = 15.0 Hz, 2H, H_12_ + H_12′_), 6.44 (d, *J* = 11.1 Hz, 2H, H_10_ + H_10′_), 6.29 (app d, *J* = 7.8 Hz, 2H, H_14_ + H_14′_), 5.32 (s, 2H, H_8_ + H_8′_), 5.14 (s, 2H, H_7_ + H_7′_), 3.80–3.73 (m, 2H, H_3_ + H_3′_), 2.05 (ddd, *J* = 13.5, 3.4, 1.5 Hz, 2H, H_4A_ + H_4′A_), 1.93 (dd, *J* = 13.5, 4.1 Hz, 2H, H_4B_ + H_4′B_), 1.87 (s, 6H, H_20_ + H_20′_), 1.81 (s, 12H, H_19_ + H_19′_ + H_18_ + H_18′_), 1.47 (ddd, *J* = 14.2, 3.6, 1.6 Hz, 2H, H_2A_ + H_2′A_), 1.35 (s, 6H, H_16_ + H_16′_), 1.17 (dd, *J* = 14.1 Hz, 3.6 Hz, 2H, H_2B_ + H_2′B_), 1.04 (s, 6H, H_17_ + H_17′_) ppm. ^13^C NMR (101 MHz, C_6_D_6_): δ 154.7 (s), 139.0 (s), 137.9 (d), 136.4 (s), 132.9 (d), 130.5 (d), 127.2 (d), 125.0 (d), 120.2 (d), 87.9 (d), 87.0 (s), 67.7 (d), 47.7 (t), 46.7 (t), 34.0 (s), 31.7 (q), 29.3 (q), 28.8 (q), 13.0 (q), 12.9 (q) ppm. HRMS (ESI^+^): calcd for C_40_H_47_O_4_ ([M + H]^+^), 601.4251; found, 601.4245. UV (MeOH) (ε, M^–1^ cm^–1^): λ_max_ 378 (10 940), 399 (11 340), 424 (5520).

#### Data for (3*S*,5*R*,8*R*,3′*S*,5′*R*,8′*S*)-auroxanthin (**6**)

The data matched those described previously.^27,28^

### Synthesis of (5′*R*,8′*R*)-Echinenone 5′,8′-Epoxide (**2a**/**2b**)

#### Diethyl (2*E*,4*E*)-3-Methyl-5-(3-hydroxy-2,6,6-trimethylcyclohex-3-methylpenta-2,4-dien-1-yl)phosphonate (**16**)

Following the described procedure for the Stille coupling, the reaction of diethyl (*E*)-(3-iodobut-2-en-1-yl)phosphonate (**12**) (150 mg, 0.472 mmol), (*E*)-2,4,4-trimethyl-3-(2-(tributylstannyl)vinyl)cyclohex-2-en-1-ol (**15**)^51^ (179 mg, 0.542 mmol), [NBu_4_][Ph_2_PO_2_] (303 mg, 0.66 mmol), CuTC (162 mg, 0.849 mmol), and Pd(PPh_3_)_4_ (33 mg, 0.028 mmol) in DMF (9.4 mL) at 25 °C for 2 h afforded, after purification by flash-column chromatography (silica gel, from 70:30 to 0:100 v/v hexane/EtOAc), 167 mg (99%) of a pale yellow oil identified as diethyl (2*E*,4*E*)-3-methyl-5-(3-hydroxy-2,2,6-trimethylcyclohex-3-methylpenta-2,4-dien-1-yl)phosphonate (**16**). ^1^H NMR (400.16 MHz, C_6_D_6_): δ 6.19 (d, *J* = 16.2 Hz, 1H, H_4_), 6.09 (d, *J* = 16.3 Hz, 1H, H_5_), 5.60 (app q, *J* = 7.7 Hz, 1H, H_2_), 4.00–3.86 (m, 5H, H_3′_ + 2×OCH_2_CH_3_), 2.61 (dd, *J*_H*–*P_ = 23.0, *J* = 8.0 Hz, 2H, 2H_1_), 1.90 (s, 3H, C_2′_-CH_3_), 1.82–1.76 (m, 1H, H_4′_), 1.72 (d, *J*_H*–*P_ = 3.8 Hz, 3H, C_3_–CH_3_), 1.74–1.64 (m, 2H, H_4′_ + H_5′_), 1.36–1.28 (m, 1H, H_5′_), 1.04 (t, *J* = 7.1 Hz, 6H, 2×OCH_2_CH_3_), 1.04 (s, 3H, C_6′_-CH_3_) 0.98 (s, 3H, C_6′_-CH_3_) ppm. ^13^C NMR (100.63 MHz, C_6_D_6_): δ 140.8 (s), 138.4 (d, *J*_C*–*P_ = 5.6 Hz), 137.9 (s, *J*_C*–*P_ = 14.5 Hz), 131.1 (s), 125.6 (d, *J*_C*–*P_ = 4.8 Hz), 120.2 (d, *J*_C*–*P_ = 12.2 Hz), 69.9 (d), 61.8 (t, *J*_C*–*P_ = 6.5 Hz, 2×), 35.2 (t), 34.9 (s), 29.2 (t), 29.1 (q) 27.8 (q), 27.6 (t, *J*_C*–*P_ = 140.5 Hz), 18.8 (q), 16.5 (q, *J*_C*–*P_ = 5.6 Hz, 2×), 12.5 (q, *J*_C*–*P_ = 2.4 Hz) ppm. IR (NaCl): ν 3500–3000 (br, O–H), 2955 (s, C–H), 2864 (s, C–H), 1241 (m, P=O), 1023 (s, P–O–C) cm^–1^. MS (ESI^+^-TOF): *m*/*z* (%) 357 ([M + H]^+^, 76), 340 (76), 339 (100), 242 (73). HRMS (ESI^+^): calcd for C_19_H_34_O_4_P ([M + H]^+^), 357.2187; found, 357.2189.

#### Diethyl ((2*E*,4*E*)-5-(3-((*tert*-Butyldimethylsilyl)oxy)-2,6,6-trimethylcyclohex-1-en-1-yl)-3-methylpenta-2,4-dien-1-yl)phosphonate (**11**)

To a cooled (0 °C) solution of diethyl (2*E*,4*E*)-3-methyl-5-(3-hydroxy-2,6,6-trimethylcyclohex-3-methylpenta-2,4-dien-1-yl)phosphonate (**16**) (50 mg, 0.14 mmol) and imidazole (24 mg, 0.35 mmol) in DMF (0.5 mL) was added dropwise a solution of *tert*-butyl chlorodimethylsilane (32 mg, 0.21 mmol) in DMF (0.5 mL). After stirring for 7 h at 25 °C, the reaction mixture was diluted with water and extracted with ethyl acetate (4×). The combined organic layers were washed with H_2_O (5×) and dried (Na_2_SO_4_), and the solvent was evaporated. The residue was purified by flash-column chromatography (silica gel, gradient from 80:20 to 0:100 v/v hexane/EtOAc) to afford 54 mg (82%) of a colorless oil identified as diethyl ((2*E*,4*E*)-5-(3-((*tert*-butyldimethylsilyl)oxy)-2,6,6-trimethylcyclohex-1-en-1-yl)-3-methylpenta-2,4-dien-1-yl)phosphonate (**11**). ^1^H NMR (400.16 MHz, CDCl_3_): δ 6.06 (d, *J* = 16.3 Hz, 1H, H_4_), 6.01 (d, d, *J* = 16.3 Hz, 1H, H_5_), 5.42 (app q, *J* = 8.0 Hz, 1H, H_2_), 4.13–4.03 (m, 4H, 2×OCH_2_CH_3_), 3.99 (t, *J* = 5.4 Hz, 1H, H_3′_), 2.70 (dd, *J*_H*–*F_ = 22.9 Hz, *J* = 8.0 Hz, 2H, 2H_1_), 1.80 (d, *J*_H*–*F_ = 3.8 Hz, 3H, C_3_–CH_3_), 1.78–1.73 (m, 1H, H_4′_),1.69 (s, 3H, C_2′_-CH_3_), 1.67–1.59 (m, 2H, H_4′_ + H_5′_), 1.39–1.31 (m, 1H, H_5′_), 1.29 (t, *J* = 7.1 Hz, 6H, 2×OCH_2_CH_3_), 1.00 (s, 3H, C_6′_-CH_3_), 0.94 (s, 3H, C_6′_-CH_3_), 0.89 (s, 9H, SiMe_2_(*t*Bu)), 0.07 (s, 3H, SiMe_2_(*t*Bu)), 0.06 (s, 3H, SiMe_2_(*t*Bu)) ppm. ^13^C NMR (100.63 MHz, CDCl_3_): δ 140.1 (s, *J*_C*–*P_ = 1.6 Hz), 138.2 (s, *J*_C*–*P_ = 14.8 Hz), 137.7 (d, *J*_C*–*P_ = 5.6 Hz), 131.1 (s), 125.9 (d, *J*_C*–*P_ = 4.4 Hz), 118.8 (d, *J*_C*–*P_ = 12.3 Hz), 71.3 (d), 62.1 (t, *J*_C*–*P_ = 6.8 Hz), 35.4 (t), 34.7 (s), 29.4 (t), 28.6 (q), 28.4 (q), 27.1 (t, *J*_C*–*P_ = 140.1 Hz), 26.1 (q, 3×), 18.5 (q), 18.3 (s), 16.6 (q, *J*_C*–*P_ = 6.0 Hz, 2×), 12.6 (q, *J*_C*–*P_ = 2.5 Hz), −4.1 (q), −4.5 (q) ppm. IR (NaCl): ν 2929 (s, C–H), 2857 (s, C–H), 1251 (m, P=O), 1031 (s, P–O–C) cm^–1^. MS (ESI^+^-TOF): *m*/*z* (%) 340 (21) 339 (100), 279 (21). HRMS (ESI^+^): calcd for C_25_H_48_O_4_PSi ([M + H]^+^), 471.3059; found, 471.3054.

#### (2*E*,4*E*,6*E*,8*E*,10*E*,12*E*)-13-(3-*tert*-Butyldimethylsilyl)oxyl)-2,6,6-trimethylcyclohex-1-en-1-yl)-2,7,11-trimethyltrideca-2,4,6,8,10,12-hexaenal (**17**)

A 1 M solution of NaHMDS in THF (0.097 mL, 0.097 mmol) was added to a cold (−78 °C) solution of (2*E*,4*E*,6*E*)-2,7-dimethylocta-2,4,6-trienedial (**7**) (16 mg, 0.097 mmol) and diethyl ((2*E*,4*E*)-5-(3-((*tert*-butyldimethylsilyl)oxy)-2,6,6-trimethylcyclohex-1-en-1-yl)-3-methylpenta-2,4-dien-1-yl)phosphonate (**11**) (41.6 mg, 0.088 mmol) in THF (3.3 mL). The reaction mixture was allowed to warm up slowly until reaching −30 °C. Then, a saturated NH_4_Cl solution was added, and the mixture was extracted with CH_2_Cl_2_ (3×). The combined organic layers were washed with a saturated NaHCO_3_ solution and dried (Na_2_SO_4_), and the solvent was evaporated. The residue was purified by flash-column chromatography (C18-silica gel, gradient from CH_3_CN to 50:50 v/v CH_3_CN/CH_2_Cl_2_) to afford 38 mg (89%) of an orange oil identified as (2*E*,4*E*,6*E*,8*E*,10*E*,12*E*)-13-(3-*tert*-butyldimethylsilyl)oxyl)-2,6,6-trimethylcyclohex-1-en-1-yl)-2,7,11-trimethyltrideca-2,4,6,8,10,12-hexaenal (**17**). ^1^H NMR (400.16 MHz, C_6_D_6_): δ 9.46 (s, 1H, CHO), 6.81 (dd, *J* = 15.0, 11.4 Hz, 1H, H_9_), 6.62 (dd, *J* = 11.8, 13.2 Hz, 1H, H_5_), 6.44 (d, *J* = 11.5 Hz, 1H, H_3_), 6.39 (dd, *J* = 10.8, 13.2 Hz, 1H, H_4_), 6.38 (d, *J* = 16.0, 1H, H_12_), 6.30 (d, *J* = 16.0, 1H, H_13_), 6.25 (d, *J* = 11.4 1H, H_10_), 6.09 (d, *J* = 11.8 Hz, 1H, H_6_), 4.05 (t, *J* = 4.9 Hz, 1H, H_3′_), 2.01 (s, H, C_2′_-CH_3_), 1.90 (s, 3H, C_11_–CH_3_), 1.82 (s, 3H, C_2_-CH_3_), 1.78 (s, 3H, C_7_-CH_3_), 1.81–1.72 (m, 2H), 1.44–1.35 (m, 2H), 1.12 (s, 3H, C_6′_-CH_3_), 1.09 (s, 3H, C_6′_-CH_3_), 1.03 (s, 9H, SiMe_2_(*t*Bu)), 0.15 (s, 3H, SiMe_2_(*t*Bu)), 0.13 (s, 3H, SiMe_2_(*t*Bu)) ppm. ^13^C NMR (100.63 MHz, C_6_D_6_): δ 193.2 (d), 147.6 (d), 140.9 (s), 140.8 (s), 139.1 (d), 137.6 (s), 137.4 (d), 137.3 (s), 137.0 (d), 131.9 (d), 131.8 (s), 131.7 (d), 128.1 (d), 127.9 (d), 71.6 (d), 35.6 (t), 35.1 (s), 29.8 (t), 28.9 (q), 28.5 (q), 26.2, (q, 3×) 19.2 (q), 18.4 (s), 12.9 (q), 12.8 (q), 9.7 (q), −4.0 (q), −4.5 (q) ppm. IR (NaCl): ν 2925 (s, C–H), 2856 (s, C–H), 1739 (m, C=O), 1082 (s) cm^–1^. MS (ESI^+^-TOF): *m*/*z* (%) 481 ([M + H]^+^, 10), 350 (29), 349 (100), 279 (11). HRMS (ESI^+^): calcd for C_31_H_49_O_2_Si ([M + H]^+^), 481.3493; found, 481.3496.

#### 4-Hydroxyechinenone-5′,8′-epoxide (**19**)

To a cooled (−30 °C) solution of diethyl (2*E*,4*E*)-3-methyl-5-((1*S*,6*R*)-2,2,6-trimethyl-7-oxabicyclo[4.1.0]heptan-1-yl)penta-2,4-dien-1-yl]phosphonate (**8**) (29.8 mg, 0.100 mmol) in THF (1.4 mL) was added *t*BuOK (0.056 mL, 0.092 mmol, 20% w/w in THF). After stirring for 30 min, a solution of (2*E*,4*E*,6*E*,8*E*,10*E*,12*E*)-13-(3-*tert*-butyldimethylsilyl)oxyl)-2,6,6-trimethylcyclohex-1-en-1-yl)-2,7,11-trimethyltrideca-2,4,6,8,10,12-hexaenal (**17**) (37.0 mg, 0.077 mmol) in THF (1.7 mL) was added via cannula, and the resulting mixture was stirred from −30 to 0 °C for 2 h. Then, a saturated aqueous solution of NH_4_Cl was added, and the mixture was extracted with a 50:50 v/v Et_2_O/CH_2_Cl_2_ mixture (3×). The combined organic layers were washed with a saturated aqueous solution of NaHCO_3_ and dried (Na_2_SO_4_), and the solvent was evaporated. The residue was purified by flash-column chromatography (C18-silica gel, gradient from MeOH to 50:50 v/v MeOH/Et_2_O) to afford 24.5 mg (47%) of an orange solid identified as *tert*-butyldimethyl((2,4,4-trimethyl-3-((1*E*,3*E*,5*E*,7*E*,9*E*,11*E*,13*E*,15*E*)-3,7,12-trimethyl-16-((2*R*,7a*R*)-4,4,7a-trimethyl-2,4,5,6,7,7a-hexahydrobenzofuran-2-yl)heptadeca-1,3,5,7,9,11,13,15-octaen-1-yl)cyclohex-2-en-1-yl)oxyl)silane (**18**), which was used in the next step without further purification.

To a solution of *tert*-butyldimethyl((2,4,4-trimethyl-3-((1*E*,3*E*,5*E*,7*E*,9*E*,11*E*,13*E*,15*E*)-3,7,12-trimethyl-16-((2*R*,7a*R*)-4,4,7a-trimethyl-2,4,5,6,7,7a-hexahydrobenzofuran-2-yl)heptadeca-1,3,5,7,9,11,13,15-octaen-1-yl)cyclohex-2-en-1-yl)oxyl)silane (**18**) (24.5 mg, 0.036 mmol) in THF (0.250 mL) was added TBAF (0.072 mL, 0.072 mmol, 1 M in THF), and the reaction mixture was stirred for 14 h at 25 °C. After this time, an additional 2 equiv of TBAF (0.072 mL, 0.072 mmol, 1 M in THF) was added, and the mixture was stirred for a further 3 h at 25 °C. The reaction mixture was poured over a saturated aqueous solution of NaHCO_3_ and extracted with 50:50 v/v Et_2_O/CH_2_Cl_2_ (3×). The combined organic layers were dried (Na_2_SO_4_), and the solvent was evaporated. The residue was purified by flash-column chromatography (CN-silica gel, gradient from hexane to 90:10 v/v hexane/acetone) to afford 19.9 mg (98%) of an orange solid identified as 4-hydroxyechinenone-5′,8′-epoxide (**19**). ^1^H NMR (400.16 MHz, C_6_D_6_): δ 6.77 (dd, *J* = 14.9, 11.4 Hz, 1H, H_11′_), 6.71 (dd, *J* = 15.0, 11.0 Hz, 1H, H_11_), 6.70–6.64 (m, 2H, H_15_ + H_15′_), 6.49 (d, *J* = 15.0 Hz, 1H, H_12′_), 6.48 (d, *J* = 15.0 Hz, 1H, H_12_), 6.45 (d, *J* = 11.0 Hz, 1H, H_10_), 6.37 (d, *J* = 16.1 Hz, 1H, H_7_), 6.35–6.26 (m, 3H, H_14_ + H_14′_ + H_10′_), 6.22 (d, *J* = 16.1 Hz, 1H, H_8_), 5.33 (s, 1H, H_8′_), 5.11 (s, 1H, H_7′_), 3.84 (br s, 1H, H_4_), 2.07–1.96 (m, 1H, H_4′_), 1.95 (s, 3H), 1.91 (s, 3H), 1.87 (s, 6H), 1.85 (s, 3H), 1.80–1.70 (m, 1H, H_4′_), 1.68–1.59 (m, 4H), 1.49 (s, 3H), 1.47–1.39 (m, 2H), 1.37–1.27 (m, 1H), 1.15–1.09 (m, 1H), 1.09 (s, 3H), 1.05 (s, 3H), 1.04 (s, 3H), 0.98 (s, 3H) ppm. ^13^C NMR (100.63 MHz, C_6_D_6_): δ 154.7 (s), 141.3 (s), 139.41 (d), 139.37 (s), 138.5 (d), 137.8 (d), 136.7 (s), 136.5 (s), 135.5 (s), 133.5 (d), 132.9 (d), 132.6 (d), 131.0 (s), 130.9 (d), 130.4 (d), 127.2 (d), 126.1 (d), 125.3 (d), 125.2 (d), 119.7 (d), 88.0 (d), 87.6 (s), 70.1 (d), 41.8 (t), 41.7 (t), 35.1 (t), 35.0 (s), 34.6 (s), 30.9 (q), 29.3 (q), 29.1 (t), 27.9 (q), 26.4 (q), 26.1 (q), 20.7 (t), 19.0 (q), 13.1 (q), 12.9 (q), 12.9 (q), 12.8 (q) ppm.

#### (5′*R*,8′*R*)- and (5′*R*,8′*S*)-Echinenone-5′,8′-epoxide (**2a**/**2b**)

To a solution of 2,4,4-trimethyl-3-((1*E*,3*E*,5*E*,7*E*,9*E*,11*E*,13*E*,15*E*)-3,7,12-trimethyl-16-((2*R*,7a*R*)-4,4,7a-trimethyl-2,4,5,6,7,7a-hexahydrobenzofuran-2-yl)heptadeca-1,3,5,7,9,11,13,15-octaen-1-yl)cyclohex-2-en-1-ol (**19**) (10.9 mg, 0.019 mmol) in DMSO (0.200 mL) was added freshly prepared IBX (10.7 mg, 0.038 mmol), and the reaction mixture was stirred for 24 h at 25 °C. After this time an additional equivalent of IBX (5.4 mg, 0.019 mmol) was added, and the mixture was further stirred for 16 h at the same temperature. The reaction mixture was diluted with water and extracted with Et_2_O (3×). The combined organic layers were washed with a saturated aqueous solution of NaHCO_3_, dried over anhydrous Na_2_SO_4_, filtered, and concentrated. The residue was purified by flash-column chromatography (C18-silica gel, gradient from MeOH to 50:50 v/v MeOH/Et_2_O) to afford 8.0 mg (74%) of an orange solid identified as echinenone-5′,8′-epoxide (**2a/2b**) as a 4:1 mixture of the 5′*R*,8′*R*/5′*R*,8′*S* isomers. This mixture was further purified by HPLC (C30 Develosil-Nomura chemical column, MeOH, 3.5 mL/min, detection at 430 and 400 nm) to provide the expected pure products.

#### Data for (5′*R*,8′*R*)-echinenone-5′,8′-epoxide (**2a**)

^1^H NMR (400.16 MHz, CDCl_3_): δ 6.66 (dd, *J* = 14.4, 10.6 Hz, 1H), 6.63–6.57 (m, 2H), 6.51 (dd, *J* = 15.0, 11.0 Hz, 1H), 6.42 (d, *J* = 14.8 Hz, 1H), 6.36 (d, *J* = 16.1 Hz, 1H), 6.30–6.23 (m, 2H), 6.22 (d, *J* = 16.0 Hz, 1H), 6.22 (d, *J* = 10.6 Hz, 1H), 6.19 (d, *J* = 11.0 Hz, 1H), 5.17 (s, 1H), 5.15 (s, 1H), 2.50 (t, *J* = 6.8 Hz, 2H), 2.00 (s, 3H), 1.97 (s, 3H), 1.95 (s, 3H), 1.87 (s, 3H), 1.85 (t, *J* = 6.8 Hz, 2H), 1.74 (s, 3H), 1.43 (s, 3H), 1.19 (s, 6H), 1.15 (s, 3H), 1.10 (s, 3H) ppm. ^1^H NMR (400.16 MHz, C_6_D_6_): δ 6.72 (dd, *J* = 15.0, 11.1 Hz, 1H, H_11′_), 6.71 (dd, *J* = 14.8, 11.0 Hz, 1H, H_11_), 6.70–6.61 (m, 2H, H_15_ + H_15′_), 6.51 (d, *J* = 14.8 Hz, 1H, H_12′_), 6.48 (d, *J* = 15.0 Hz, 1H, H_12_), 6.46 (d, *J* = 11.1 Hz, 1H, H_10′_), 6.38 (d, *J* = 16.0 Hz, 1H, H_8_), 6.35 (d, *J* = 11.0 Hz, 1H, H_14′_), 6.29 (d, *J* = 11.0 Hz, 2H, H_10_ + H_14_), 6.14 (d, *J* = 16.0 Hz, 1H, H_7_), 5.33 (s, 1H, H_8′_), 5.11 (s, 1H, H_7′_), 2.41 (t, *J* = 6.8 Hz, 2H, 2H_3_), 2.17 (s, 3H, 3H_18_), 2.00 (d, *J* = 12.5 Hz, 1H, H_4′_), 1.86 (s, 3H, 3H_20_), 1.85 (s, 6H, 3H_20_ + 3H_19′_), 1.82 (s, 3H, 3H_19_), 1.74–1.65 (m, 1H, H_4′_), 1.52–1.46 (m, 2H, 2H_2_), 1.49 (s, 3H, 3H_18′_), 1.46–1.40 (m, 2H, H_3′_), 1.34–1.27 (m, 1H, H_2′_), 1.14–1.07 (m, 1H, H_2′_), 1.05 (s, 3H, 3H_16′_), 0.98 (s, 3H, 3H_17′_), 0.95 (s, 6H, 3H_16_ + 3H_17_) ppm. ^13^C NMR (100.63 MHz, C_6_D_6_): δ 197.3 (s), 159.5 (s), 154.8 (s), 141.4 (d), 139.9 (d), 139.6 (s), 137.7 (d), 137.2 (s), 136.2 (s), 134.9 (d), 134.7 (s), 134.4 (d), 132.7 (d), 131.4 (d), 130.4 (s), 130.2 (d), 127.2 (d), 125.5 (d), 124.8 (d), 124.4 (d), 119.7 (d), 88.0 (d), 87.6 (s), 41.8 (t), 41.7 (t), 37.6 (t), 35.6 (s), 34.6 (t), 30.9 (q), 27.6 (q, 2×), 26.4 (q), 26.1 (q), 20.7 (t), 14.4 (q), 13.1 (q), 13.0 (q), 12.8 (q), 12.5 (q) ppm. MS (ESI^+^-TOF): *m*/*z* (%) 567 ([M + H]^+^, 100). HRMS (ESI^+^): calcd for C_40_H_55_O_2_ ([M + H]^+^), 567.4195; found, 567.4197.

#### Data for (5′*R*,8′*S*)-echinenone-5′,8′-epoxide (**2b**)

^1^H NMR (400.16 MHz, CDCl_3_): δ 6.66 (dd, *J* = 14.4, 11.3 Hz, 1H), 6.63–6.57 (m, 2H), 6.52 (dd, *J* = 15.0, 11.0 Hz, 1H), 6.42 (d, *J* = 14.8 Hz, 1H), 6.36 (d, *J* = 16.0 Hz, 1H), 6.30–6.23 (m, 2H), 6.22 (d, *J* = 16.0 Hz, 1H), 6.22 (d, *J* = 11.0 Hz, 1H), 6.18 (d, *J* = 11.0 Hz, 1H), 5.23 (s, 1H), 5.07 (s, 1H,), 2.50 (t, *J* = 6.8 Hz, 2H), 2.00 (s, 3H), 1.97 (s, 3H), 1.95 (s, 3H), 1.87 (s, 3H), 1.85 (t, *J* = 6.8 Hz, 2H), 1.80 (s, 3H), 1.46 (s, 3H), 1.19 (s, 6H), 1.17 (s, 3H,), 1.11 (s, 3H) ppm. ^1^H NMR (400.16 MHz, C_6_D_6_): δ 6.72 (dd, *J* = 15.0, 10.9 Hz, 1H, H_11′_), 6.71 (dd, *J* = 14.9, 11.0 Hz, 1H, H_11_), 6.70–6.61 (m, 2H, H_15_ + H_15′_), 6.51 (d, *J* = 10.9 Hz, 1H, H_10′_), 6.50 (d, *J* = 15.0 Hz, 2H, H_12′_ + H_12_), 6.38 (d, *J* = 16.0 Hz, 1H, H_8_), 6.35 (d, *J* = 11.0 Hz, 1H, H_14′_), 6.29 (d, *J* = 11.0 Hz, 2H, H_10_ + H_14_), 6.14 (d, *J* = 16.0 Hz, 1H, H_7_), 5.25 (s, 1H, H_8′_), 5.19 (d, *J* = 1.9 Hz, 1H, H_7′_), 2.41 (t, *J* = 6.8 Hz, 2H, 2H_3_), 2.17 (s, 3H, 3H_18_), 1.98 (d, *J* = 12.1 Hz, 1H, H_4′_), 1.88 (s, 3H, 3H_20_), 1.87 (s, 3H, 3H_20_), 1.85 (s, 3H, 3H_19′_), 1.82 (s, 3H, 3H_19_), 1.68–1.59 (m, 1H, H_4′_), 1.54 (s, 3H, 3H_18′_), 1.51–1.47 (m, 2H, 2H_2_), 1.46–1.39 (m, 2H, H_3′_), 1.36–1.27 (m, 1H, H_2′_), 1.14–1.03 (m, 1H, H_2′_), 1.08 (s, 3H, 3H_16′_), 0.99 (s, 3H, 3H_17′_), 0.95 (s, 6H, 3H_16_ + 3H_17_) ppm. ^13^C NMR (100.63 MHz, C_6_D_6_): δ 197.3 (s), 159.5 (s), 154.1 (s), 141.4 (d), 140.2 (s), 139.9 (d), 137.6 (d), 137.2 (s), 136.2 (s), 134.9 (d), 134.7 (s), 134.4 (d), 132.6 (d), 131.5 (d), 130.4 (s), 130.2 (d), 126.1 (d), 125.6 (d), 124.8 (d), 124.4 (d), 118.6 (d), 88.4 (d), 88.0 (s), 42.3 (t), 42.1 (t), 37.6 (t), 35.6 (s), 35.1 (s), 34.6 (t), 30.8 (q), 27.9 (q), 27.6 (q, 2×), 25.7 (q), 21.0 (t), 14.4 (q), 13.7 (q), 13.0 (q), 12.8 (q), 12.5 (q) ppm.

## Supporting Information Available

Synthesis procedures for additional precursors of auroxanthin and study of their reactivity under HWE reaction conditions; ^1^H and ^13^C NMR spectra, including NOE effects for structural determination, for all compounds described in the text (PDF)
