# Supplementary material for: Stereoselective Synthesis of Bisfuranoxide (Aurochrome, Auroxanthin) and Monofuranoxide (Equinenone 5′,8′-Epoxide) Carotenoids by Double Horner–Wadsworth–Emmons Reaction
Source: J Nat Prod. 2022 Sep 19;85(10):2302–11. doi: 10.1021/acs.jnatprod.2c00475 (PMC9693700; doi:10.1021/acs.jnatprod.2c00475)
Supplement: Supplementary file 1 — np2c00475_si_001.pdf [file np2c00475_si_001.pdf]

Supporting Information.

Stereo- and Enantioselective Synthesis of Bisfuranoxide (Aurochrome, Auroxanthin) and Monofuranoxide (Equinenone 5',8´-epoxide) Carotenoids by Double Horner-Wadsworth-Emmons Reaction

Aurea Rivas, Marta Castiñeira, Rosana Álvarez,* Belén Vaz,* and Angel R. de Lera*

Department of Organic Chemistry and Center for Biomedical Research (CINBIO), IBIV, Universidade de Vigo, 36310 Vigo, Spain

- Synthesis procedures for additional precursors of auroxanthin and study of their reactivity under HWE reaction conditions. **S3**

- ^{1}H- and ^{13}C-NMR spectra, including NOE effects for structural determination:

NMR spectra of diethyl (2*E*,4*E*)-3-methyl-5-((1*S*,6*R*)-2,2,6-trimethyl-7-oxabicyclo[4.1.0]heptan-1-yl)-penta-2,4-dien-1-yl]phosphonate **8** **S9**
NMR spectra of (8*R*,8'*R*)-aurochrome **4** in C_6D_6 **S11**
NOESY-1d spectra (C_6D_6) of (8*R*,8'*R*)-aurochrome **4** **S16**
^{1}H-NMR spectrum of (8*R*,8'*R*)-aurochrome **4** in $CDCl_3$ **S18**

NMR spectra of diethyl (2*E*,4*E*,1'*S*,4'*S*,6'*R*)-[5-(4-hydroxy-2,2,6-trimethyl-7-oxabicyclo[4.1.0]heptan-1-yl)-3-methylpenta-2,4-dien-1-yl] phosphonate **9** **S19**
NMR spectra of diethyl (2*E*,2'*R*,6'*S*,7'a*R*)-[3-(6-hydroxy-4,4,7a-trimethyl-2,4,5,6,7,7a-hexahydrobenzofuran-2-yl)-3-methylprop-2-en-1-yl]phosphonate **10** **S21**
NMR spectra of (2*R*,6*S*,7a*R*,2'*E*)-2-(4-diethoxyphosphorylbut-2-en-2-yl)-4,4,7a-trimethyl-2,4,5,6,7,7a-hexahydrobenzofuran-6-yl acetate **21** **S23**
NMR spectra of (1*R*,3*S*,6*S*,1'*E*,3'*E*)-5-diethoxyphosphoryl-3-methylpenta-1,3-dien-1-yl-1,5,5-trimethyl-7-oxabicyclo[4.1.0]heptan-3-yl acetate **20** **S25**
NMR spectra of (1*S*,3*R*,4*Z*,2'*E*,4'*E*)-4-(5'-diethoxyphosphoryl-3'-methylpenta-2',4'-dien-1'-ylidene)-3-hydroxy-3,5,5-trimethylcyclohexyl acetate **22** **S27**
NMR spectra of diethyl (1*E*,3*E*,5*Z*,2*R*,4'*S*)-5-(-2',4'-dihydroxy-2,6,6-trimethylcyclohexylidene)-3-methylpenta-1,3-dien-1-yl)phosphonate **23** **S29**
NMR spectra of (8*R*,8'*R*)-auroxanthin **6** in C_6D_6 **S31**
NOESY-1d spectra (C_6D_6) of (8*R*,8'*R*)- auroxanthin **6** **S36**
^{1}H-NMR spectrum of (8*R*,8'*R*)-auroxanthin **6** in $CDCl_3$ **S39**

NMR spectra of diethyl (2*E*,4*E*)-3-methyl-5-(3-hydroxy-2,6,6-trimethylcyclohex-3-methylpenta-2,4-dien-1-yl)phosphonate **15** **S40**
NMR spectra of diethyl ((2*E*,4*E*)-5-(3-((*tert*-butyldimethylsilyl)oxy)-2,6,6-trimethylcyclohex-1-en-1-yl)-3-methylpenta-2,4-dien-1-yl)phosphonate **11** **S42**

NMR spectra of (2*E*,4*E*,6*E*,8*E*,10*E*,12*E*)-13-(3-*tert*-butyldimethylsilyl)oxyl)-2,6,6-trimethylcyclohex-1-en-1-yl)-2,7,11trimethyltrideca-2,4,6,8,10,12-hexaenal **17** **S45**
NMR spectra of 4-hydroxy-echinenone-5',8'-epoxide **19** **S46**
NMR spectra of (5'*R*,8'*R*)-echinenone-5',8'-epoxide **2a** **S48**
NOESY-1d spectra (C_6D_6) of (5'*R*,8'*R*)-echinenone-5',8'-epoxide **2a** **S53**
NMR spectra of (5'*R*,8'*S*)-echinenone-5',8'-epoxide **2b** **S55**
NOESY-1d spectra (C_6D_6) of (5'*R*,8'*S*)-echinenone-5',8'-epoxide **2b** **S58**
Comparison of ^{1}H-NMR spectra of **2a** and **2b** ($CDCl_3$) **S60**

In efforts to explore alternative procedures for the two-fold HWE reaction, the C5,C8-dihydrofuran derivative **10** was prepared from **9** in 92% yield. The process was however accompanied by partial degradation of the substrate, which might reflect its sensitivity to acid, affording unstable cationic species. Finally, the secondary alcohol of either **9** or **10** was protected as the corresponding acetates **20** or **21** (Ac_2O, pyridine, CH_2Cl_2, 25 °C, 68% and 84% yield, respectively), with the purpose to avoid undesired competing reactions during the HWE condensation with dialdehyde **7** (Scheme S1).

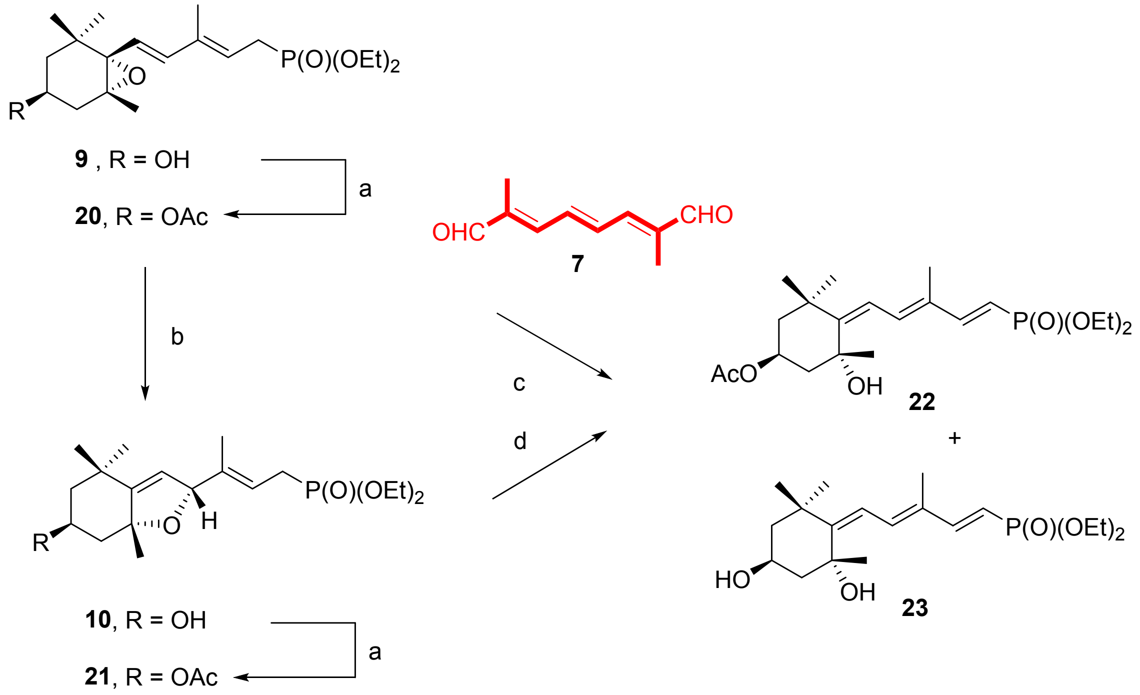

Scheme S1. Reagents and reaction conditions: (a) Ac_2O, pyridine, DMAP, CH_2Cl_2, 25 °C, 68% for **20**; 84% for **21**. (b). TFA, CH_2Cl_2, 0 °C, 92%. (c). **7**, NaHMDS, THF, -78 to 25 °C, 15h, quantitative yield from **16** (3:1 **22/23** ratio); traces from **10**. (d). NaOMe, MeOH, -30 °C, 70%.

The bidirectional HWE reaction of **7** and alkenyl phosphonates **10** or **21** followed by deprotection was also examined, but unfortunately auroxanthin (**6**) was not present, despite the expectation that the reaction conditions should generate the same intermediate.[1] Treatment of protected 5,8-dihydrofuranoxide allyl phosphonate **20** (2.2 equiv) with 2.5 equiv NaHMDS in THF) with C_{10}-trienedialdehyde **7** was inefficient, and the reactants were partially recovered, with just trace amounts of the 5,8-dihydrofuranoxide-pentaenal derived from the mono-HWE condensation being detected. Likewise, the use of the anion generated from preformed dihydrofuranoxide **10** (generated using 4.6 mol equivalents of *t*BuOK in THF from -30 °C to rt) led to extensive degradation and afforded only traces of the corresponding apocarotenoid[1] and recovered C_{10}-trienedialdehyde **7** accompanied by isomerized trienyl phosphonate (not shown). Using instead phosphonate **20**, a 1:1 mixture of trienyl phosphonate **22** and deprotected analogue **23** was obtained when using NaHMDS as base (THF, -78 to 0 °C). The **22/23** mixture was also obtained in a 3:1 ratio upon further heating to 25 °C the anion obtained from the treatment of acetylated dienyl phosphonate **21** with *t*BuOK in THF at -30 °C. Finally, when the allyl phosphonate **9** was treated with NaOMe in MeOH, the deprotected trienyl phosphonate **23**[1, 2] was obtained in 73% yield. It is tempting to suggest that the substrate with a smaller OH functionality at C_3 would be less disfavored to adopt the reactive conformation with the tertiary alkoxide at the equatorial position, leading to productive Bürgi-Dunitz trajectories to the C8-carbon of the unsaturated system. The intermediate could then evolve towards formation of the

furanoxide phosphonate **10**, and follow the desired HWE reaction manifold to provide the entire skeleton of auroxanthin (**6**) as depicted for aurochrome (**4**) in Scheme 3.[1, 2]

Diethyl (2*E*,2'*R*,6'*S*,7'a*R*)-[3-(6-Hydroxy-4,4,7a-trimethyl-2,4,5,6,7,7a-hexahydrobenzofuran-2-yl)-3-methylprop-2-en-1-yl]phosphonate (10). To a cooled (0 °C) solution of diethyl [(2*E*,4*E*,1'*S*,4'*S*,6'*R*)-5-(4-hydroxy-2,2,6-trimethyl-7-oxabicyclo[4.1.0]heptan-1-yl)-3-methylpenta-2,4-dien-1-yl]phosphonate (**9**) (23.3 mg, 0.065 mmol) in CH_2Cl_2 (3 mL) was added TFA (0.02 mL, 0.195 mmol), and the reaction mixture was allowed to reach room temperature. After stirring for 1 h at 25 °C, EtOAc and a saturated aqueous solution of $NaHCO_3$ were added until neutral pH. The aqueous layer was washed with EtOAc (3x) and the combined organic layers were washed with brine, dried (Na_2SO_4) and the solvent was removed. The residue was purified by column chromatography (silica gel, from 48:52 to 60:40 v/v acetone/CH_2Cl_2) to afford 21.5 mg (92%) of a product identified as diethyl (2*E*,2'*R*,6'*S*,7'*aR*)l-[3-(6-hydroxy-4,4,7a-trimethyl-2,4,5,6,7,7a-hexahydrobenzofuran-2-yl)-3-methylprop-2-en-1-yl]phosphonate (**10**). $[\alpha]_D^{24}$ +71 (*c* 0.19, MeOH). **^{1}H-NMR** (400.13 MHz, CD_3OD): δ 5.49 (q, J = 7.1 Hz, $^3J_{H-P}$ = 7.1 Hz, 1H, H_2), 5.28 (s, 1H, $H_{3'}$), 5.10 (s, 1H, $H_{2'}$), 4.14 - 4.03 (m, 5H, $H_{6'}$ + 2xO$\underline{CH_2}$CH$_3$), 2.68 (dd, J = 7.8 Hz, $^2J_{H-P}$ = 22.2 Hz, 2H, $2H_1$), 2.05 (dd, J = 13.7, 3.7 Hz, 1H, $H_{7'A}$), 1.91 (dd, J = 13.6, 4.4 Hz, 1H, $H_{7'B}$), 1.76 (ddd, J = 14.1, 4.7, 1.4 Hz, 1H, $H_{5'A}$), 1.59 (d, $^5J_{H-P}$ = 4.0 Hz, 3H, CH_3), 1.56 (s, 3H, CH_3), 1.45 (dd, J = 14.1, 3.6 Hz, 1H, $H_{5'B}$), 1.30 (t, J = 7.0 Hz, 6H, 2xOCH$_2\underline{CH_3}$), 1.30 (s, 3H, CH_3), 1.16 (s, 3H, CH_3) ppm. **^{13}C-NMR** (100.62 MHz, CD_3OD): δ 155.6 (s), 141.9 (s, $^3J_{C-P}$ = 14.3 Hz), 120.7 (d, $^5J_{C-P}$ = 3.1 Hz), 116.2 (d, $^2J_{C-P}$ = 11.7 Hz), 88.9 (d, $^4J_{C-P}$ = 3.1 Hz), 88.8 (s), 67.9 (d), 63.5 (t, $^2J_{C-P}$ = 5.4 Hz), 48.0 (t), 47.3 (t), 34.6 (s), 31.8 (q), 29.6 (q), 29.5 (q), 26.5 (t, $^1J_{C-P}$ = 142.3 Hz), 16.8 (q, $^3J_{C-P}$ = 6.4 Hz), 12.3 (q) ppm. **HRMS** (ESI$^+$): Calcd. for $C_{19}H_{34}O_5P$ ([M+H]$^+$), 373.2138; found, 373.2131. **IR (NaCl):** ν 3600-3100 (br, O-H), 2960 (s, C-H), 2922 (s, C-H), 2869 (w, C-H), 1242 (m, P=O), 1028 (s, P-O-C) cm^{-1}.

(2*R*,6*S*,7a*R*,2'*E*)-2-(4-Diethoxyphosphorylbut-2-en-2-yl)-4,4,7a-trimethyl-2,4,5,6,7,7a-hexahydrobenzofuran-6-yl Acetate (21). To a solution of diethyl (2*E*,2'*R*,6'*S*,7'*aR*)-3-(6-hydroxy-4,4,7a-trimethyl-2,4,5,6,7,7a-hexahydrobenzofuran-2-

yl)-3-methylprop-2-en-1-yl]phosphonate (**10**) (56.3 mg, 0.22 mmol) in CH_2Cl_2 (3.7 mL) were added pyridine (0.09 mL, 1.12 mmol), acetic anhydride (0.11 mL, 1.12 mmol) and DMAP (5.4 mg, 0.04 mmol). After stirring for 1 h at 25 °C, the reaction mixture was diluted with Et_2O and the organic layer was washed with a saturated aqueous solution of $CuSO_4$ (3x). The combined organic layers were dried (Na_2SO_4) and the solvent was evaporated. The residue was purified by flash-column chromatography (C-18 silica gel, from 50:50 to 100:0 v/v CH_3CN/H_2O) to afford 44.3 mg (68%) of a pale yellow oil identified as (2*R*,6*S*,7a*R*,2'*E*)-2-(4-diethoxyphosphorylbut-2-en-2-yl)-4,4,7a-trimethyl-2,4,5,6,7,7a-hexahydrobenzofuran-6-yl acetate (**21**). **$[\alpha]_D^{24}$** +58 (*c* 0.63, MeOH). **^{1}H-NMR** (400.13 MHz, CD_3OD): δ 5.53 (tapp q, J = 7.0 Hz, $^3J_{H\text{-}P}$ = 7.0 Hz, 1H, $H_{3'}$), 5.38 (s, 1H, H_3), 5.21 – 5.17 (m, 1H, H_6), 5.16 (s, 1H, H_2), 4.16 - 4.06 (m, 4H, 2xO$\underline{CH_2}$$CH_3$), 2.71 (dd, J = 8.0 Hz, $^2J_{H\text{-}P}$ = 22.0 Hz, 2H, $2H_{4'}$), 2.24 – 2.16 (m, 1H, H_{7A}), 2.07 (s, 3H, CH_3CO), 1.99 - 1.86 (m, 2H, H_{5A} + H_{7B}), 1.63 (d, $^4J_{H\text{-}P}$ = 3.6 Hz, 3H, $C_{2'}$-CH_3), 1.58 (s, 3H, CH_3), 1.56 - 1.50 (m, 1H, H_{5B}), 1.33 (t, J = 7.3 Hz, 6H, 2xO$\underline{CH_2}$$CH_3$), 1.21 (s, 3H, CH_3) ppm. **^{13}C-NMR** (100.62 MHz, CD_3OD): δ 171.8 (s), 154.6 (s), 141.7 (s, $^3J_{C\text{-}P}$ = 14.2 Hz), 121.5 (d, $^5J_{C\text{-}P}$ = 2.8 Hz), 116.3 (d, $^2J_{C\text{-}P}$ = 11.2 Hz), 88.8 (d, $^4J_{C\text{-}P}$ = 2.6 Hz), 88.0 (s), 71.4 (d), 63.5 (t, $^2J_{C\text{-}P}$ = 6.6 Hz, 2x), 44.9 (t), 44.3 (t), 34.8 (s), 31.7 (q), 28.7 (q), 28.6 (q), 26.4 (t, $^1J_{C\text{-}P}$ = 140.7 Hz), 21.4 (q), 16.8 (q, $^2J_{C\text{-}P}$ = 5.6 Hz), 12.4 (q, $^7J_{C\text{-}P}$ = 2.4 Hz) ppm. **HRMS** (ESI$^+$): Calcd. for $C_{21}H_{35}O_6P$ ([M+H]$^+$), 415.2244; found, 415.2230. **IR (NaCl):** ν 2963 (s, C-H), 2932 (s, C-H), 1737 (s, C=O), 1252 (s, P=O), 1027 (s, P-O-C) cm^{-1}.

(1*R*,3*S*,6*S*,1'*E*,3'*E*)-5-Diethoxyphosphoryl-3-methylpenta-1,3-dien-1-yl-1,5,5-trimethyl-7-oxabicyclo[4.1.0]heptan-3-yl Acetate (20). Following the described procedure for the acetylation of secondary alcohols, the reaction of diethyl (2*E*,4*E*,1'*S*,4'*S*,6'*R*)-[5-(4-hydroxy-2,2,6-trimethyl-7-oxabicyclo[4.1.0]heptan-1-yl)-3-methylpenta-2,4-dien-1-yl]phosphonate (**9**) (51.9 mg, 0.14 mmol), pyridine (0.06 mL, 0.7 mmol), acetic anhydride (0.07 mL, 0.7 mmol) and DMAP (3.4 mg, 0.03 mmol) in CH_2Cl_2 (2.3 mL) for 1 h at 25 °C produced, after column chromatography (silica gel-CN, from 100:0 to 90:10 hexane/EtOAc), 36.5 mg (84%) of a pale yellow solid identified as (1*R*,3*S*,6*S*,1'*E*,3'*E*)-5-diethoxyphosphoryl-3-methylpenta-1,3-dien-1-yl)-1,5,5-trimethyl-7-oxabicyclo[4.1.0]heptan-3-yl acetate (**20**). **$[\alpha]_D^{24}$** -8 (*c* 0.11, MeOH). **^{1}H-NMR** (400.13 MHz, CD_3OD): δ 6.26 (d, J = 15.7 Hz, 1H, $H_{2'}$), 5.96 (d, J = 15.7 Hz, 1H, $H_{1'}$), 5.49 (app q, J = 7.7 Hz, $^2J_{H\text{-}P}$ = 7.7 Hz, 1H, $H_{4'}$), 4.95 - 4.88 (m, 1H, H_3), 4.16

- 4.06 (m, 4H, 2xO$\underline{CH_2}$CH$_3$), 2.82 (dd, *J* = 8.2 Hz, $^2J_{H-P}$ = 23.2 Hz, 2H$_{5'}$), 2.37 (dd, *J* = 14.7, 5.5 Hz, 1H, H$_{2A}$), 2.02 (s, 3H, CH$_3$CO), 1.88 - 1.80 (m, 1H, H$_{2B}$), 1.85 (s, 3H, CH$_3$), 1.64 (d, *J* = 13.5 Hz, 1H, H$_{4A}$), 1.45 - 1.37 (m, 1H, H$_{4B}$), 1.35 - 1.30 (m, 6H, 2xOCH$_2\underline{CH_3}$), 1.19 (s, 6H, 2xCH$_3$), 0.98 (s, 3H, CH$_3$) ppm. **^{13}C-NMR** (100.62 MHz, CD$_3$OD): δ 172.2 (s), 138.6 (s, $^3J_{C-P}$ = 14.6 Hz), 138.0 (d, $^4J_{C-P}$ = 5.3 Hz), 124.7 (d, $^5J_{C-P}$ = 4.2 Hz), 120.9 (d, $^2J_{C-P}$ = 12.6 Hz), 71.6 (s), 69.0 (d), 67.3 (s), 63.6 (t, $^2J_{C-P}$ = 6.9 Hz, 2x), 42.9 (t), 37.8 (t), 35.9 (s), 29.2 (q), 27.1 (t, $^1J_{C-P}$ = 139.6 Hz), 25.6 (q), 21.3 (q), 20.3 (q), 16.7 (q, $^3J_{C-P}$ = 5.9 Hz, 2x), 12.9 (q, $^4J_{C-P}$ = 2.9 Hz) ppm. **HRMS** (ESI$^+$): Calcd. for $C_{21}H_{35}O_6P$ ([M+H]$^+$), 415.2244; found, 415.2240. **IR (NaCl):** ν 2969 (m, C-H), 2929 (m, C-H), 1736 (s, C=O), 1243 (s, P=O), 1028 (s, P-O) cm^{-1}.

(1*S*,3*R*,4*Z*,2'*E*,4'*E*)-4-(5'-Diethoxyphosphoryl-3'-methylpenta-2',4'-dien-1'-ylidene)-3-hydroxy-3,5,5-trimethylcyclohexyl Acetate (22) and Diethyl (1*E*,3*E*,5*Z*,2*R*,4'*S*)-5-(-2',4'-Dihydroxy-2,6,6-trimethylcyclohexylidene)-3-methylpenta-1,3-dien-1-yl)phosphonate (23). To a cooled (-78 °C) solution of (1*R*,3*S*,6*S*,1'*E*,3'*E*)-6-5-diethoxyphosphoryl-3-methylpenta-1,3-dien-1-yl-1,5,5-trimethyl-7-oxabicyclo[4.1.0]heptan-3-yl acetate (**20**) (18.2 mg, 0.04 mmol) in THF (0.7 mL) was added NaHMDS (0.05 mL, 1M in hexane, 0.05 mmol). After stirring for 30 min at -78 °C, a solution of (2*E*,4*E*,6*E*)-2,7-dimethylocta-2,4,6-triene-1,8-dial **7** (3.3 mg, 0.02 mmol) in THF (0.7 mL) was added. After being stirred at the same temperature for 1.5 h, the reaction mixture was stirred overnight at 25 °C. Then a saturated aqueous solution of NH$_4$Cl was added and the mixture was extracted with a 90:10 v/v EtOAc/CH$_2$Cl$_2$ mixture. The combined organic layers were washed with a saturated aqueous solution of NaHCO$_3$, dried (Na$_2$SO$_4$) and the solvent was evaporated. Purification by flash-column chromatography (silica gel-CN, from 50:50 to 0:100 v/v hexane/CH$_2$Cl$_2$) afforded a 1:1 mixture of two products identified as (1*S*,3*R*,4*Z*,2'*E*,4'*E*)-4-(5'-diethoxyphosphoryl-3'-methylpenta-2',4'-dien-1'-ylidene)-3-hydroxy-3,5,5-trimethylcyclohexyl acetate (**22**) and diethyl (1*E*,3*E*,5*Z*,2*R*,4'*S*)-5-(-2',4'-dihydroxy-2,6,6-trimethylcyclohexylidene)-3-methylpenta-1,3-dien-1-yl)phosphonate (**23**).

Data for (1*S*,3*R*,4*Z*,2'*E*,4'*E*)-4-(5'-diethoxyphosphoryl-3'-methylpenta-2',4'-dien-1'-ylidene)-3-hydroxy-3,5,5-trimethylcyclohexyl Acetate (22). $[\alpha]_D^{26}$ +41 (*c* 0.07, MeOH). **^{1}H NMR** (400.13 MHz, CD$_3$OD): δ 7.18 (dd, *J* = 17.1 Hz, $^2J_{H-P}$ = 22.2 Hz, 1H, H$_{4'}$), 6.98 (d, *J* = 12.0 Hz, 1H, H$_{1'}$), 6.77 (d, *J* = 12.0 Hz, 1H, H$_{2'}$), 5.75 (dd, *J* = 17.1

Hz, $^{1}J_{H\text{-}P}$ = 18.5 Hz, 1H, $H_{5'}$), 5.21 – 5.12 (m, 1H, H_1), 4.05 (m, 4H, 2xO$\underline{CH_2}$$CH_3$), 2.21 (dd, J = 14.5, 7.8 Hz, 1H, H_{2A}), 1.99 (s, 3H, CH_3), 2.01 - 19.4 (m, 1H, H_{6A}), 1.90 (s, 3H, CH_3), 1.74 – 1.65 (m, 2H, H_{2B} + H_{6B}), 1.44 (s, 3H, CH_3), 1.37 (s, 3H, CH_3), 1.36 (s, 3H, CH_3), 1.30 (t, J = 7.1 Hz, 6H, 2xO$CH_2$$\underline{CH_3}$) ppm. **^{13}C NMR** (100.62 MHz, CD_3OD): δ 172.0 (s), 157.5 (s), 155.1 (d, $^{2}J_{C\text{-}P}$ = 6.5 Hz), 136.8 (d), 135.0 (s, $^{3}J_{C\text{-}P}$ = 24.2 Hz), 120.3 (d, $^{4}J_{C\text{-}P}$ = 2.3 Hz), 111.7 (d, $^{1}J_{C\text{-}P =}$ 191.8 Hz), 74.3 (s), 69.1 (d), 63.3 (t, $^{2}J_{C\text{-}P}$ = 5.5 Hz, 2x), 45.0 (t), 43.8 (t), 37.2 (s), 32.5 (q), 31.5 (q), 30.9 (q), 21.3 (q), 16.7 (q, $^{3}J_{C\text{-}P}$ = 6.4 Hz, 2x), 11.7 (q) ppm. **HRMS** (ESI^+): Calcd. for $C_{21}H_{35}O_6P$ ($[M+H]^+$),415.2244; found, 415.2239. **IR (NaCl):** ν 3600 - 3100 (br, O-H), 2974 (m, C-H), 2930 (m, C-H), 1732 (s, C=O), 1591 (s, C=C), 1247 (s, P=O), 1022 (s, P-O) cm^{-1}.

Data for diethyl (1*E*,3*E*,5*Z*,2*R*,4'*S*)-5-(-2',4'-dihydroxy-2,6,6-trimethylcyclohexylidene)-3-methylpenta-1,3-dien-1-yl)phosphonate (23). **$[\alpha]_D^{26}$** -145 (c 0.36, MeOH). **^{1}H NMR** (400.13 MHz, CD_3OD): δ 7.17 (dd, J = 17.1 Hz, $^{2}J_{H\text{-}P}$ = 22.2 Hz, 1H, H_2), 6.98 (d, J = 12.0 Hz, 1H, H_5), 6.77 (d, J = 12.0 Hz, 1H, H_4), 5.74 (dd, J = 17.1 Hz, $^{1}J_{H\text{-}P}$ = 18.5 Hz, 1H, H_1), 4.15 - 4.01 (m, 1H, $H_{4'}$), 4.09 - 4.01 (m, 4H, 2xO$\underline{CH_2}$$CH_3$), 2.12 (dd, J = 13.7, 7.0 Hz, 1H, $H_{3'A}$), 1.89 (s, 3H, CH_3), 1.77 (dd, J = 13.2, 5.6 Hz, 1H, $H_{5'A}$), 1.65 - 1.57 (m, 2H, $H_{3'B}$ + $H_{5'B}$), 1.44 (s, 3H, CH_3), 1.36 (s, 3H, CH_3), 1.35 (s, 3H, CH_3), 1.30 (t, J = 7.1 Hz, 6H, 2xO$CH_2$$\underline{CH_3}$) ppm. **^{13}C NMR** (100.62 MHz, CD_3OD): δ 158.0 (s), 155.2 (d, $^{2}J_{C\text{-}P}$ = 6.7 Hz), 137.2 (d), 134.8 (s, $^{3}J_{C\text{-}P}$ = 24.0 Hz), 120.4 (d, $^{4}J_{C\text{-}P}$ = 2.3 Hz), 111.6 (d, $^{1}J_{C\text{-}P}$ = 191.5 Hz), 75.0 (s), 64.3 (d), 63.3 (t, $^{2}J_{C\text{-}P}$ = 5.7 Hz, 2x), 49.8 (t), 47.9 (t), 37.7 (s), 33.1 (q), 32.3 (q), 30.8 (q), 16.7 (q, $^{3}J_{C\text{-}P}$ = 6.5 Hz, 2x), 11.7 (q) ppm. **HRMS** (ESI^+): Calcd. for $C_{19}H_{33}NaO_5P$ ($[M+H]^+$), 395.1958; found, 395.1954. **IR (NaCl):** ν 3600 – 3100 (br, O-H), 2931 (m, C-H), 1589 (m, C=C), 1232 (s, P=O), 1025 (s, P-O) cm^{-1}.

References:

1. Acemoglu, M.; Eugster, C. H., Die diasteromeren Aurochrome: Synthese, Analytik und Chiroptische Eigenschaften. *Helv. Chim. Acta* **1984**, *67*, 471-487.

2. Acemoglu, M.; Eugster, C. H., (5R,6S,5′R,6′S)-5,6,5′,6′-Diepoxy-β,β-cartin: Sythese, Spectroskopische, chiroptische und chromatographische Eigenschaften. *Helv. Chim. Acta* **1984,** *67*, 184-190.

^{1}H-NMR (400.13 MHz, CD_3OD) spectrum of 8.

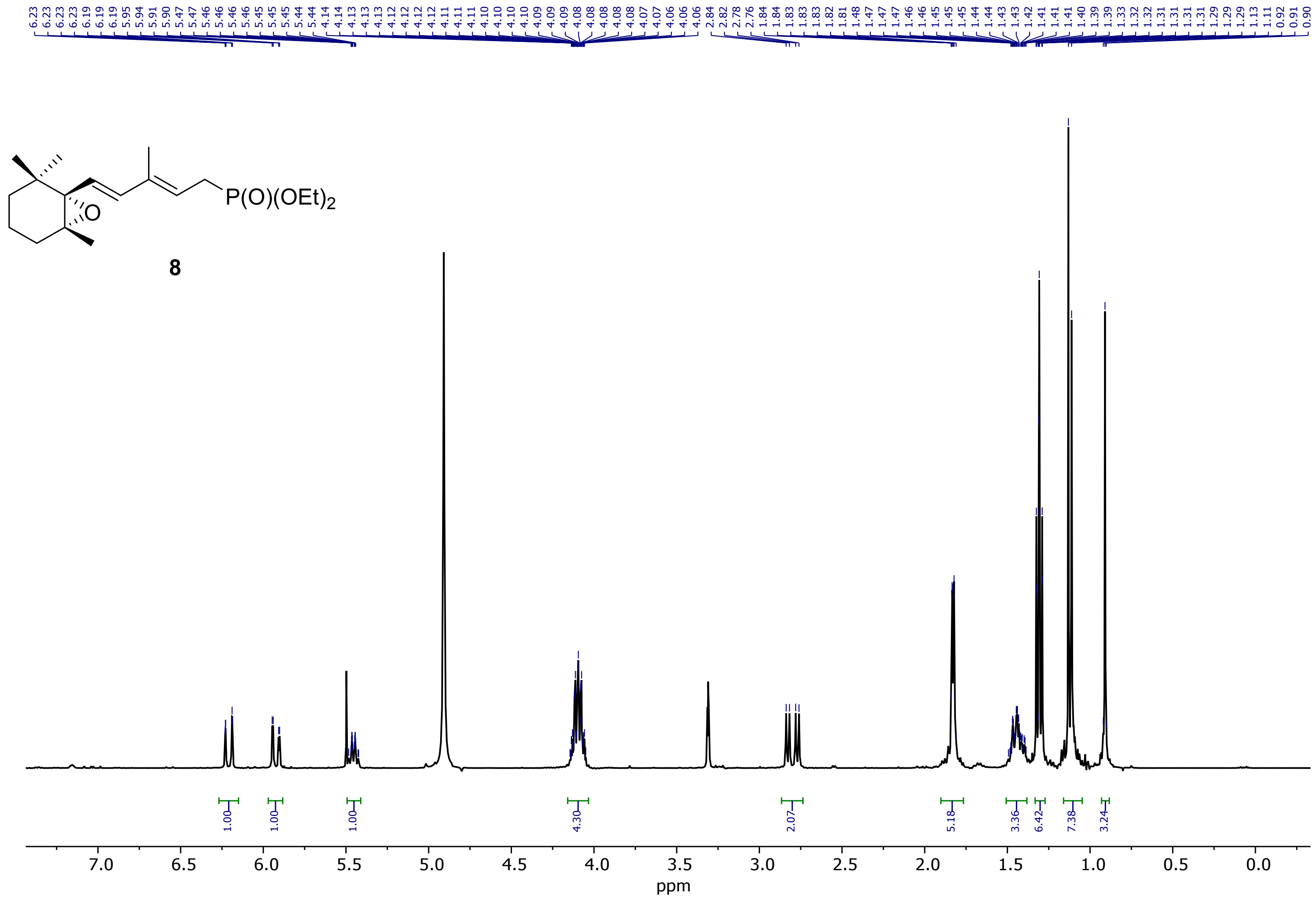

^{13}C-NMR (100.62 MHz, CD_3OD) spectrum of 8.

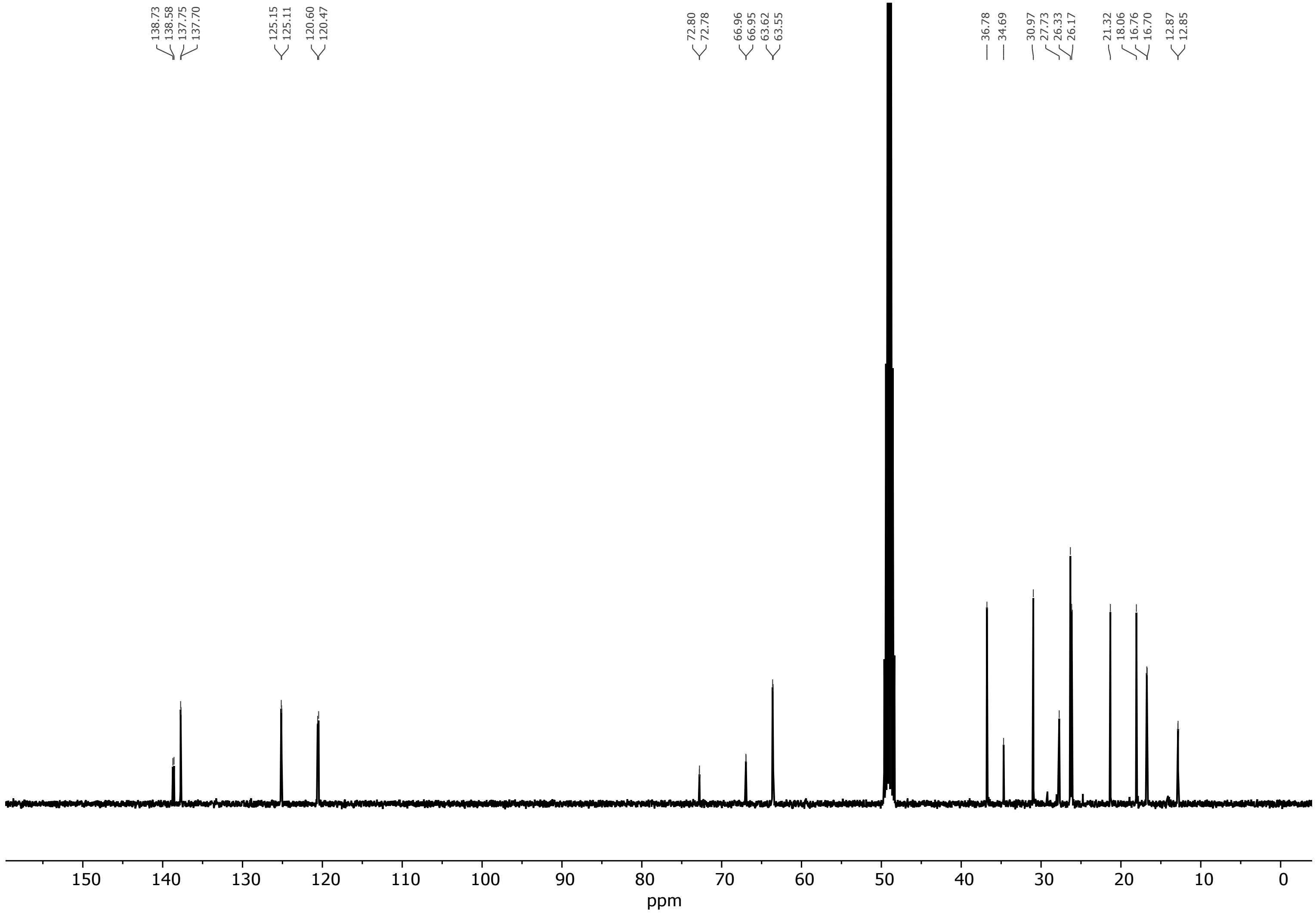

^{1}H-NMR (400.13 MHz, C_6D_6) spectrum of (8*R*,8'*R*)-Aurochrome (4).

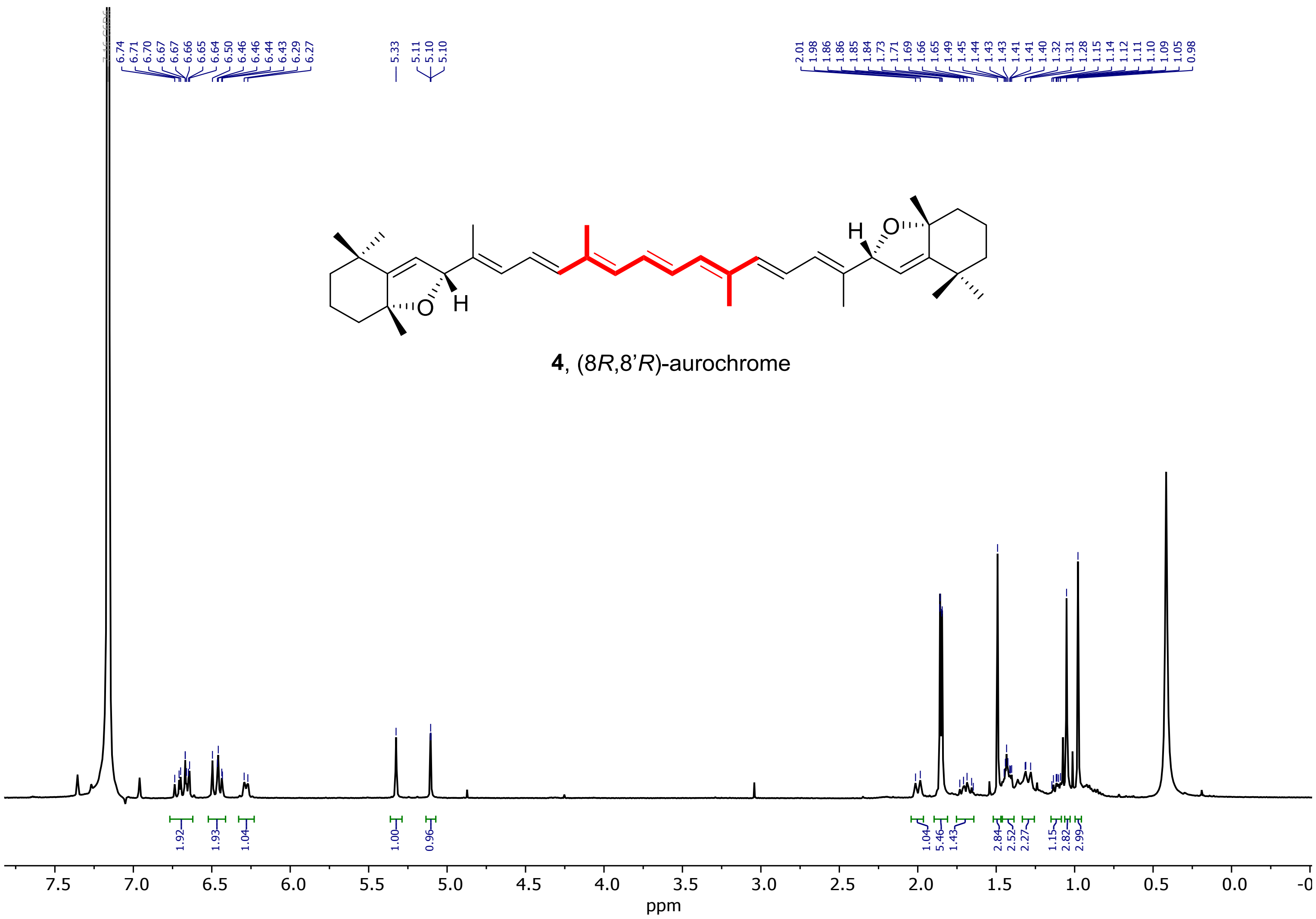

^{13}C-NMR (100.62 MHz, C_6D_6) spectrum of (8*R*,8'*R*)-Aurochrome (4).

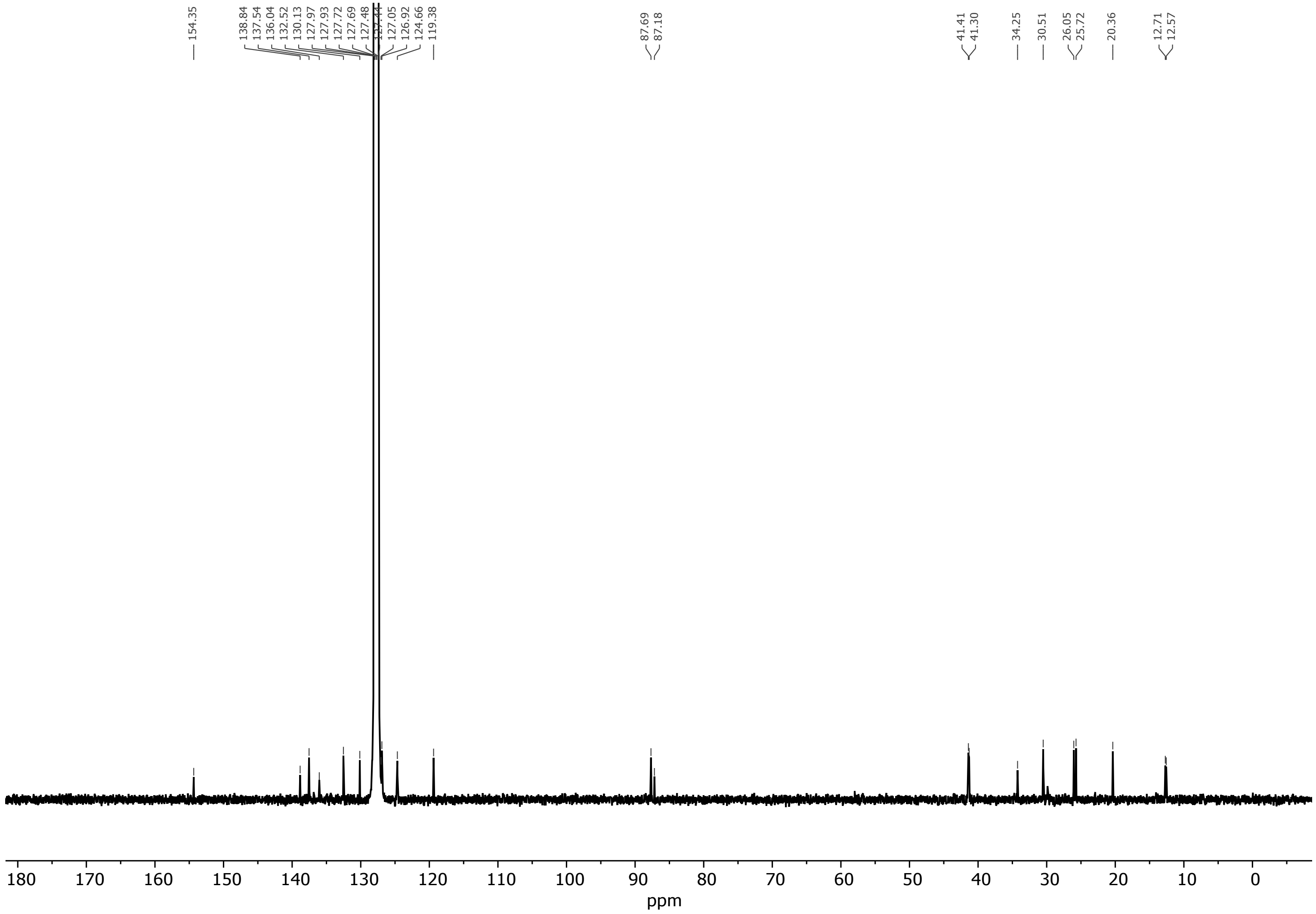

COSY (C_6D_6) spectrum of (8*R*,8’*R*)-Aurochrome (4).

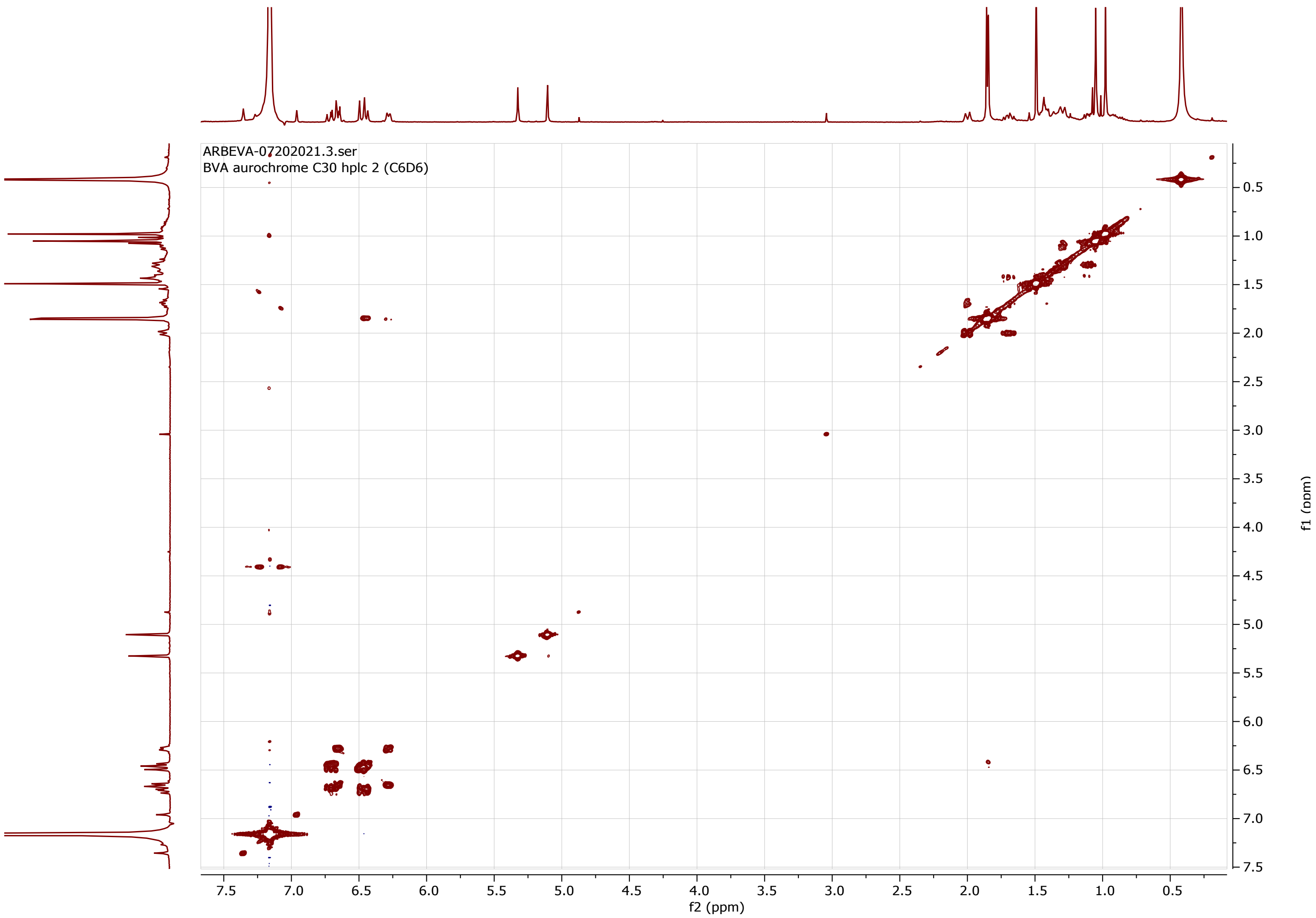

HSQC (C_6D_6) spectrum of (8*R*,8'*R*)-Aurochrome (4).

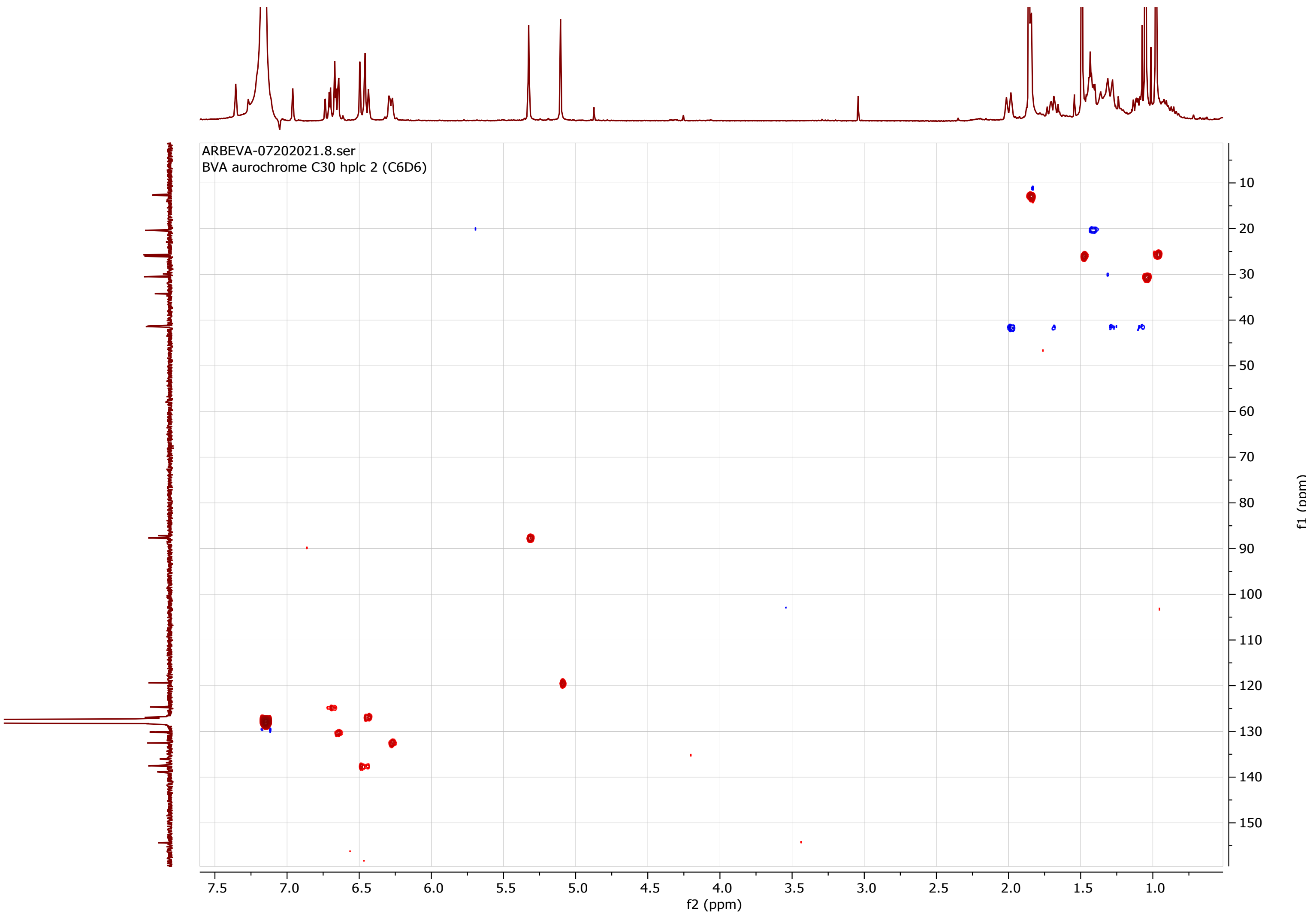

HMBC (C_6D_6) spectrum of (8*R*,8'*R*)-Aurochrome (4).

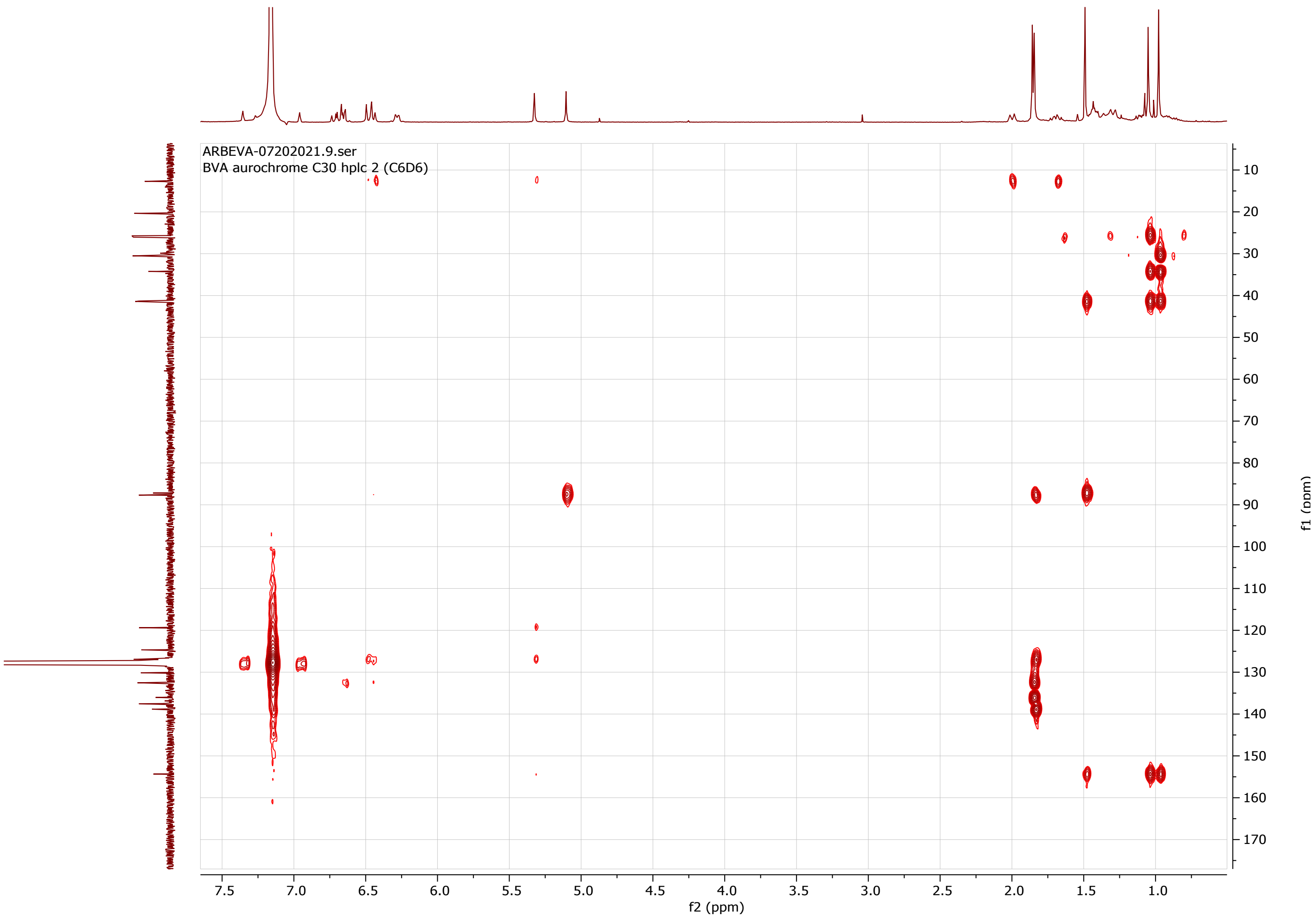

NOESY-1d (400.13 MHz, C_6D_6) of (8*R*,8'*R*)-Aurochrome (4)

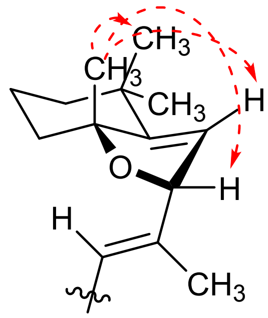

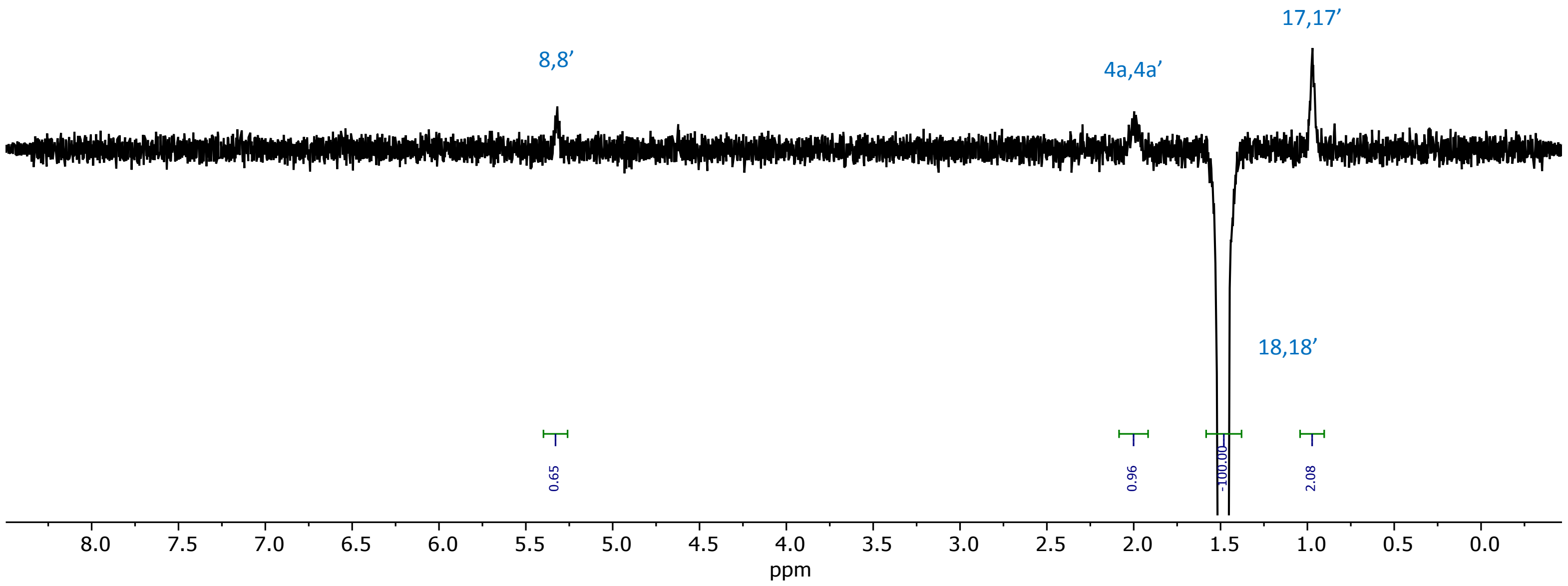

NOESY-1d (400.13 MHz, C_6D_6) of (8*R*,8’*R*)-Aurochrome (4)

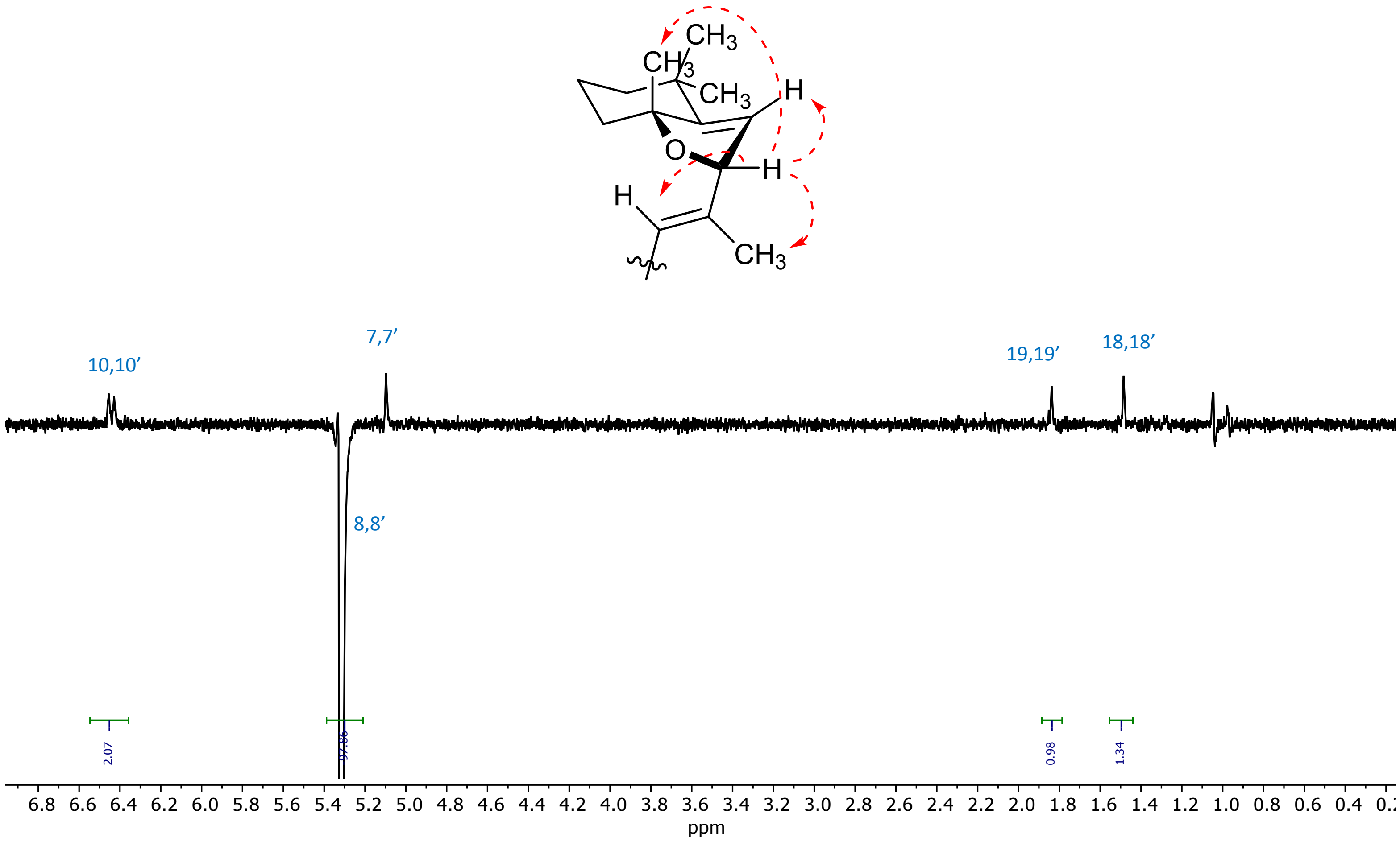

^{1}H-NMR (400.13 MHz, $CDCl_3$) spectrum of (8*R*,8'*R*)-Aurochrome (4).

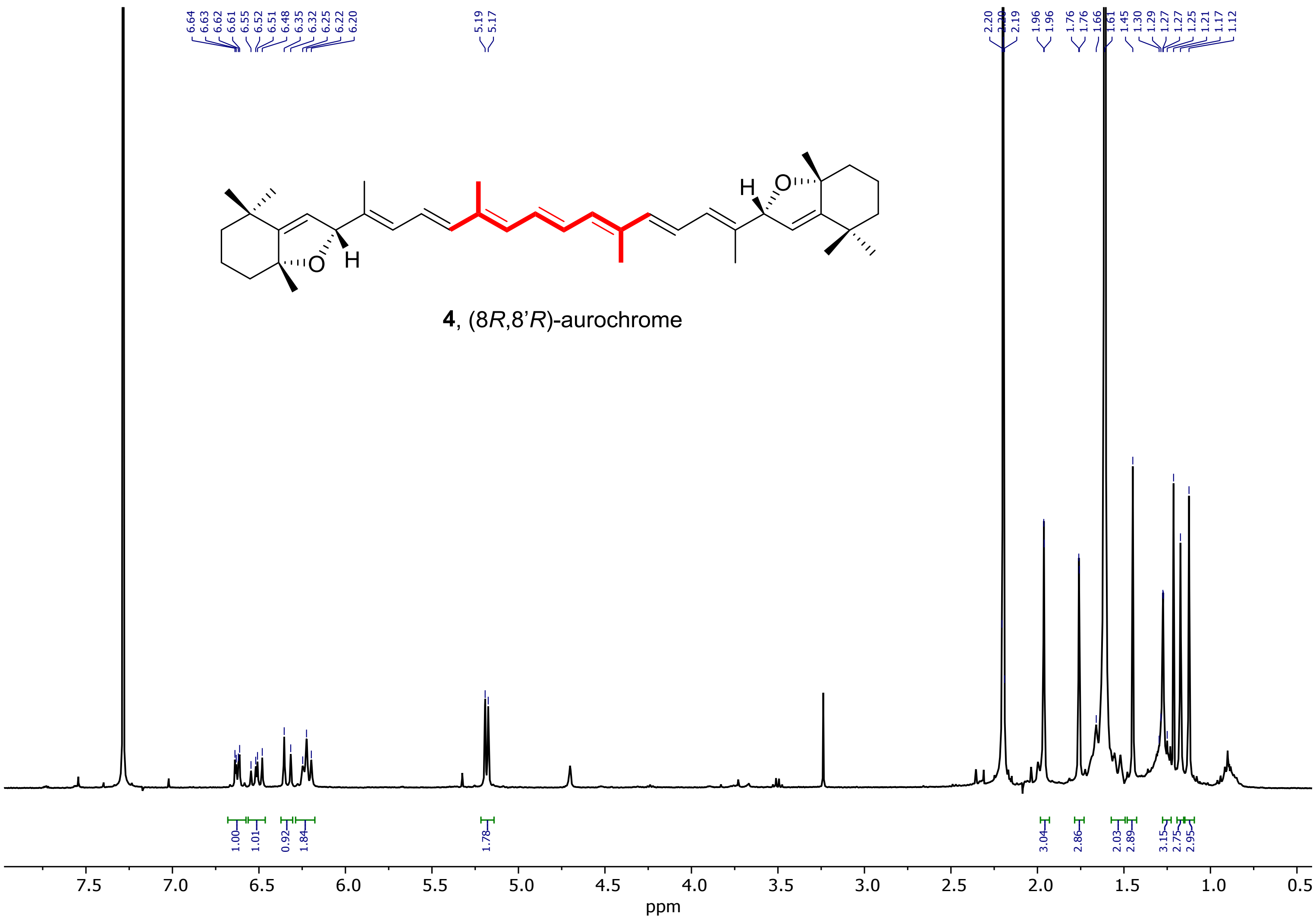

^{1}H-NMR (400.13 MHz, CD_3OD) spectrum of 9

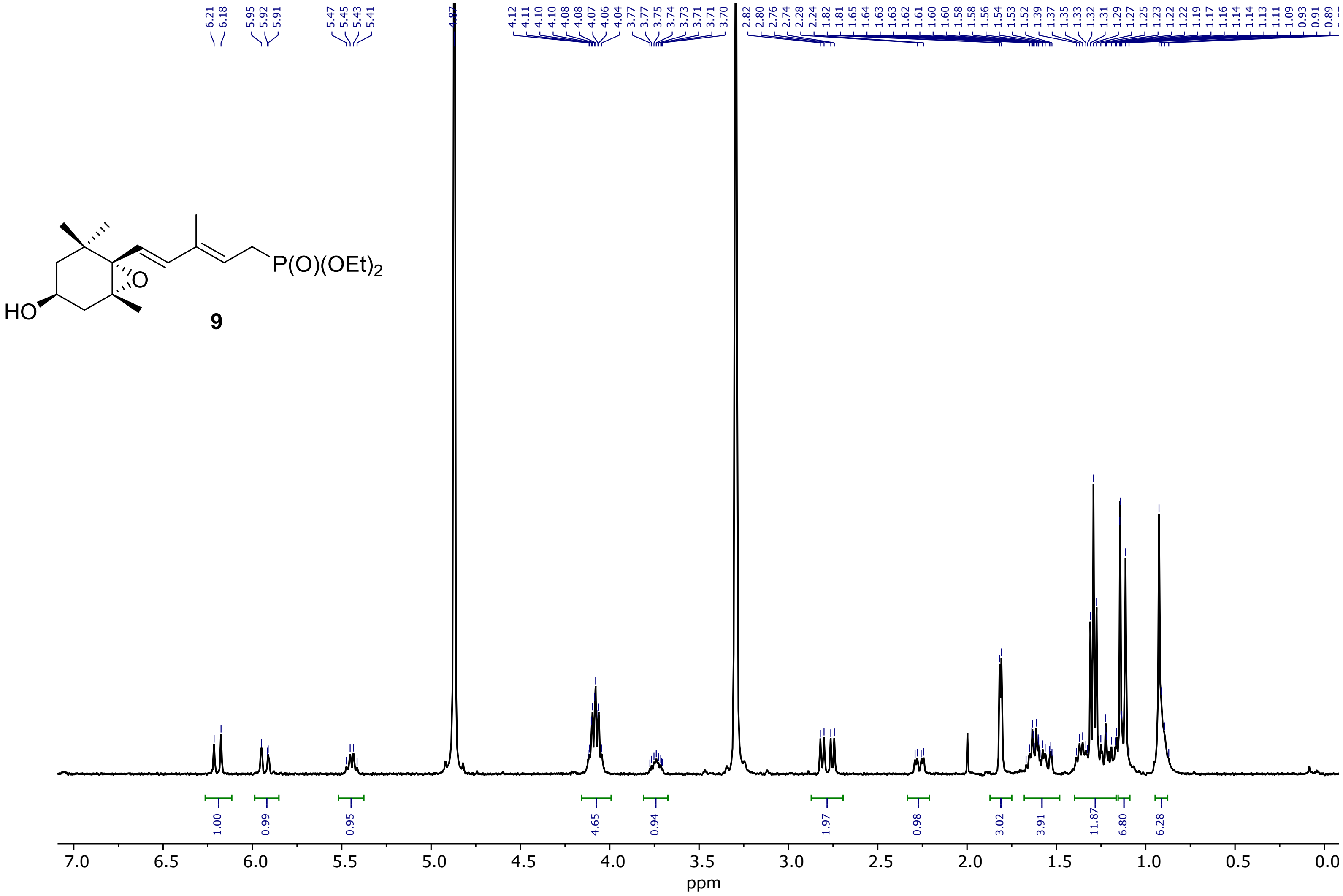

^{13}C-NMR (100.62 MHz, CD_3OD) spectrum of 9

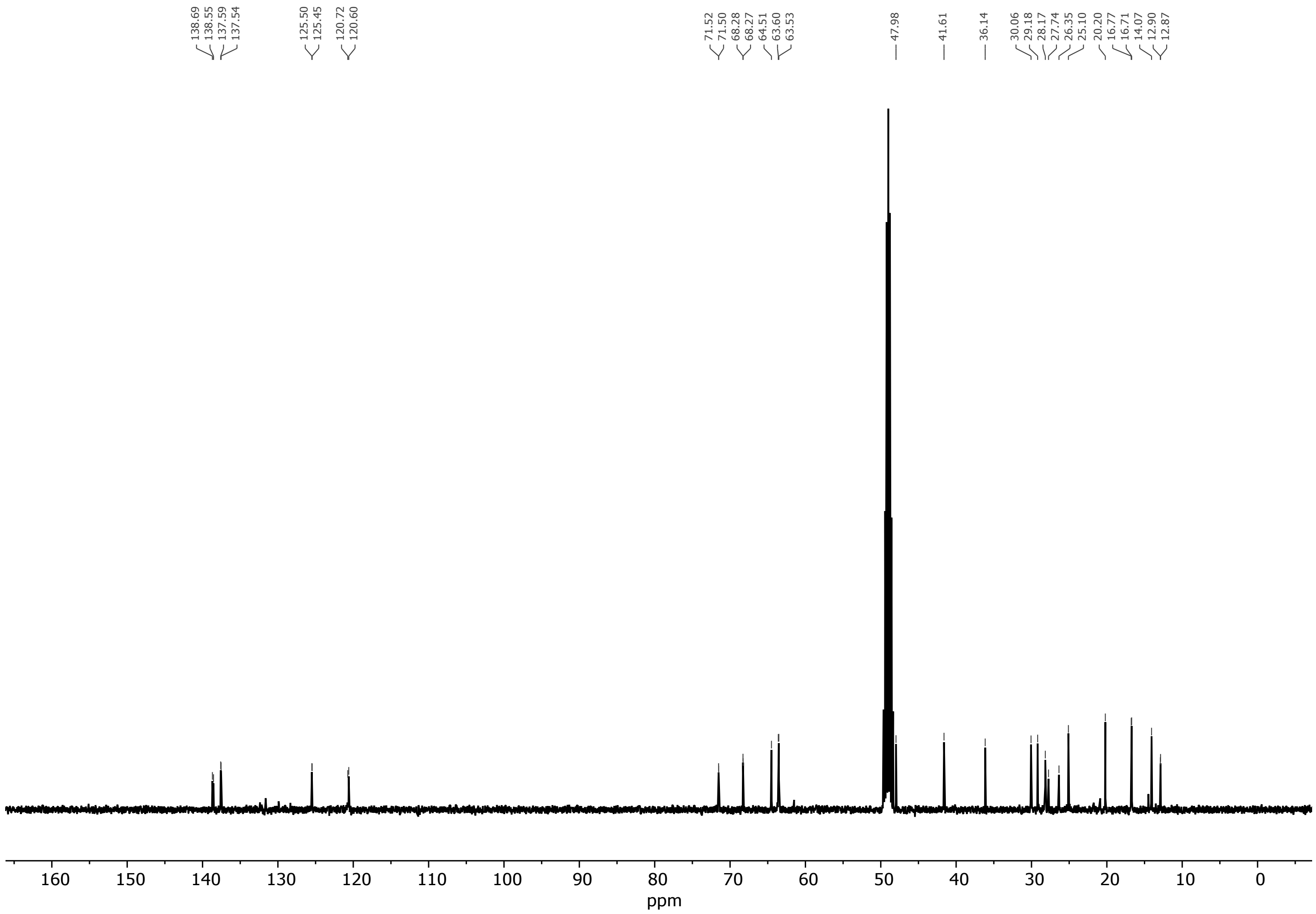

^{1}H-NMR (400.13 MHz, CD_3OD) spectrum of **10**.

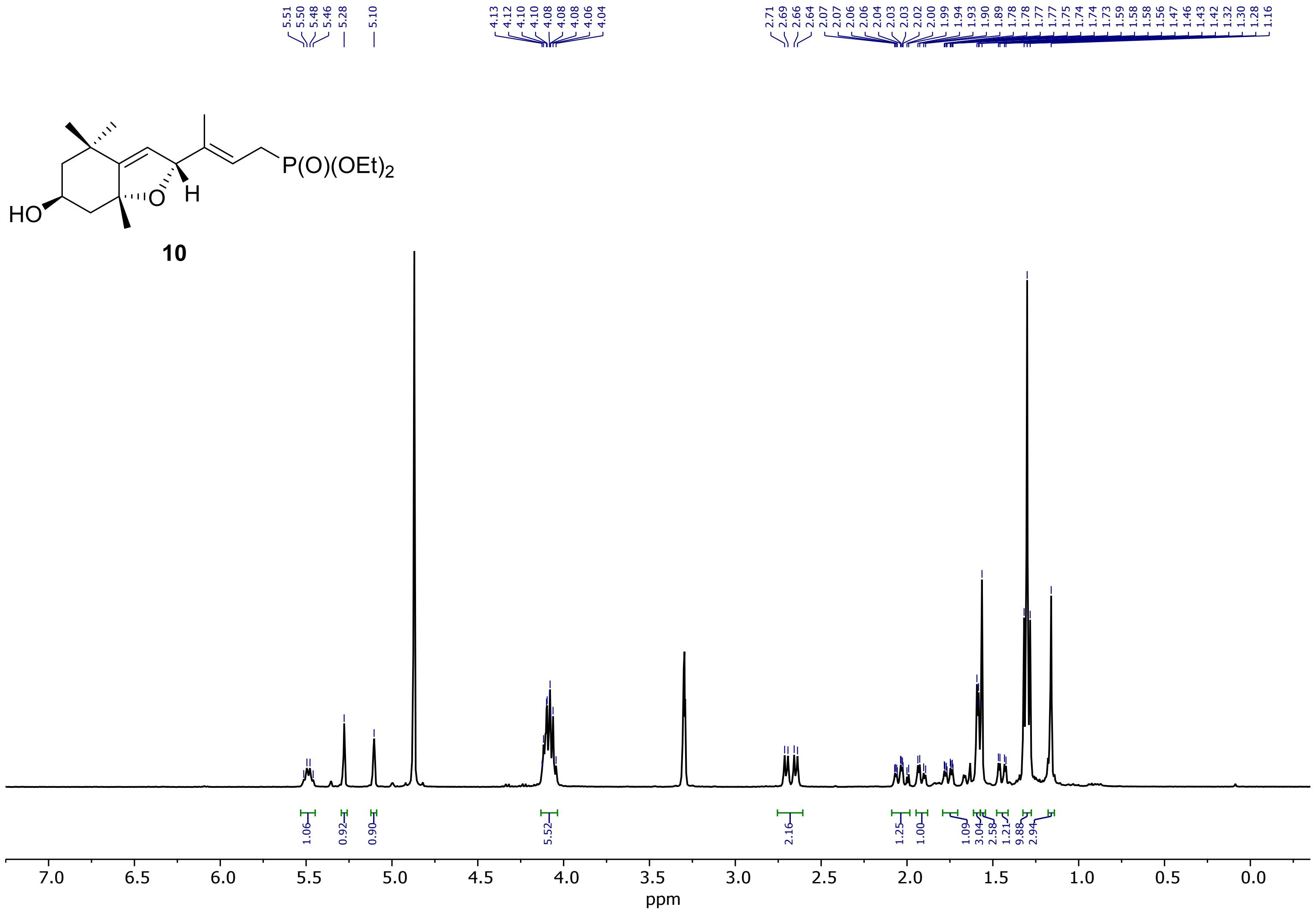

^{13}C-NMR (100.62 MHz, CD_3OD) spectrum of 10

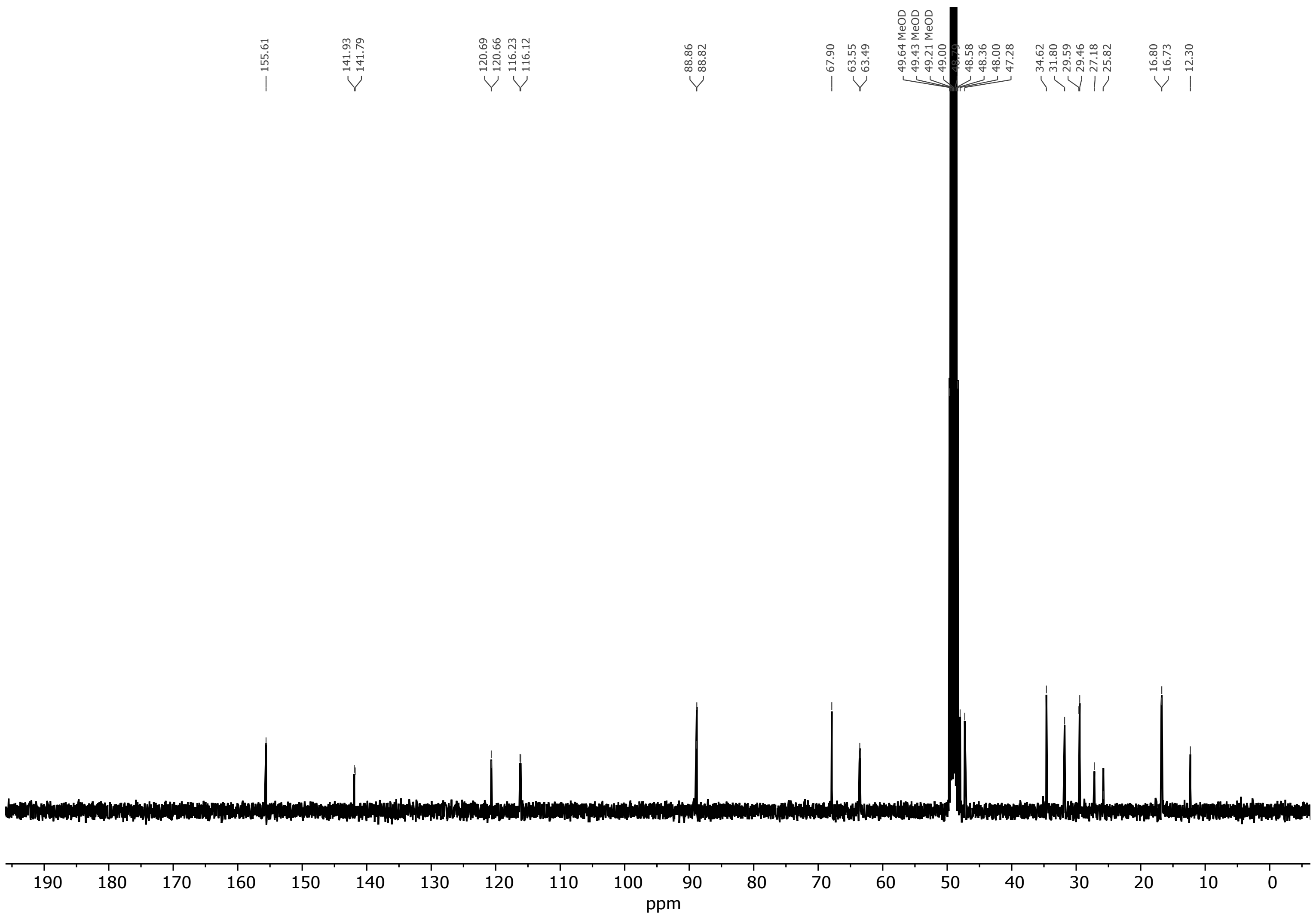

^{1}H-NMR (400.13 MHz, CD_3OD) spectrum of **21**.

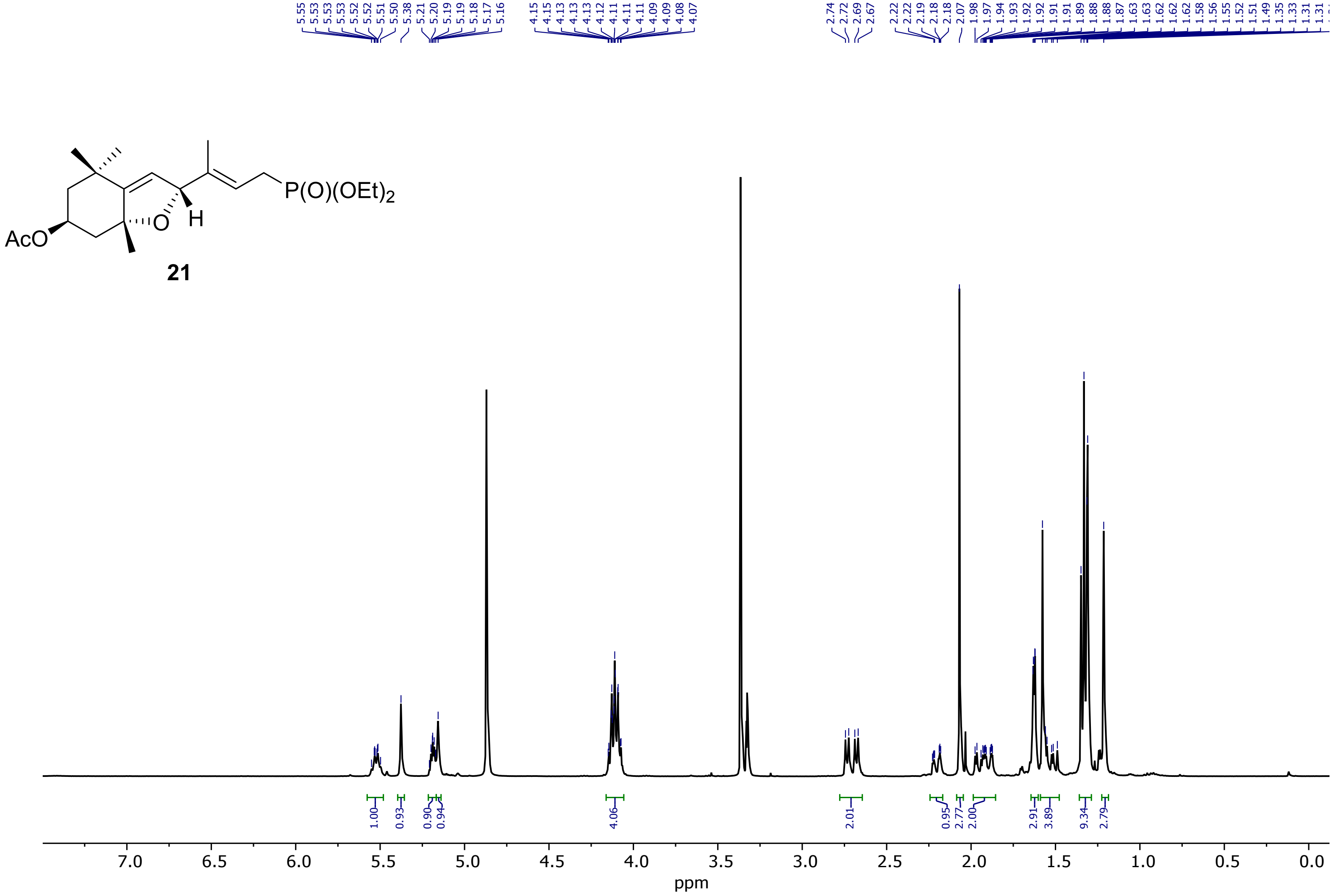

^{13}C-NMR (100.62 MHz, CD_3OD) spectrum of 21

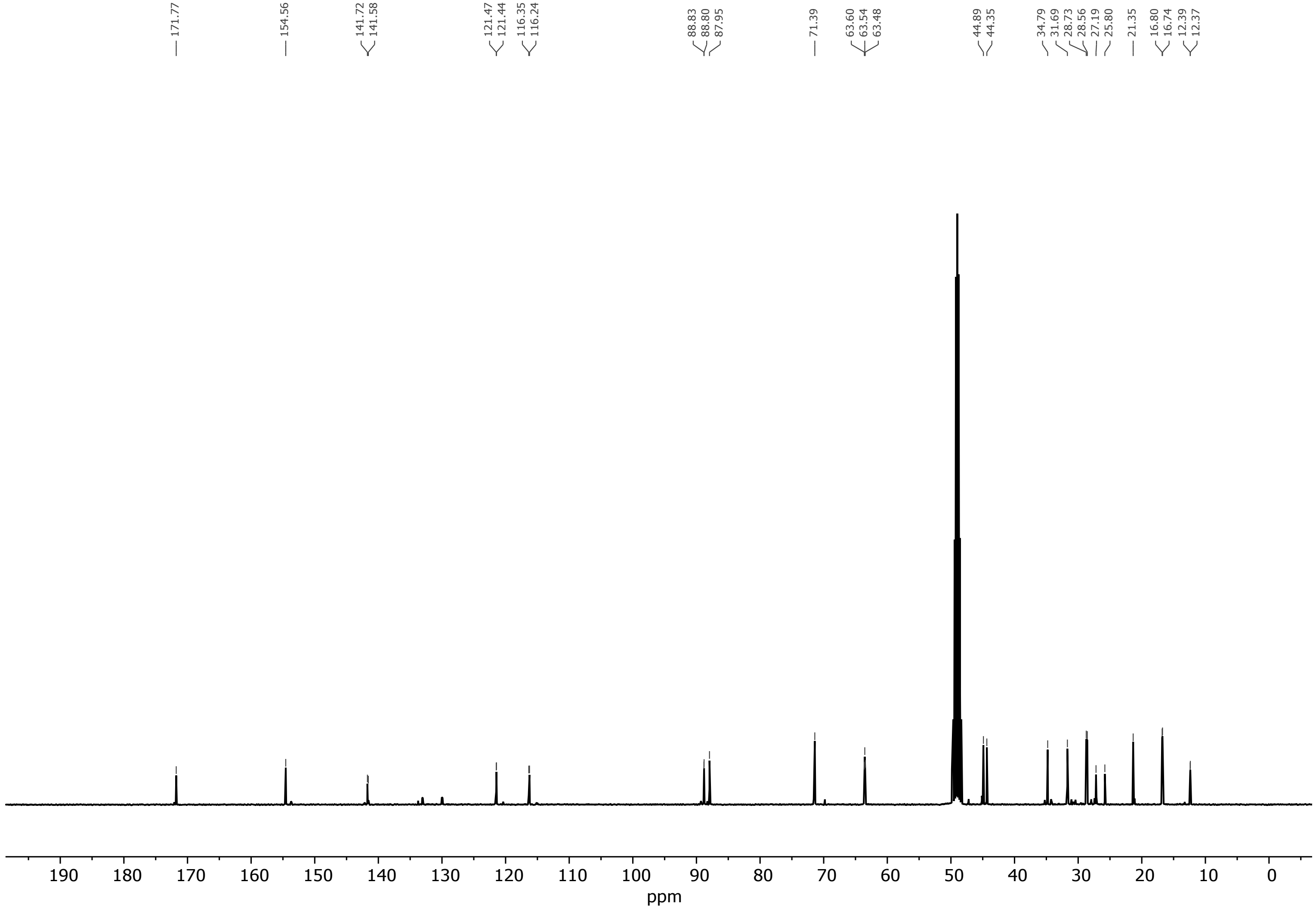

^{1}H-NMR (400.13 MHz, CD_3OD) spectrum of 20.

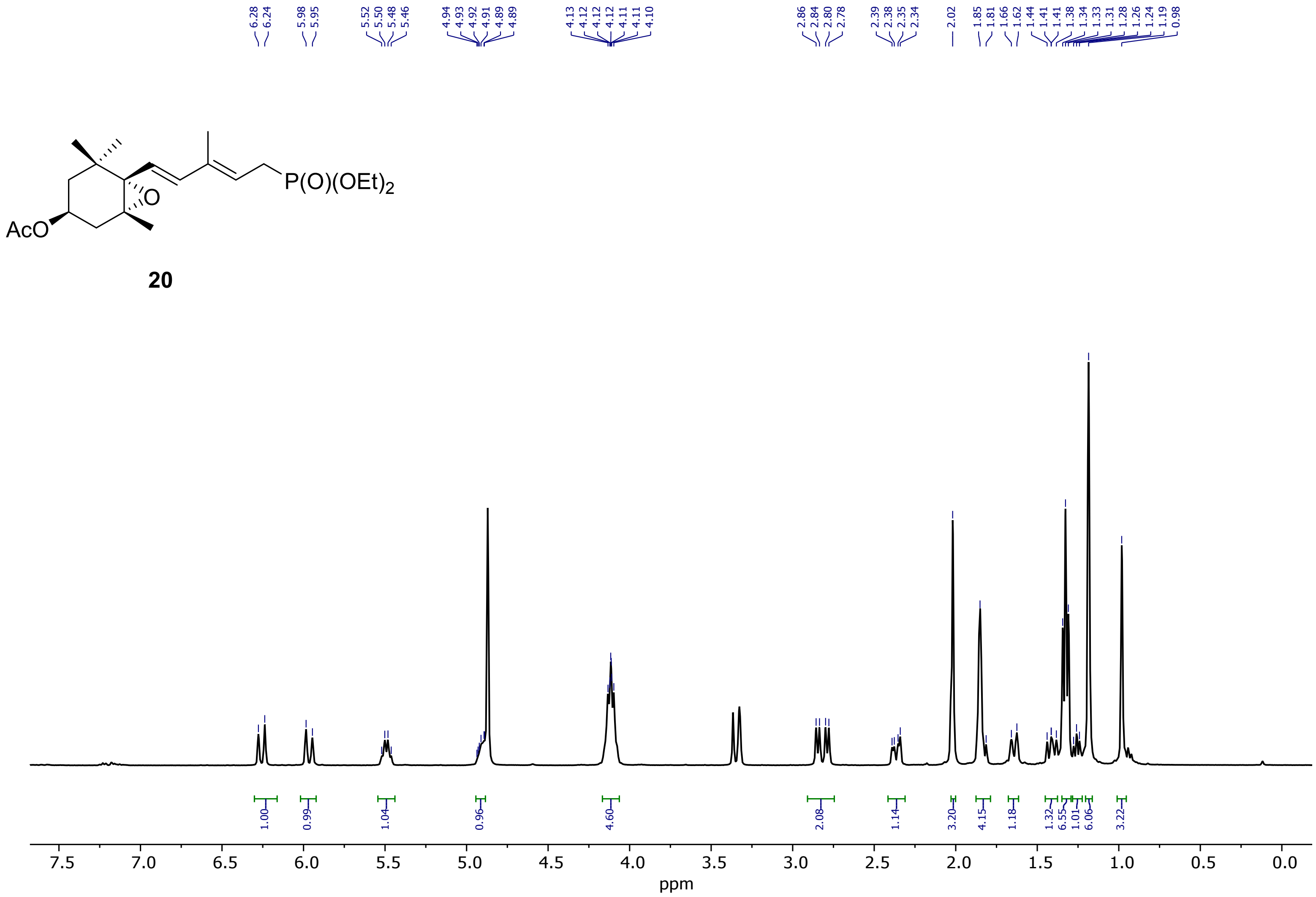

^{13}C-NMR (100.62 MHz, CD_3OD) spectrum of 20

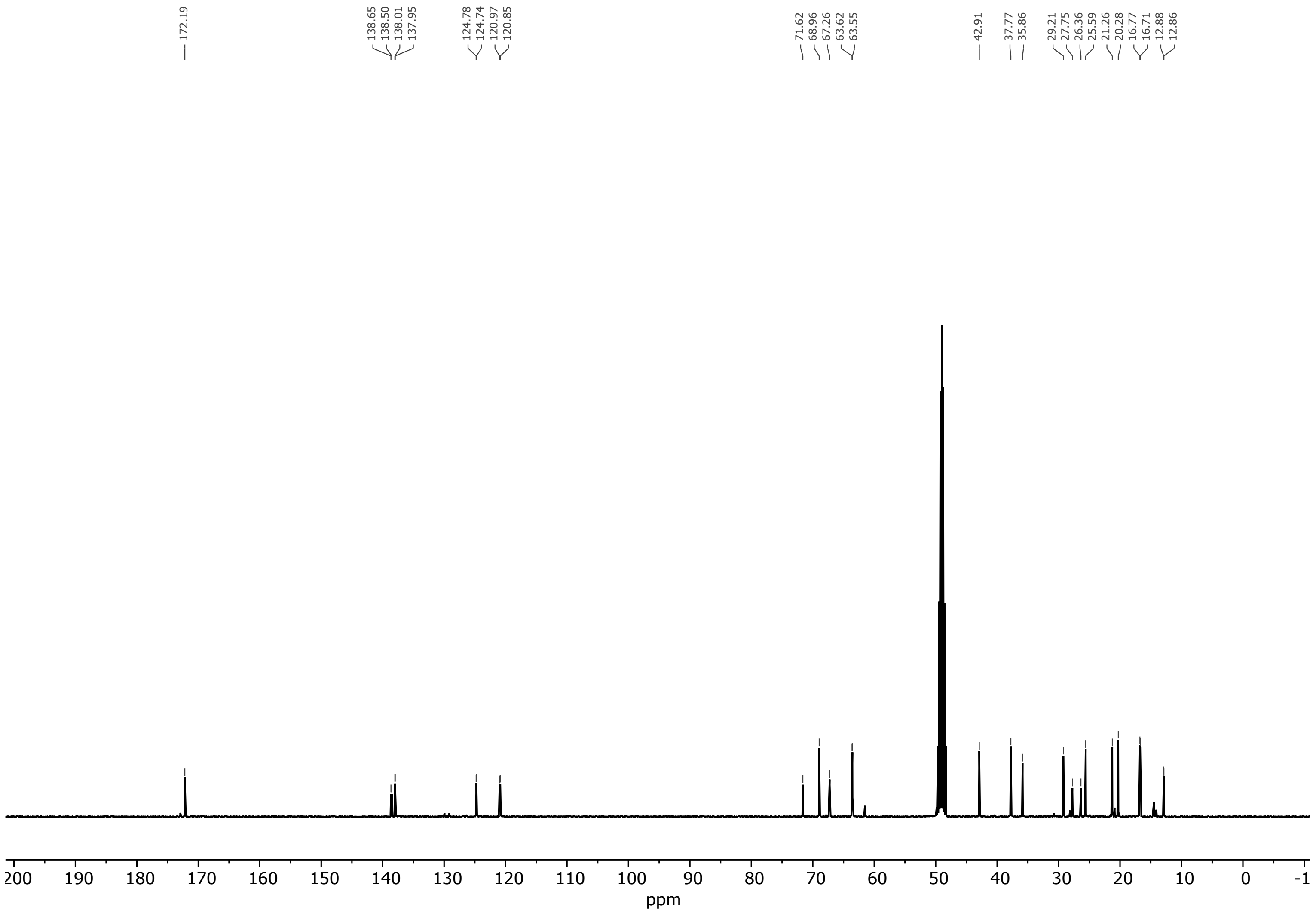

^{1}H-NMR (400.13 MHz, CD_3OD) spectrum of **22**.

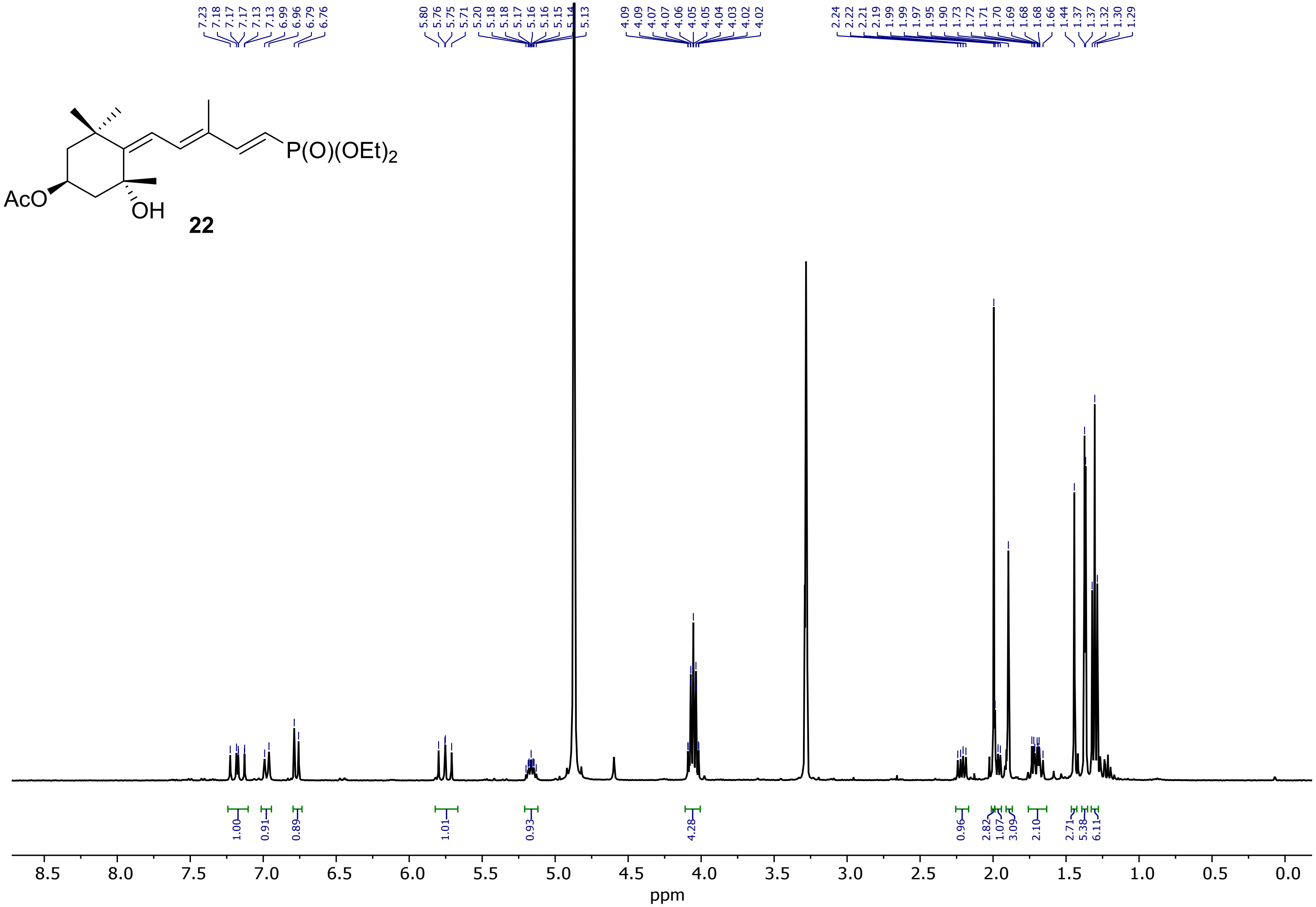

^{13}C-NMR (100.62 MHz, CD_3OD) spectrum of 22.

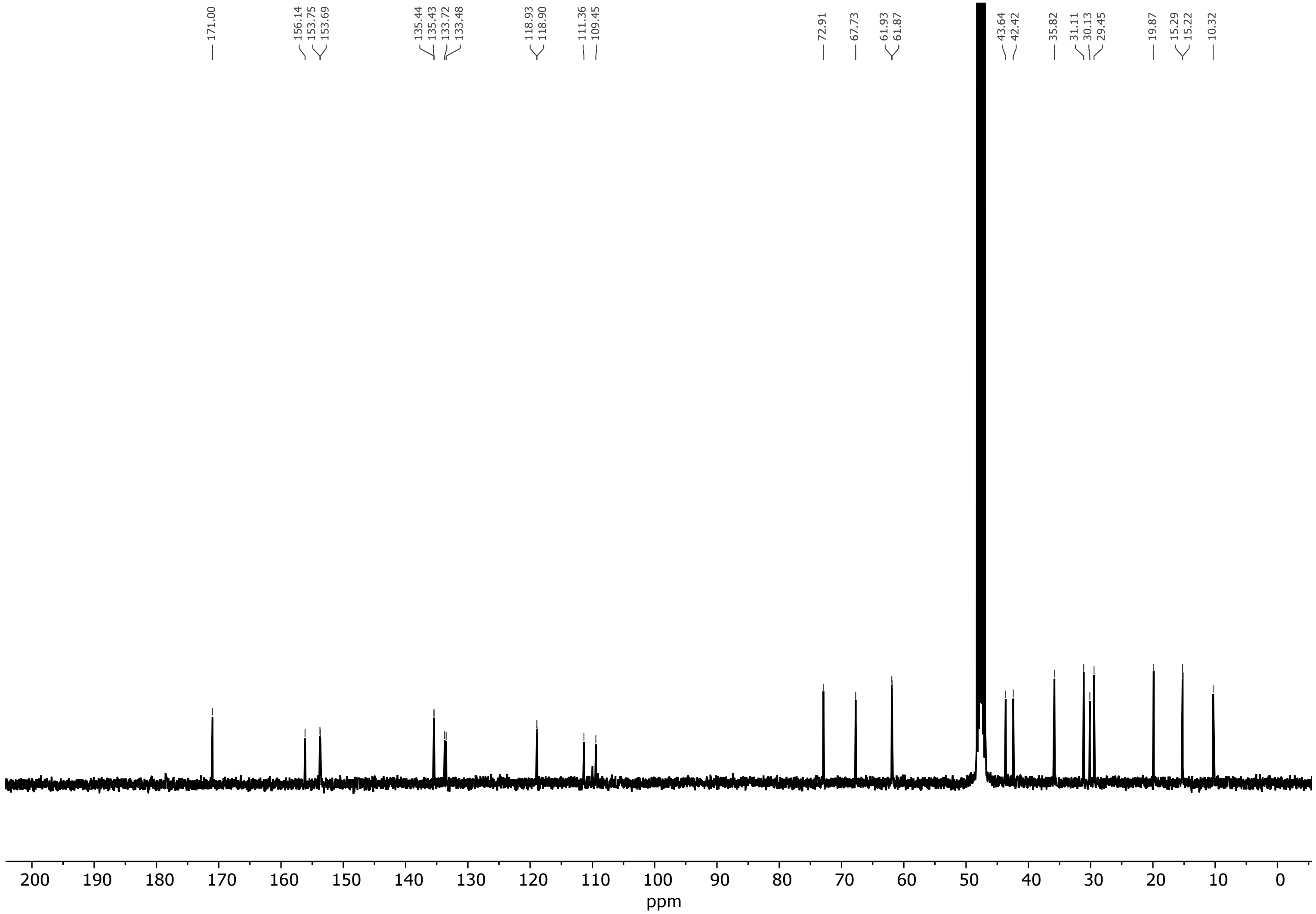

^{1}H-NMR (400.13 MHz, CD_3OD) spectrum of 23.

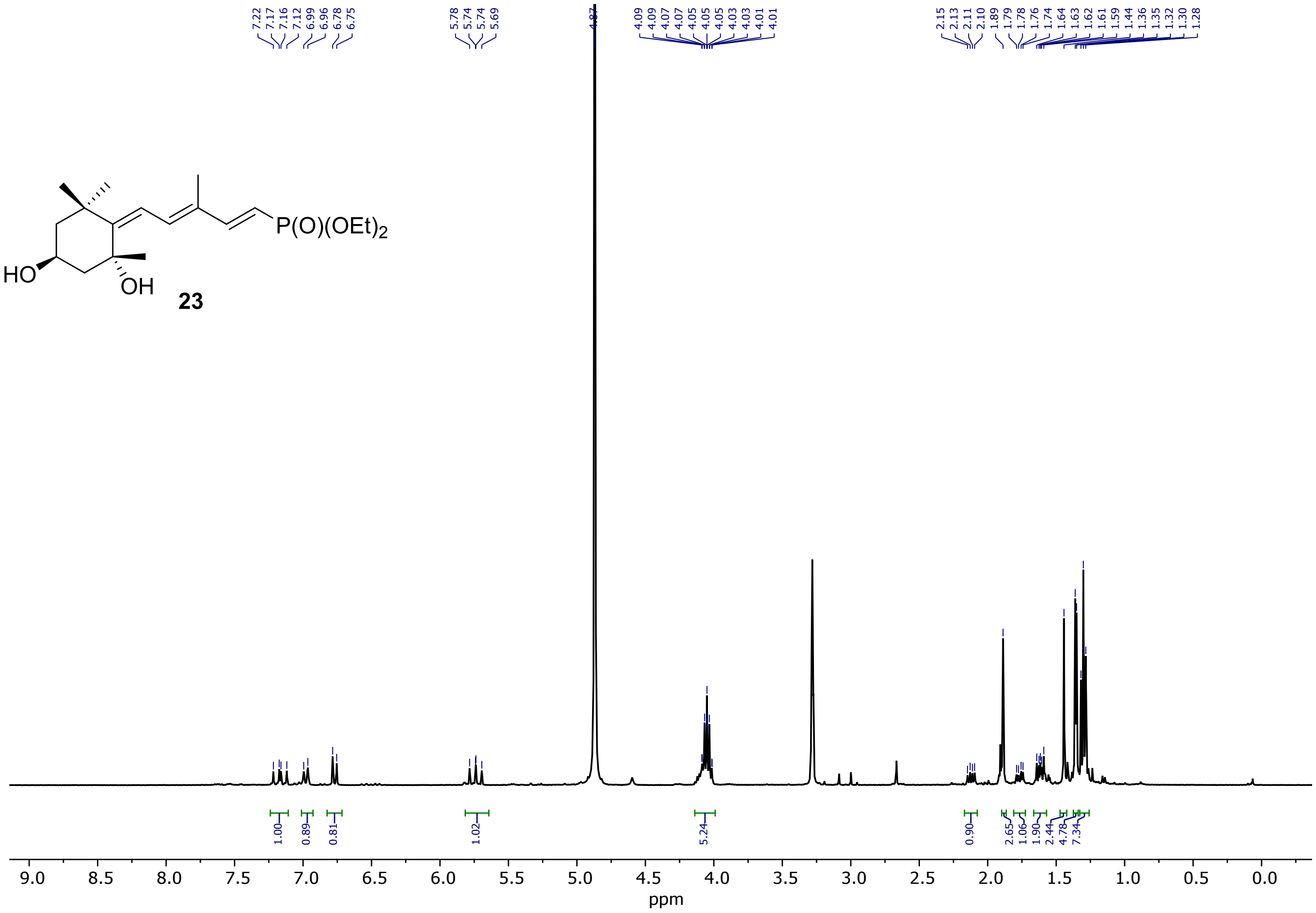

^{13}C-NMR (100.62 MHz, CD_3OD) spectrum of 23.

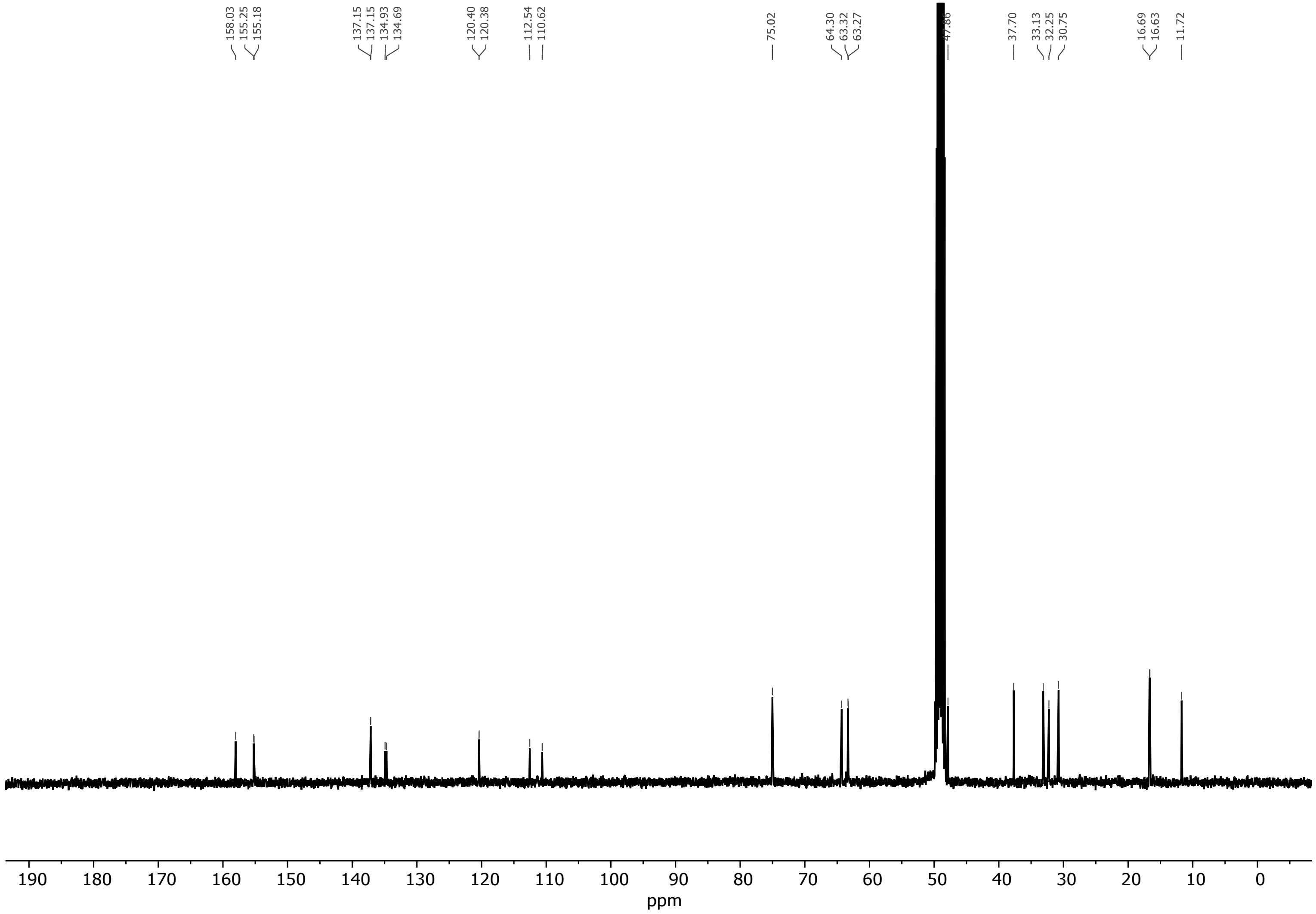

^{1}H-NMR (400.13 MHz, C_6D_6) spectrum of auroxanthin (6).

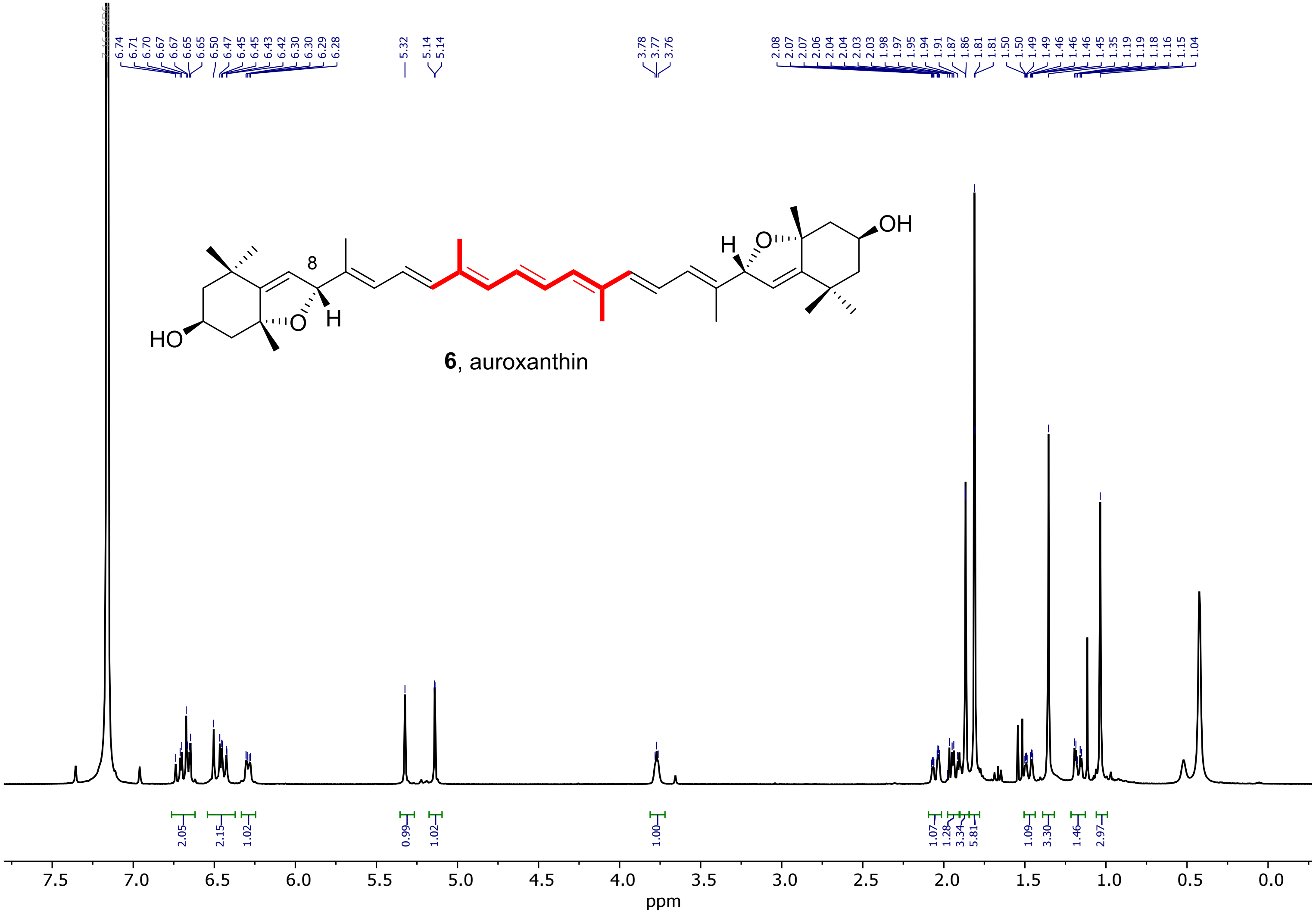

^{13}C-NMR (100.64 MHz, C_6D_6) spectrum of auroxanthin (6).

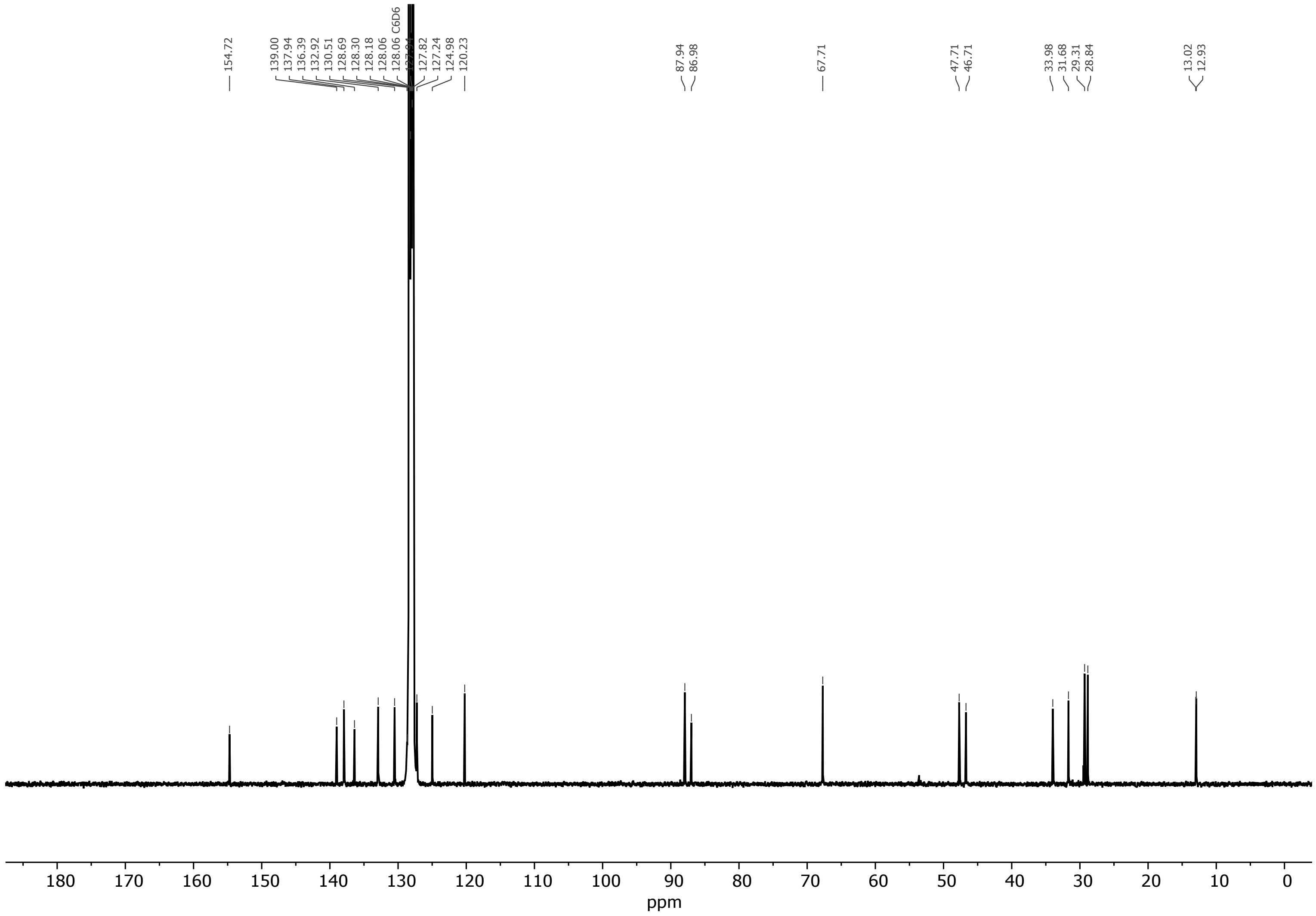

COSY (100.64 MHz, C_6D_6) spectrum of auroxanthin (6).

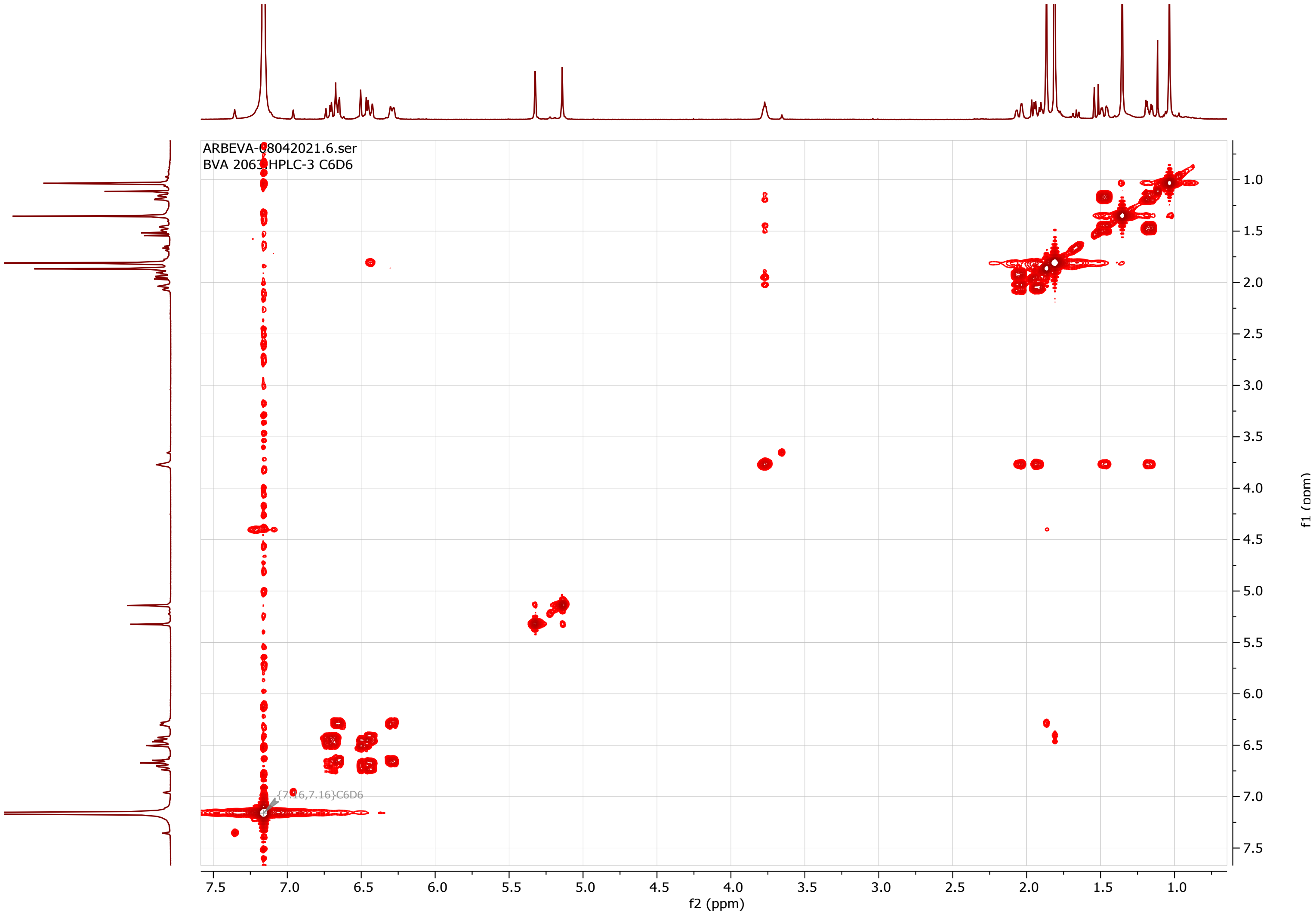

HSQC (C_6D_6) spectrum of auroxanthin (6).

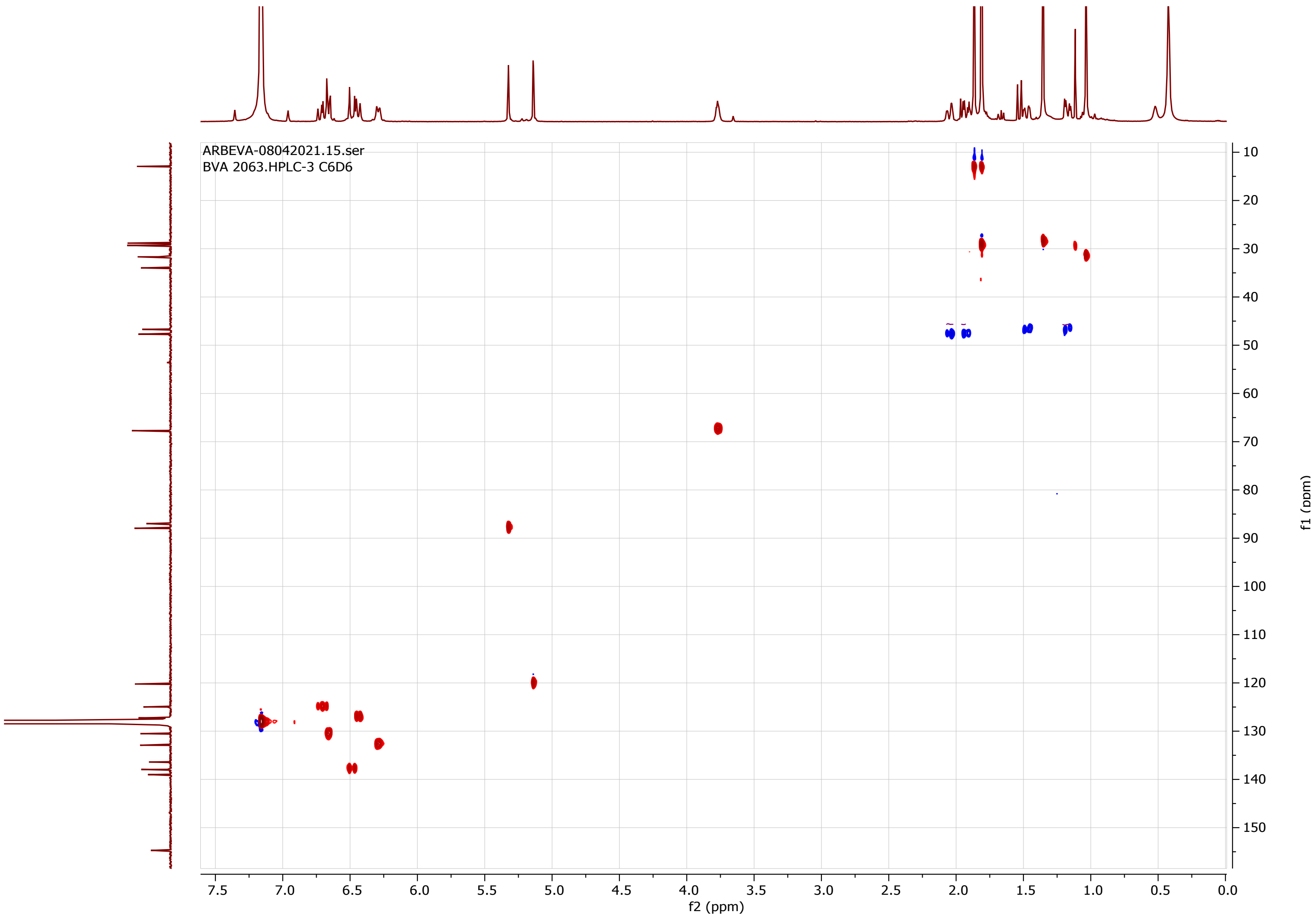

HMBC (C_6D_6) spectrum of auroxanthin (6).

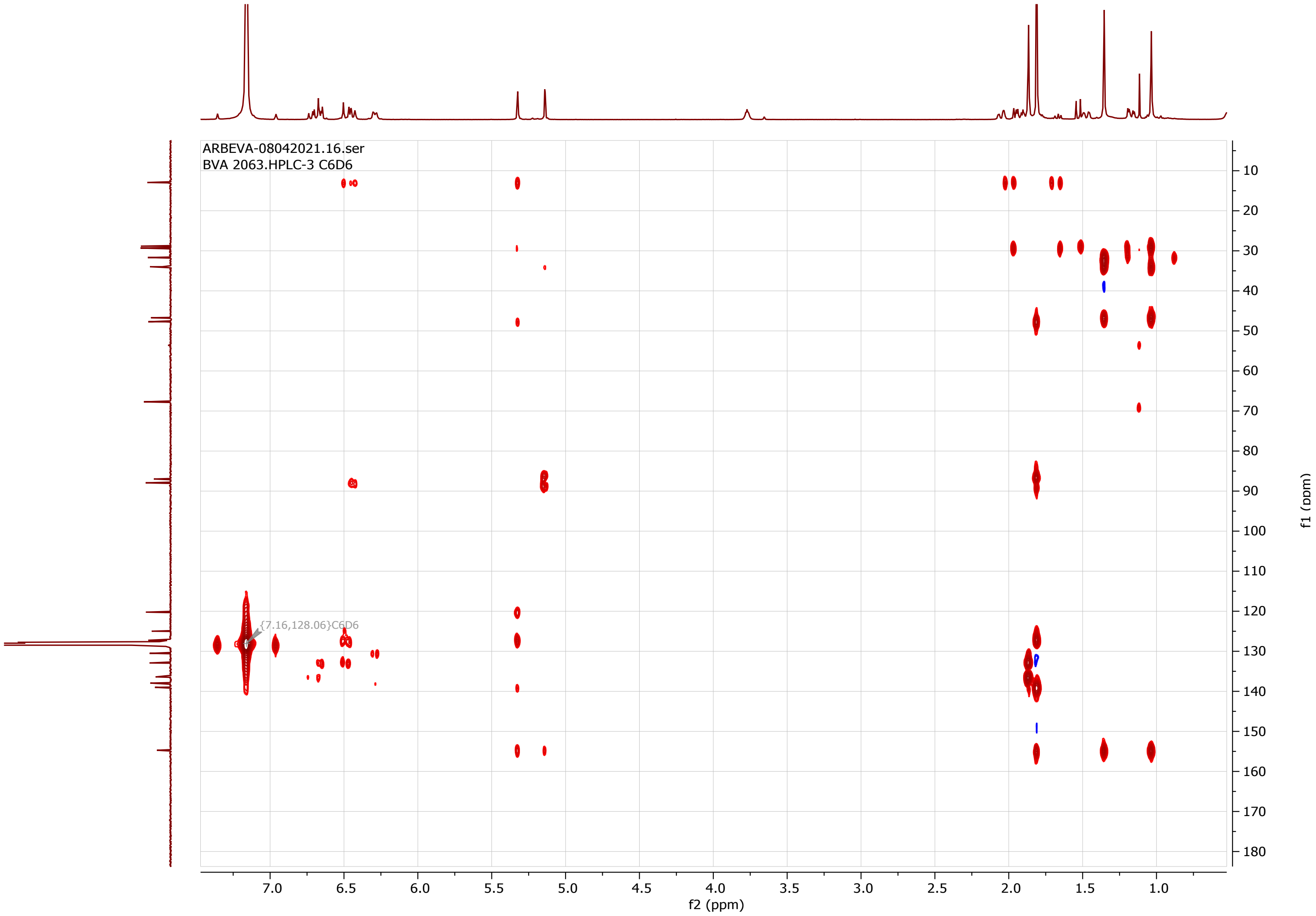

NOESY-1d (C_6D_6) of auroxanthin (6).

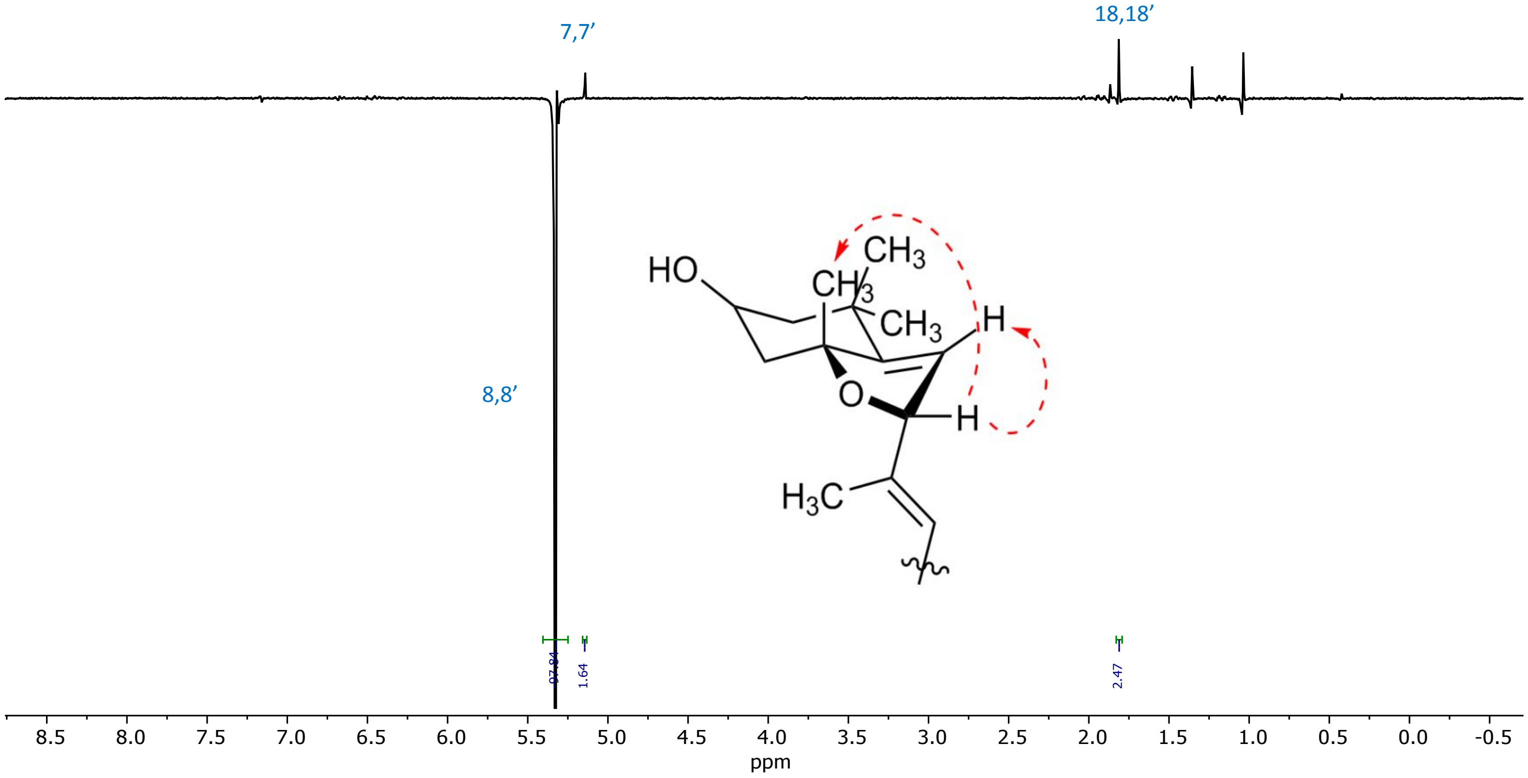

NOESY-1d (C_6D_6) of auroxanthin (6).

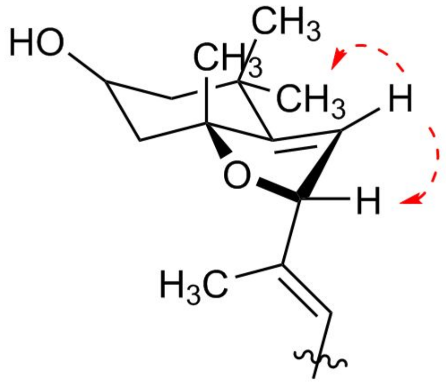

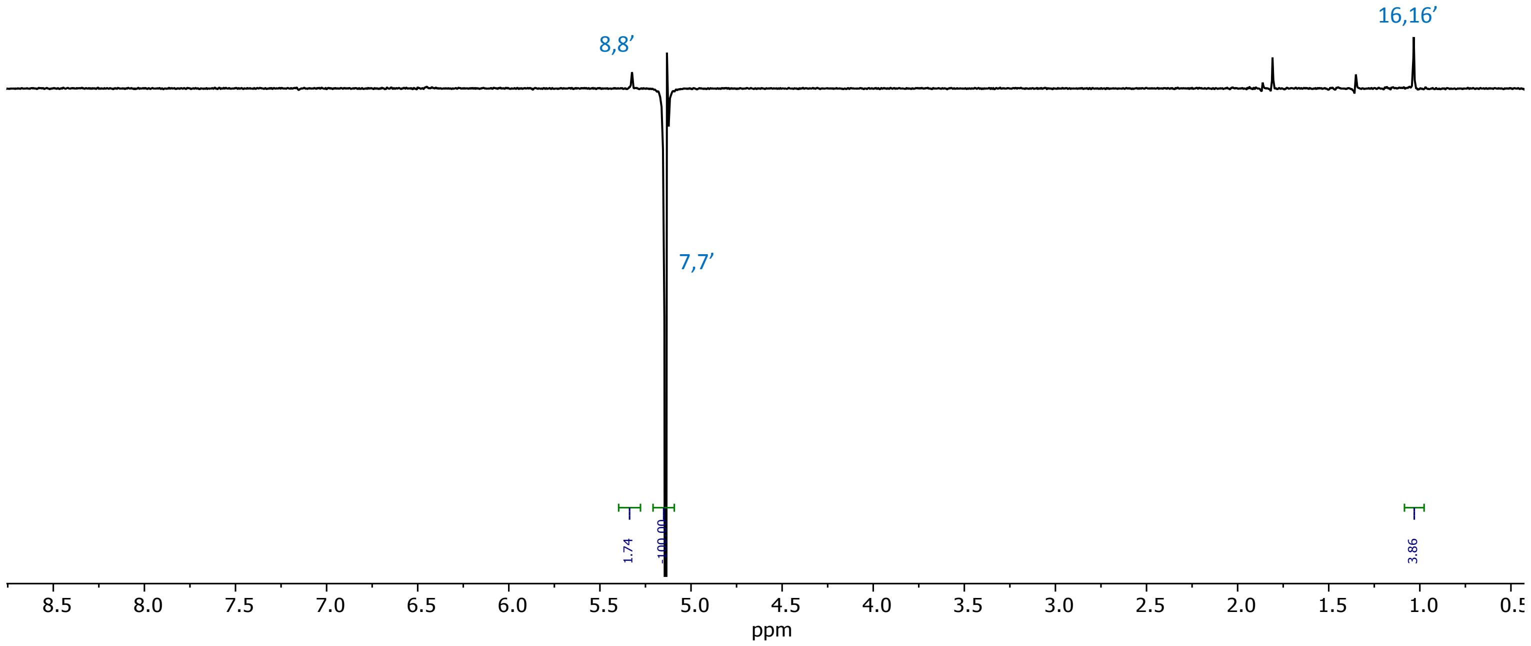

NOESY-1d (C_6D_6) of auroxanthin (6).

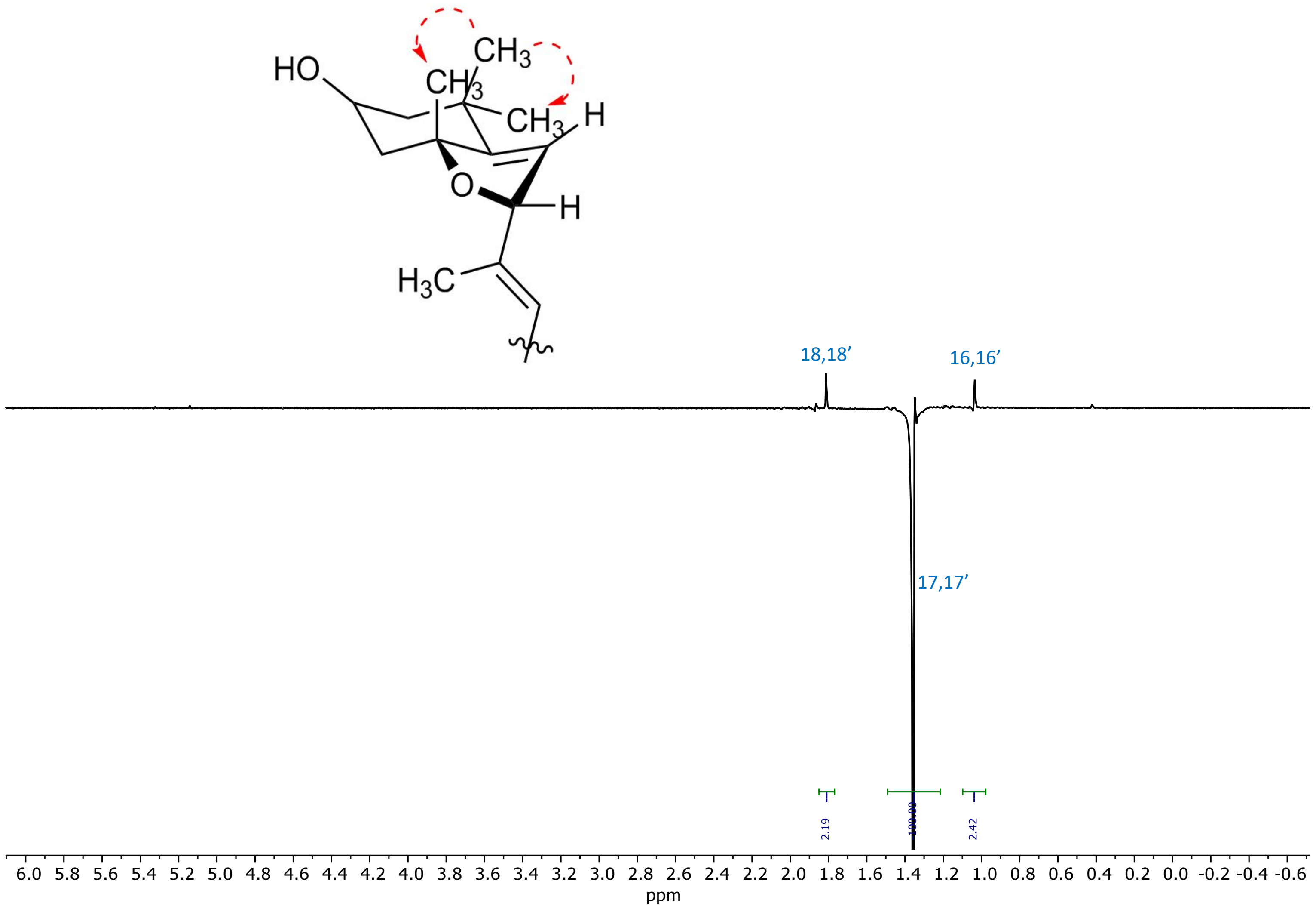

^{1}H-NMR (400.13 MHz, $CDCl_3$) spectrum of auroxanthin (6)

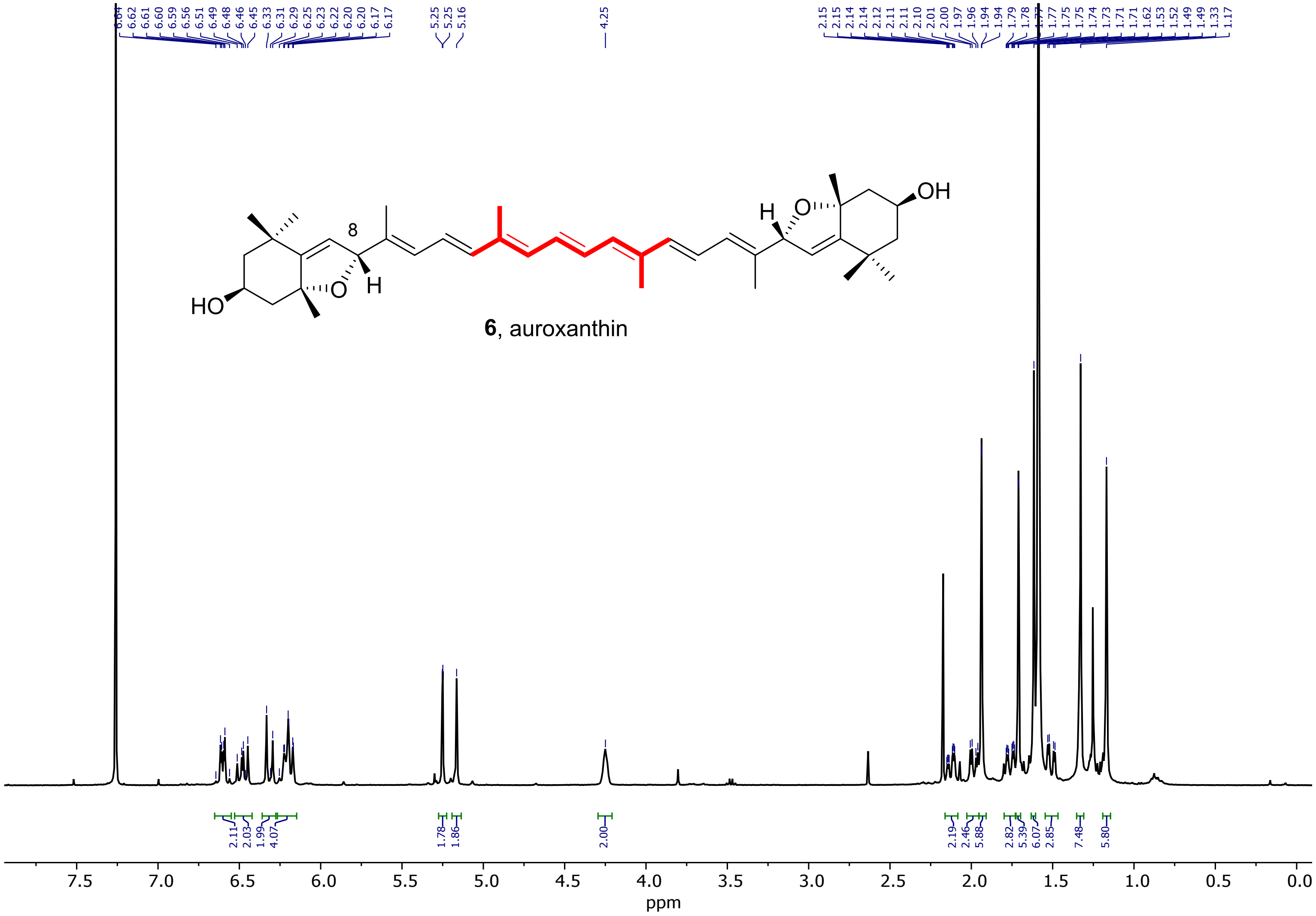

^{1}H-NMR (400.13 MHz, C_6D_6) spectrum of 15.

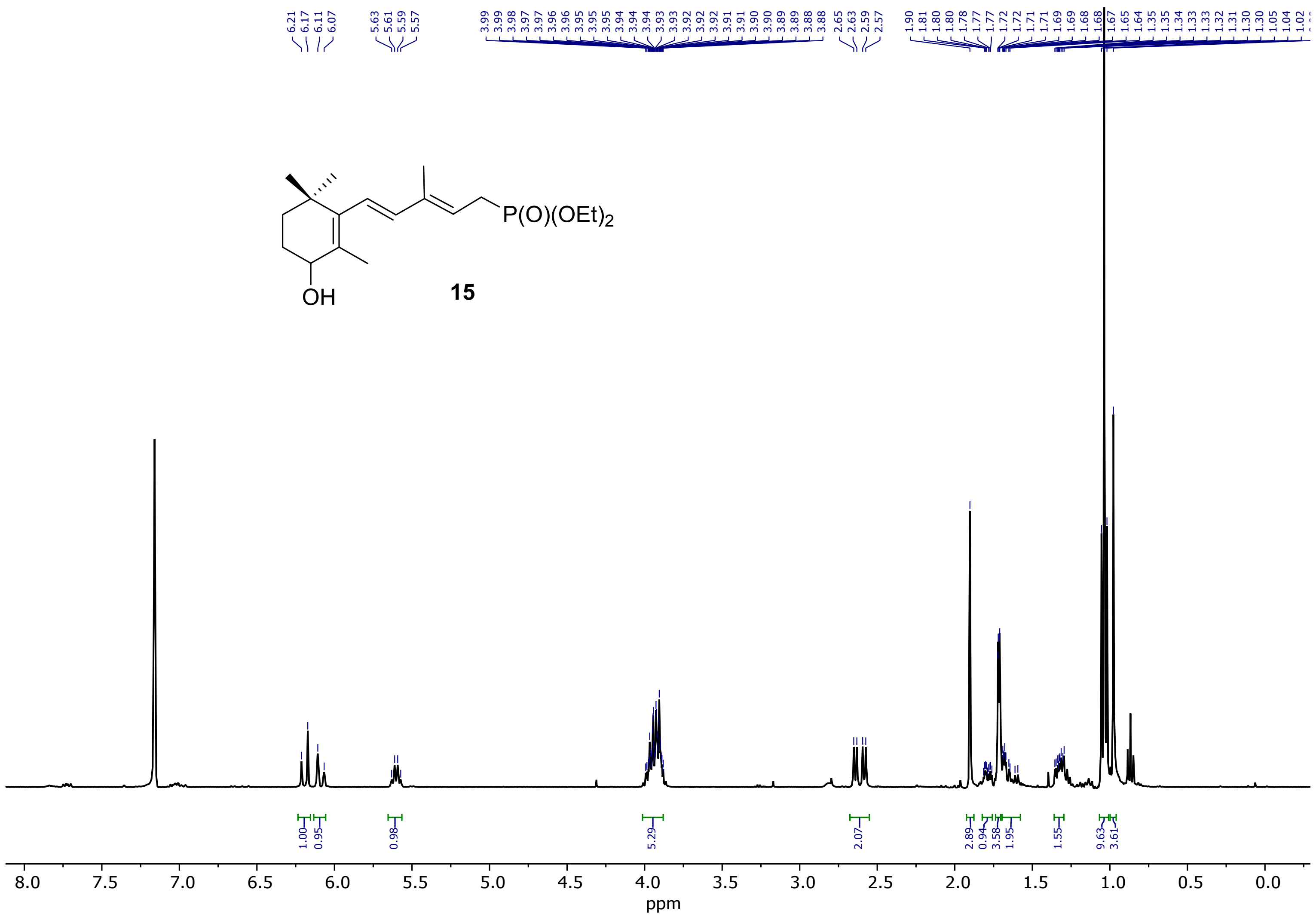

^{13}C-NMR (100.62 MHz, C_6D_6) spectrum of 15.

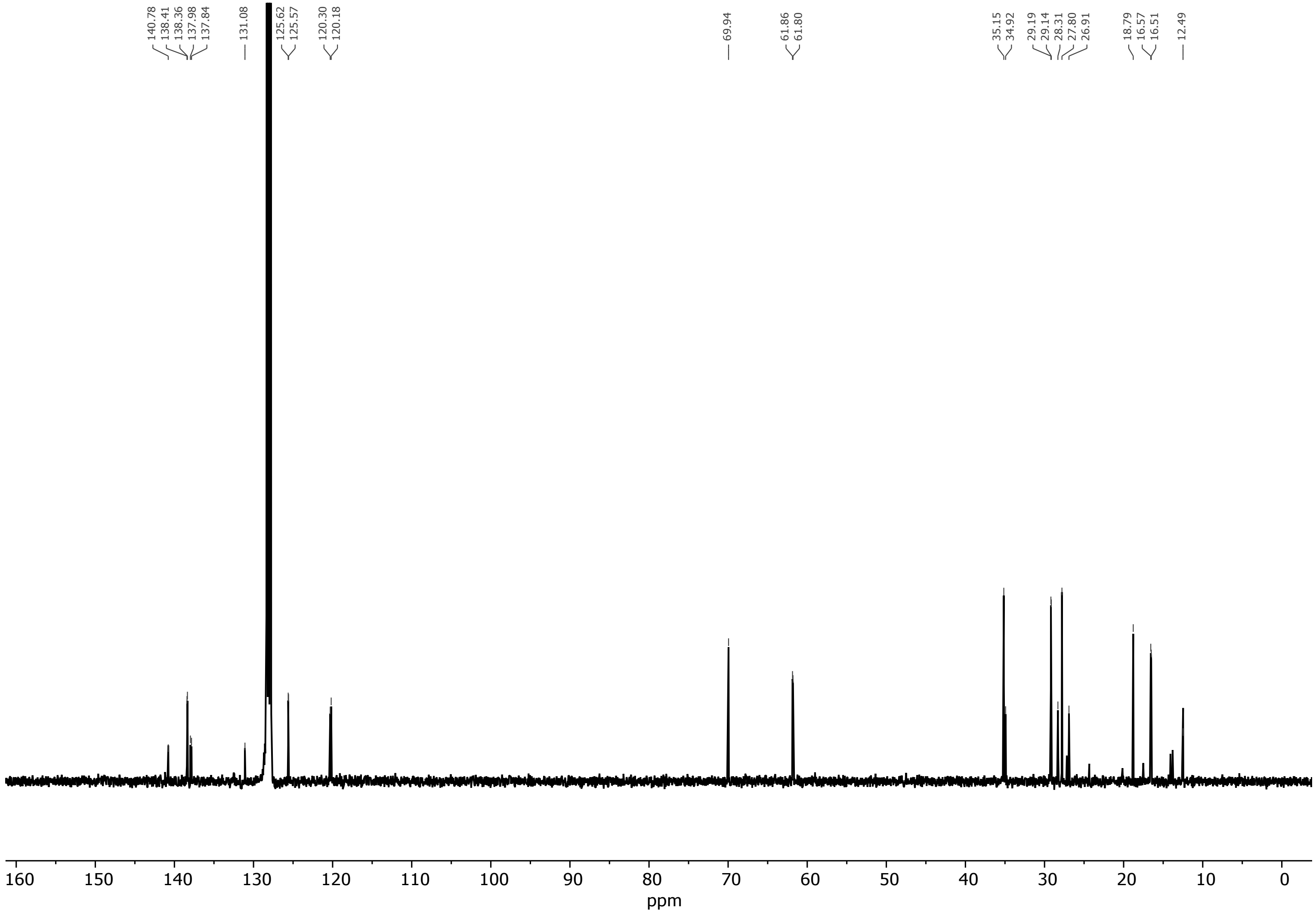

^{1}H-NMR (400.13 MHz, $CDCl_3$) spectrum of 11.

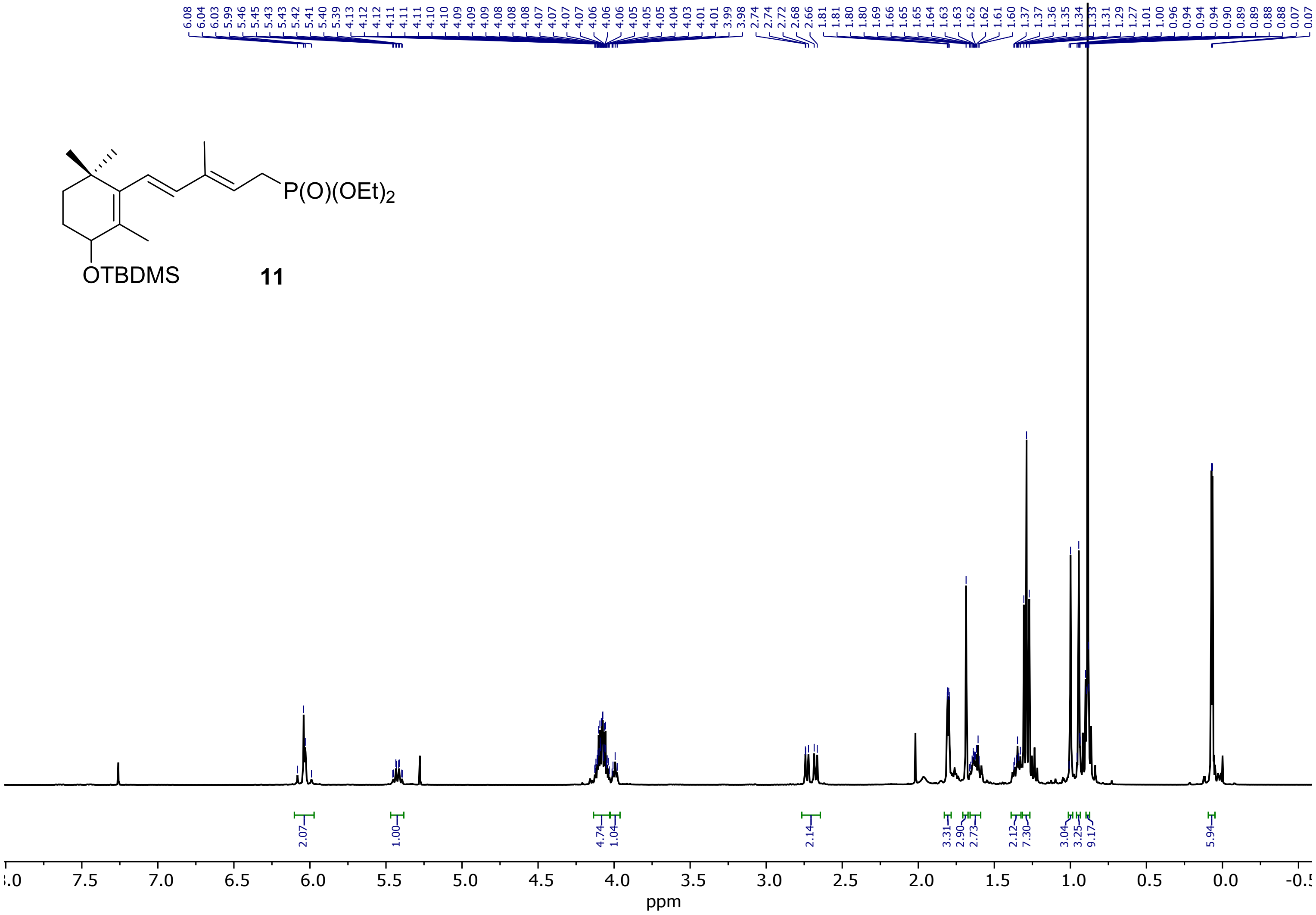

^{13}C-NMR (100.62 MHz, $CDCl_3$) spectrum of 11.

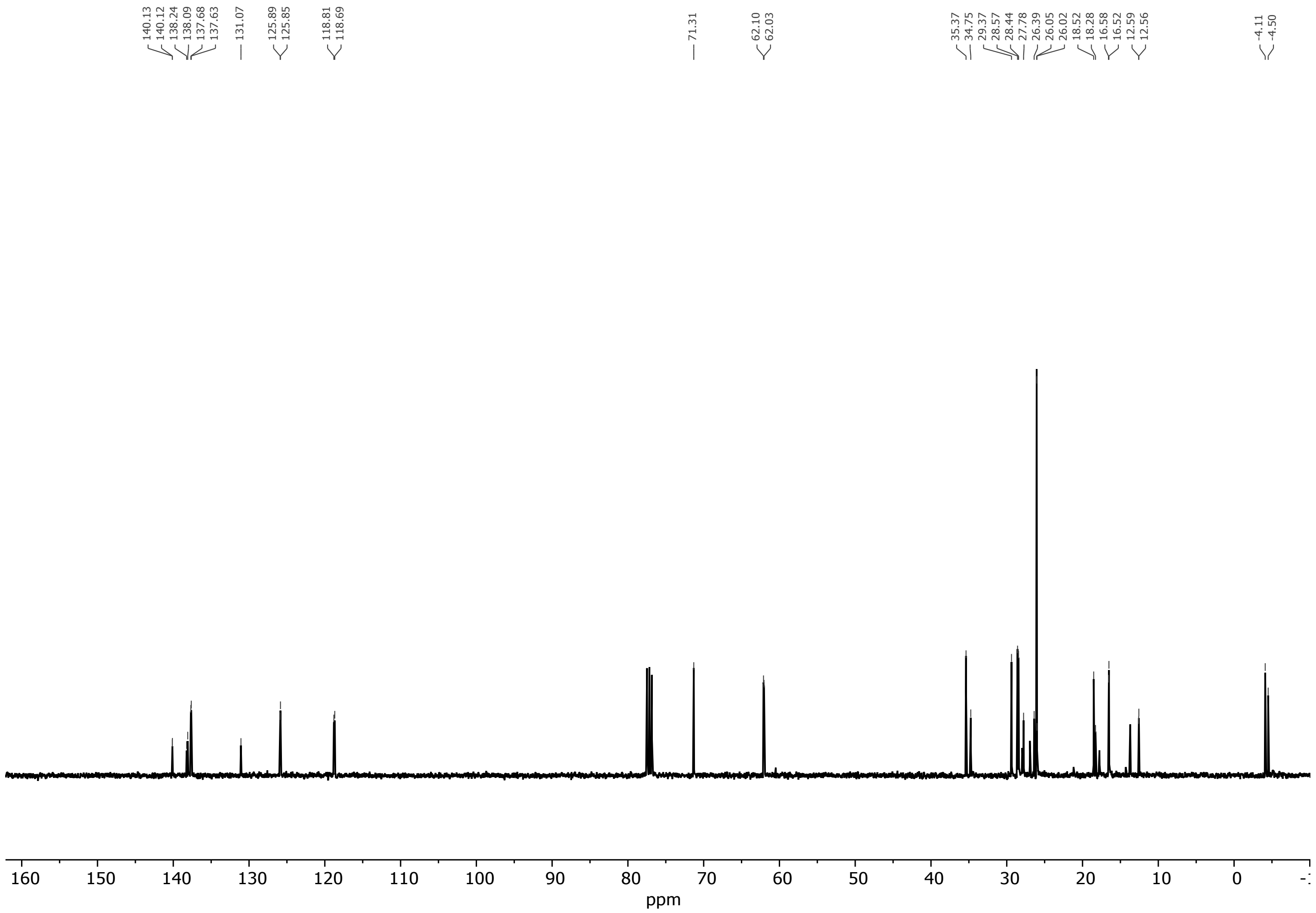

^{1}H-NMR (400.13 MHz, C_6D_6) spectrum of 17.

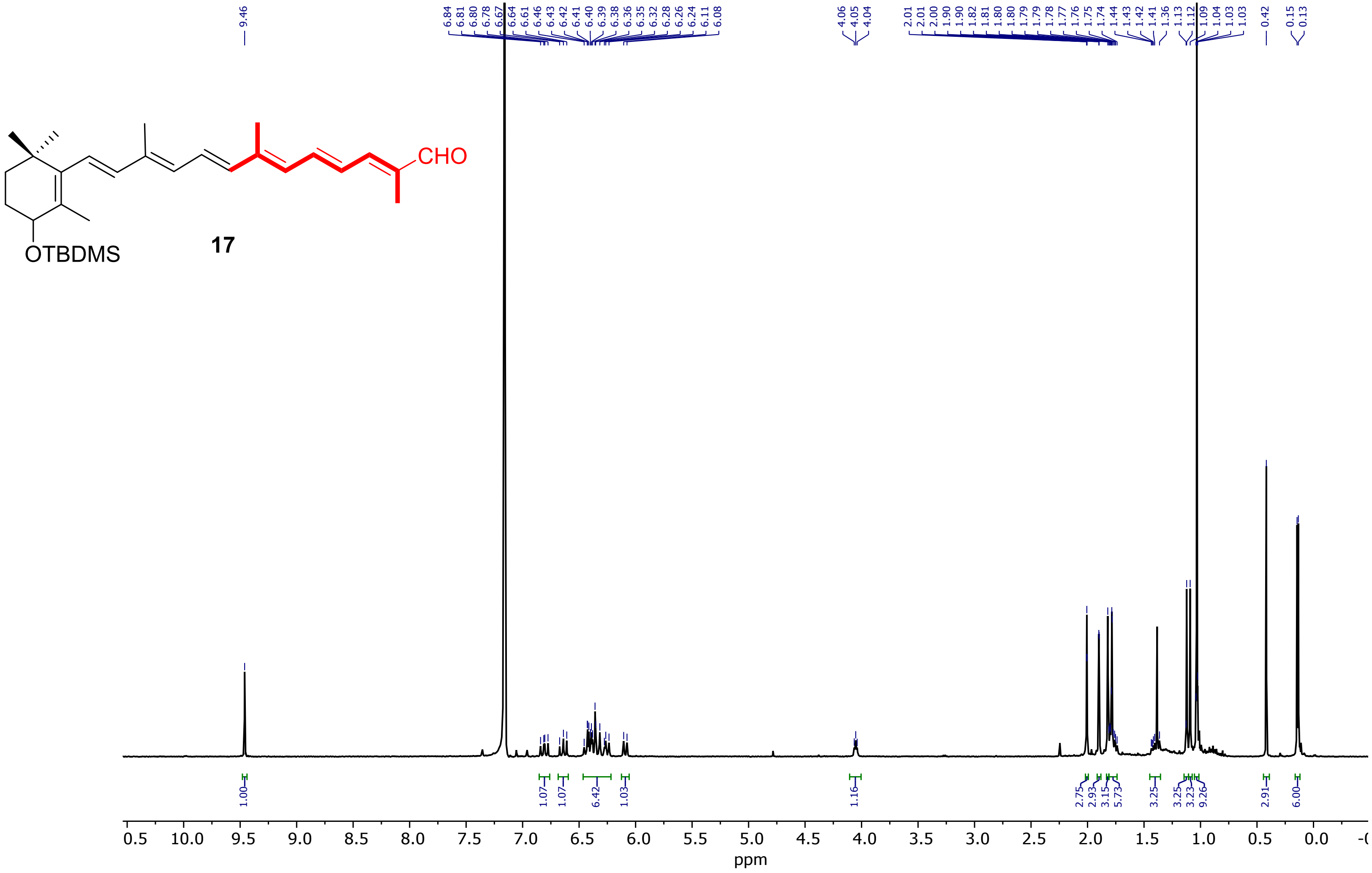

^{13}C-NMR (100.62 MHz, C_6D_6) spectrum of 17.

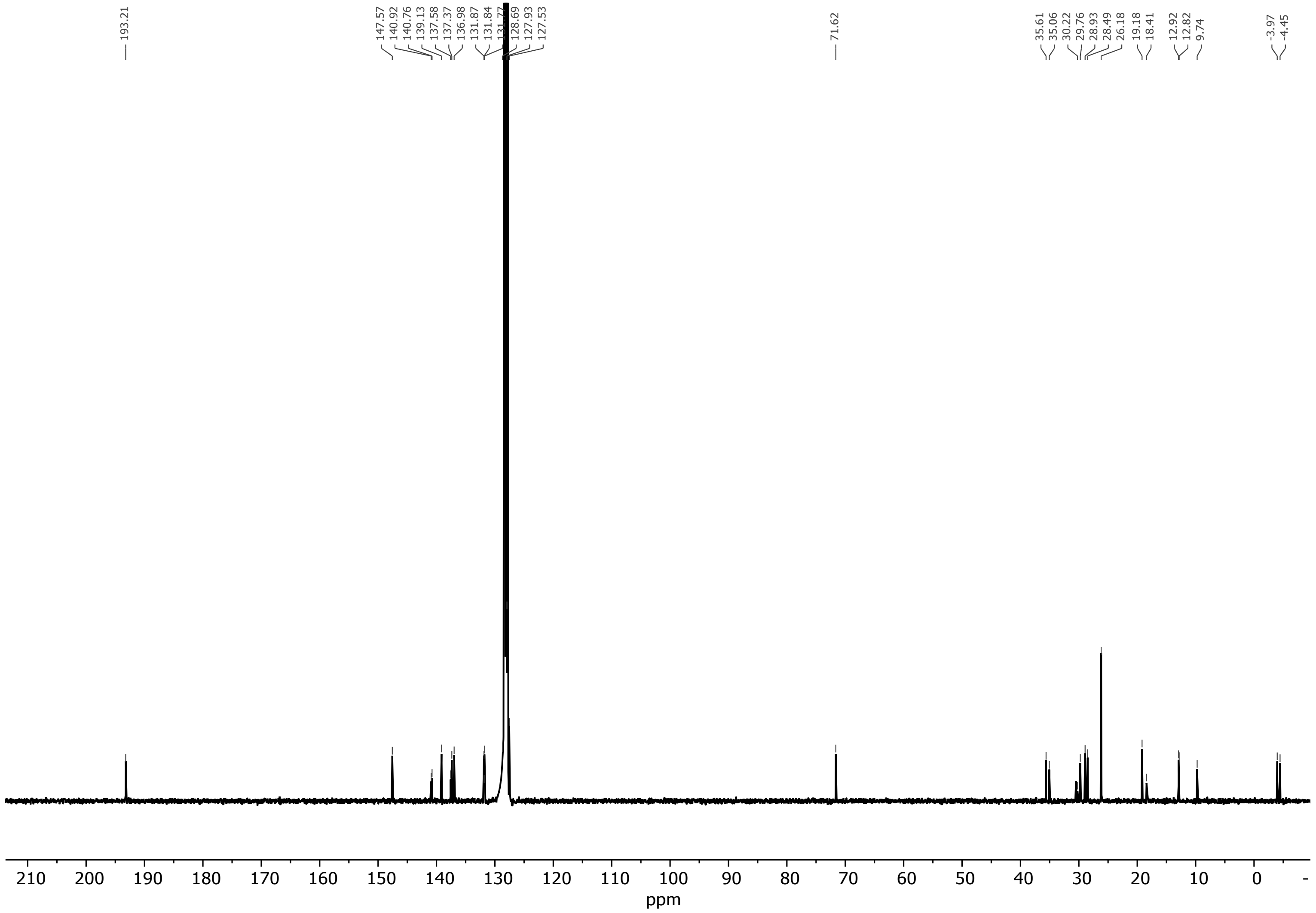

^{1}H-NMR (400.13 MHz, C_6D_6) spectrum of 19.

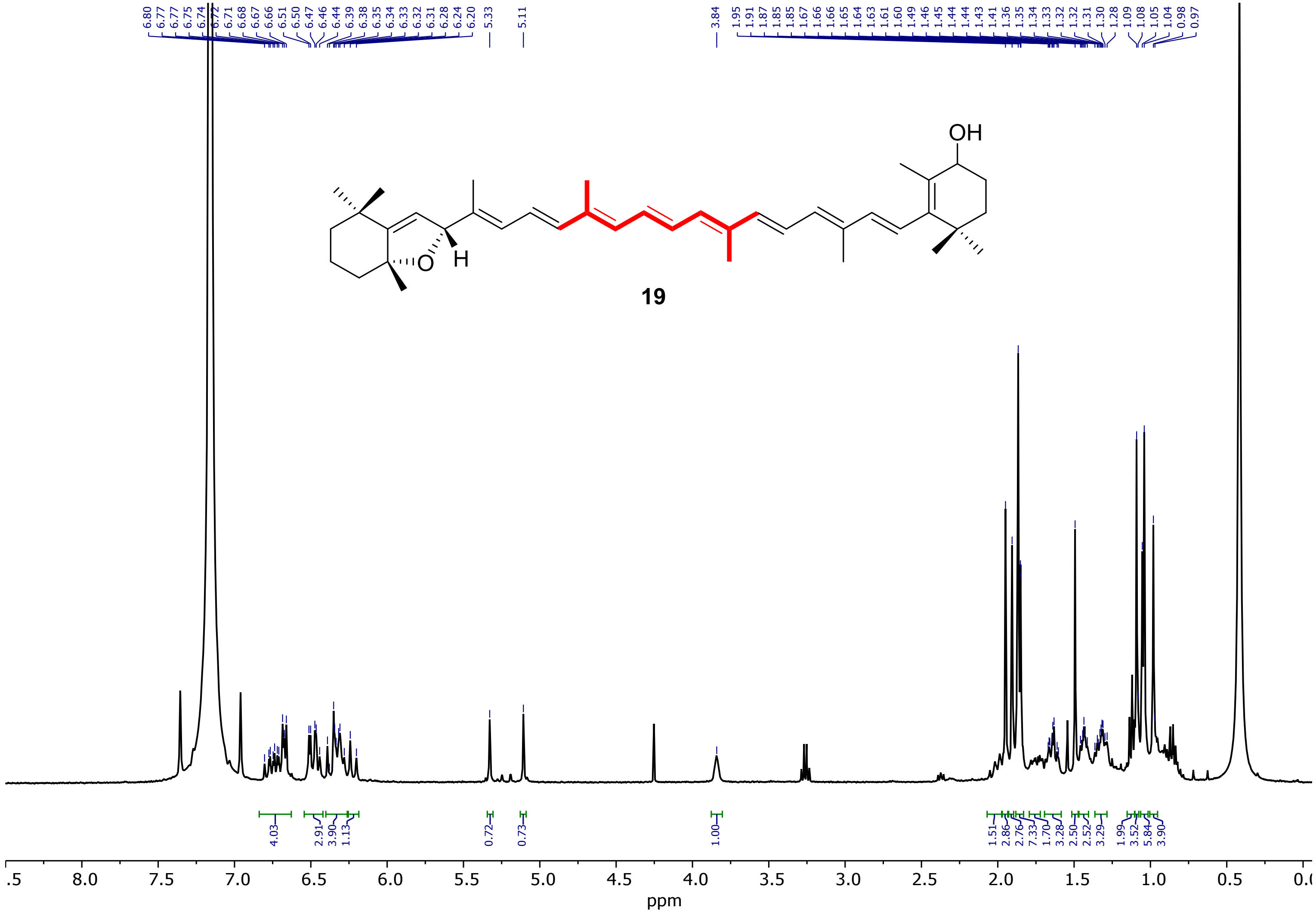

^{13}C-NMR (100.62 MHz, C_6D_6) spectrum of 19.

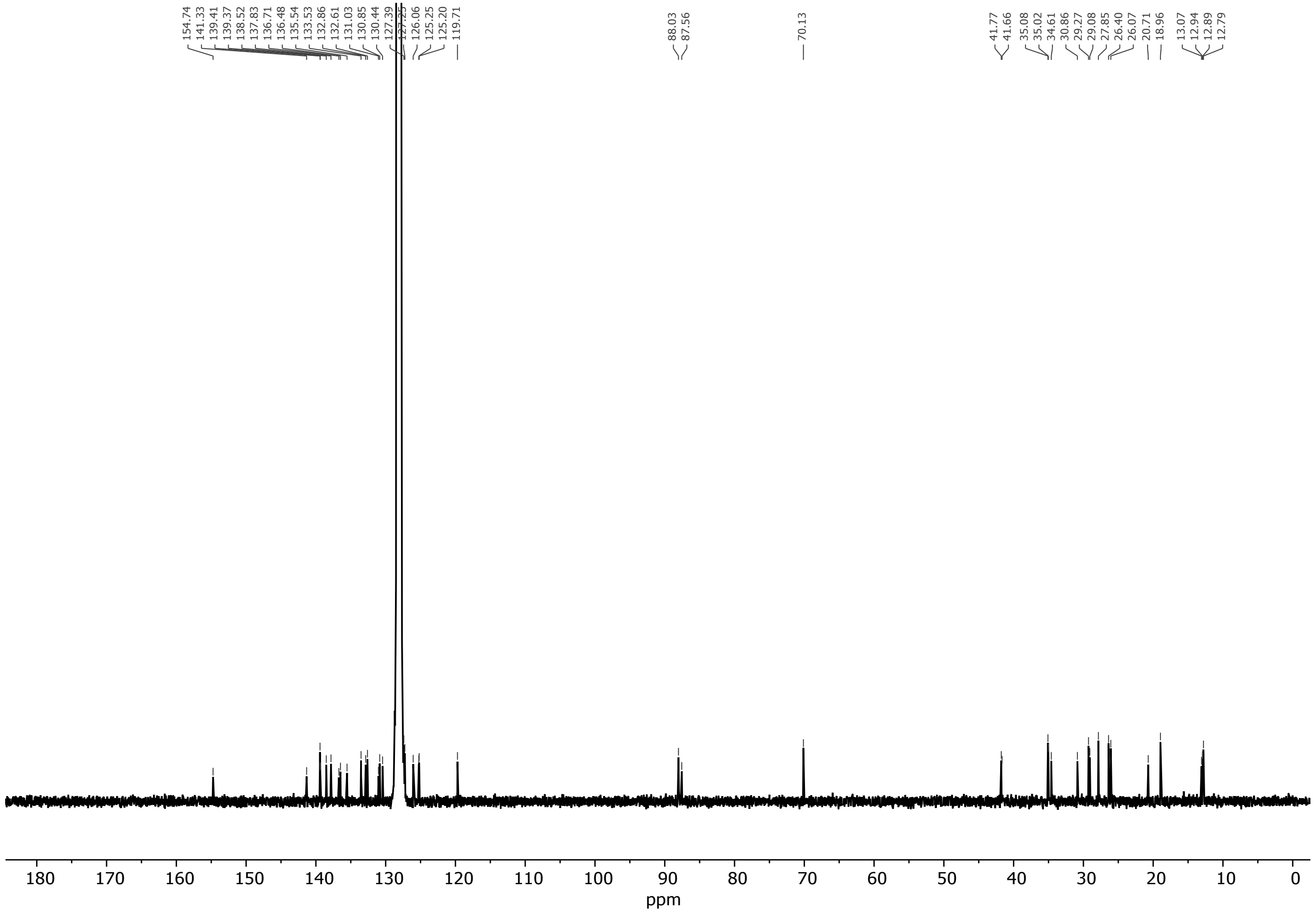

^{1}H-NMR (400.13 MHz, C_6D_6) spectrum of 2a.

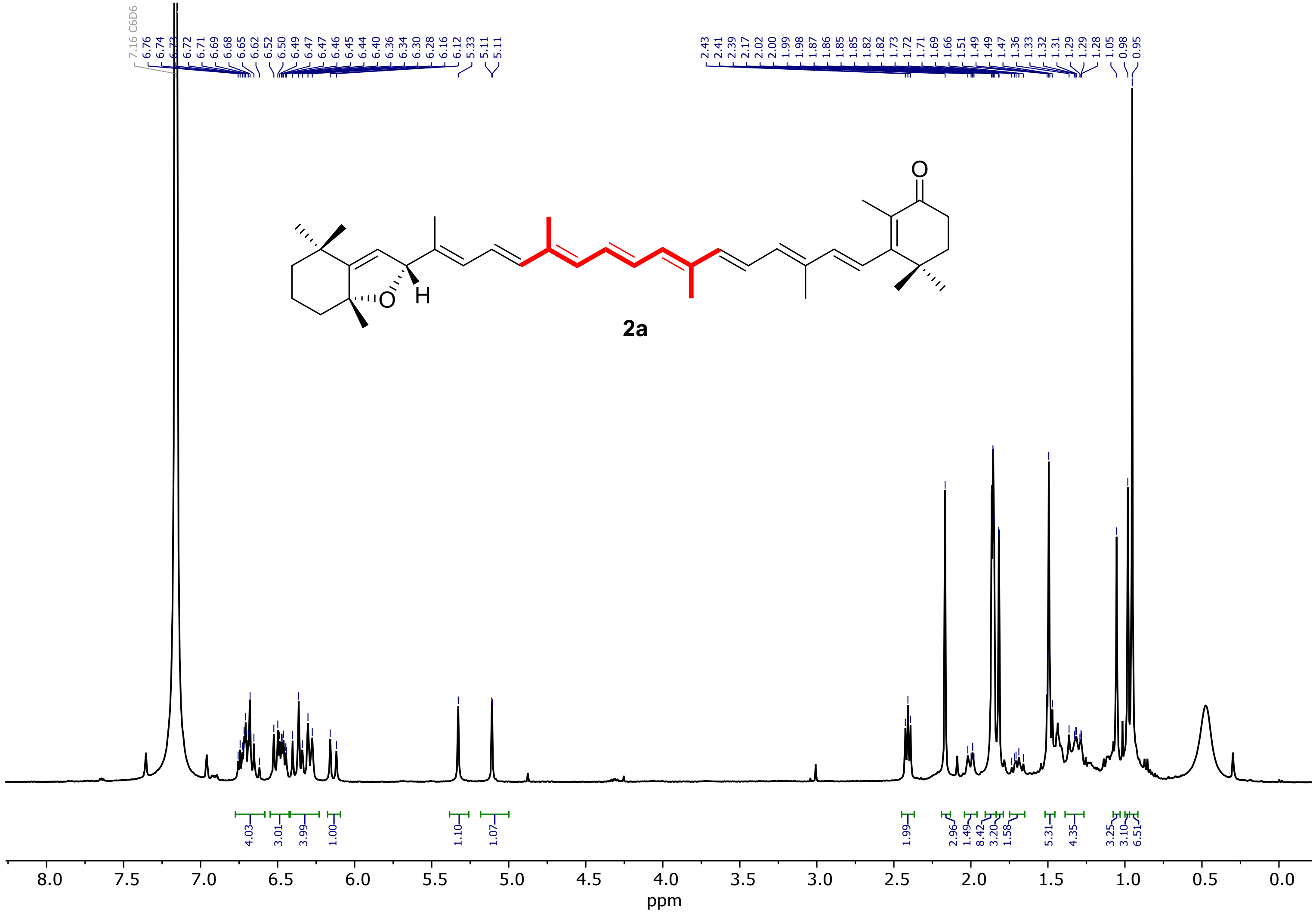

^{13}C-NMR (100.62 MHz, C_6D_6) spectrum of 2a.

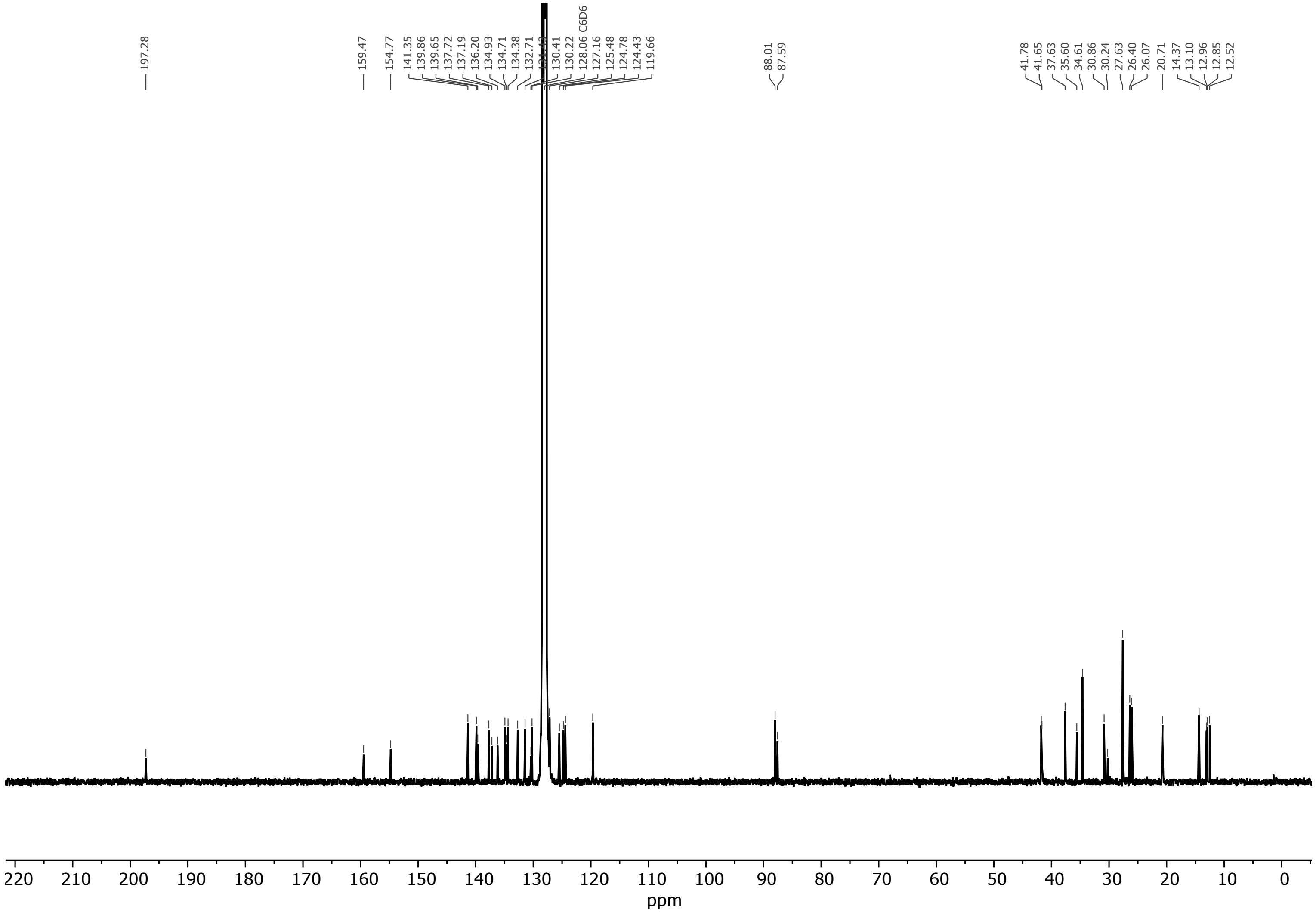

COSY (400.13 MHz, C_6D_6) spectrum of 2a.

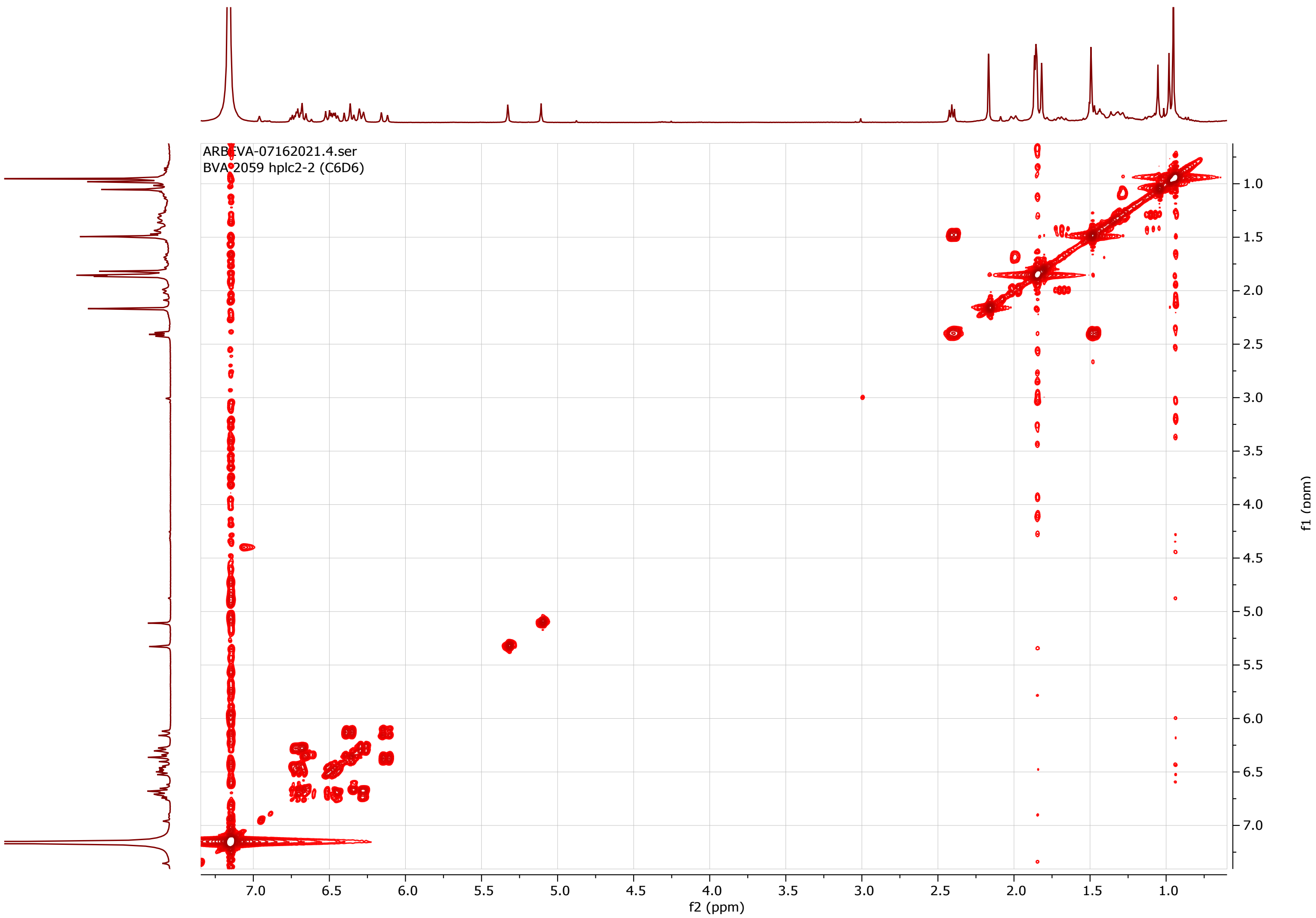

HSQC (C_6D_6) spectrum of 2a.

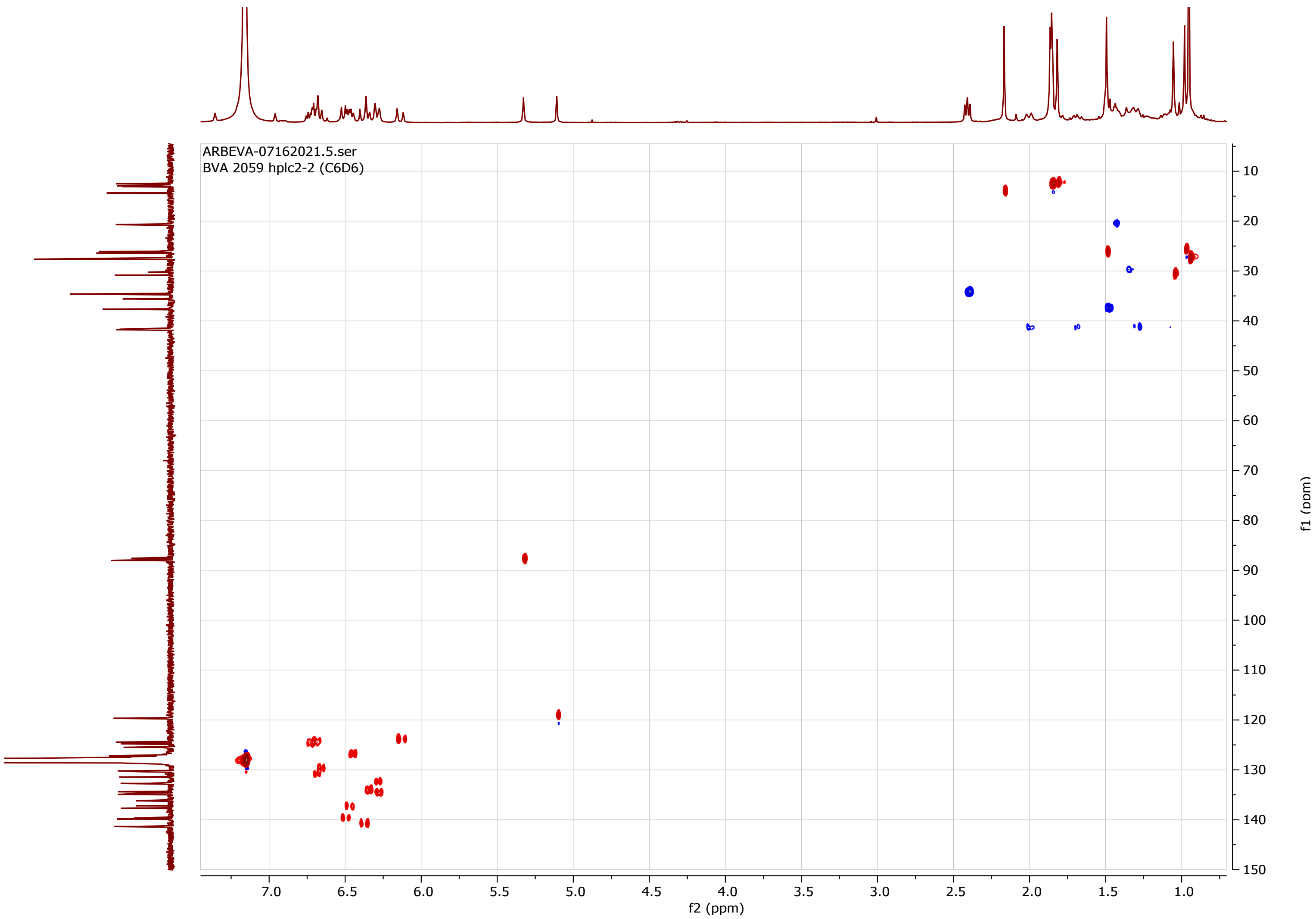

HMBC (C_6D_6) spectrum of 2a.

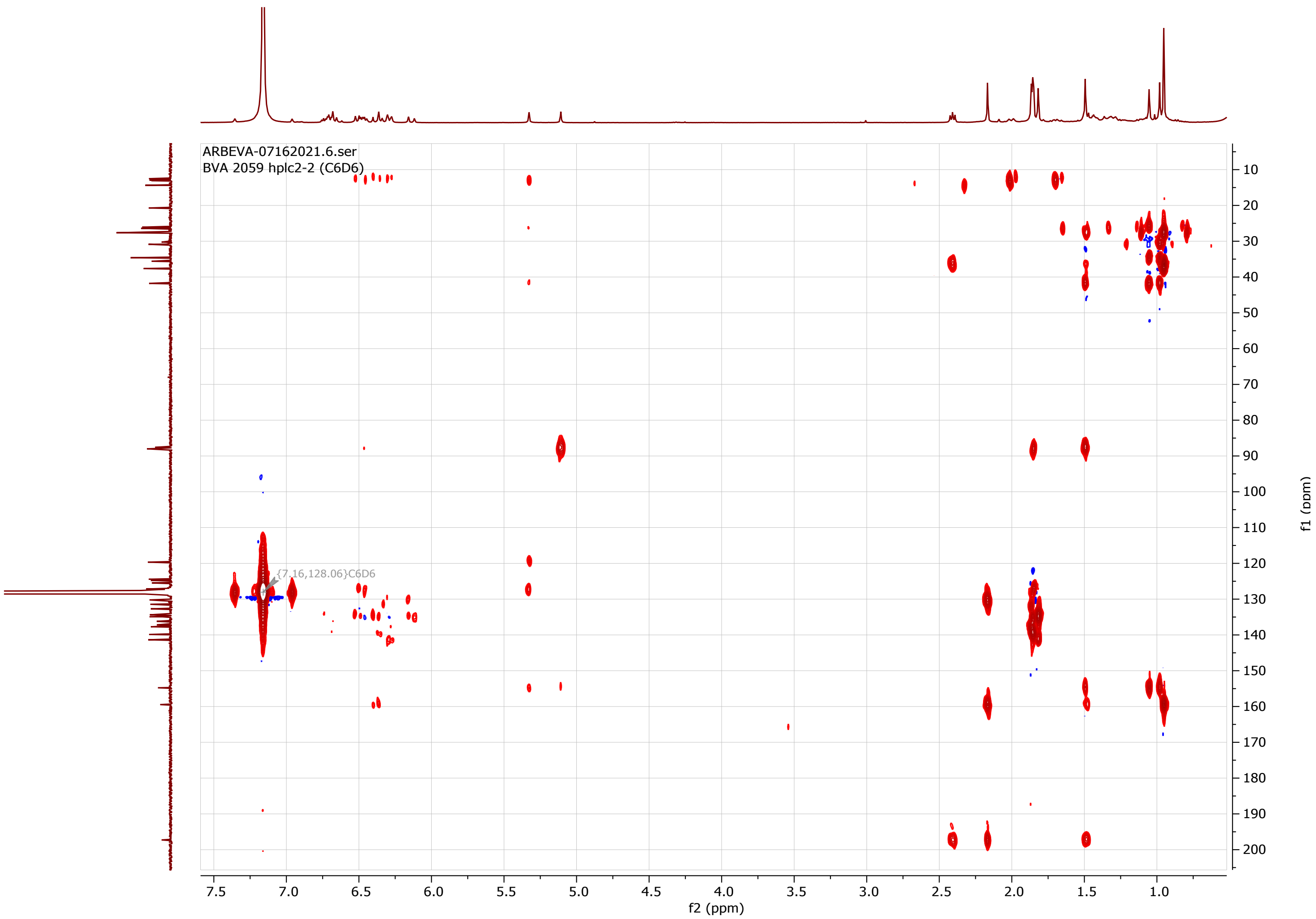

NOESY-1d (C_6D_6) spectrum of 2a.

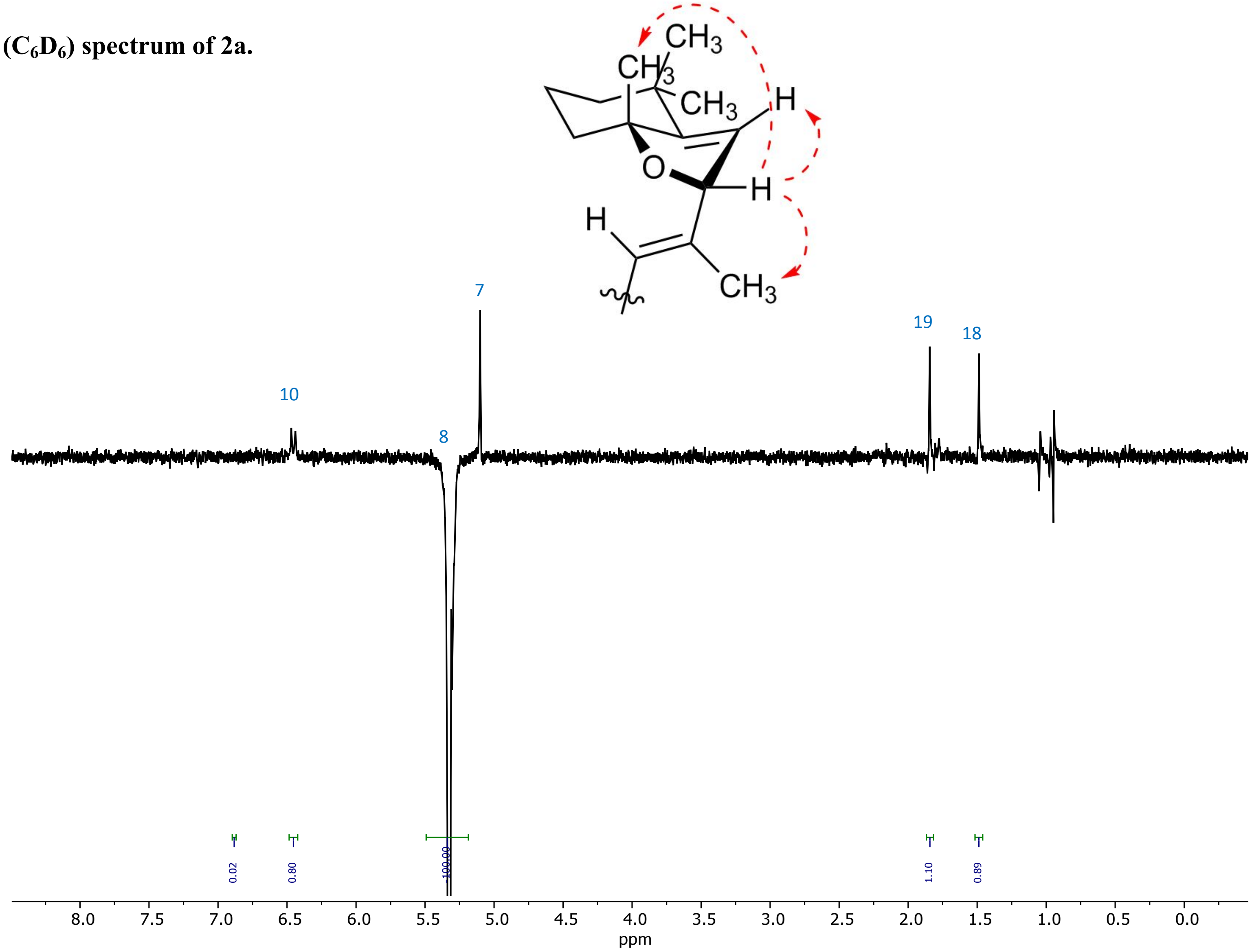

NOESY-1d (C_6D_6) spectrum of 2a.

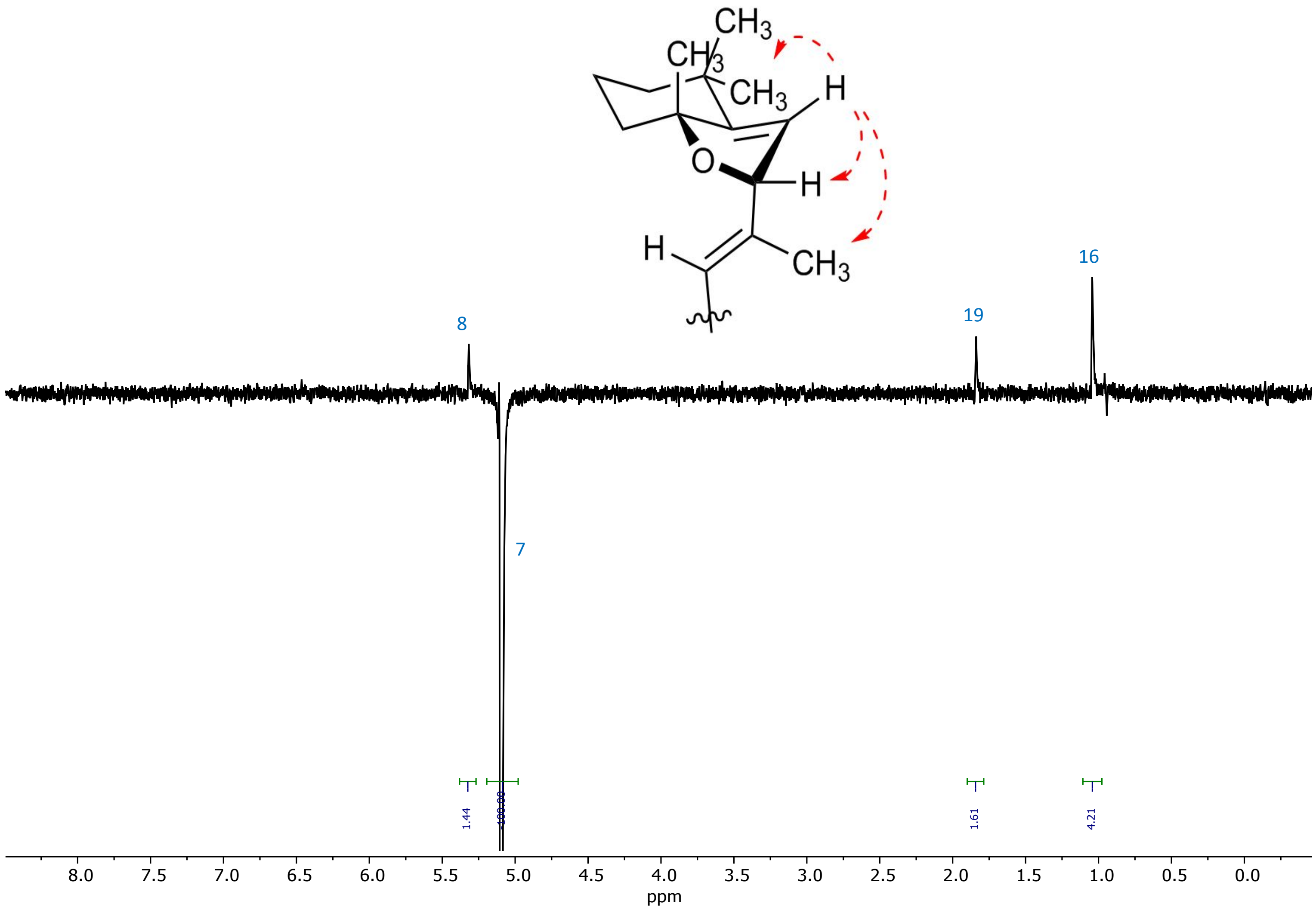

^{1}H-NMR (400.13 MHz, C_6D_6) spectrum of 2b.

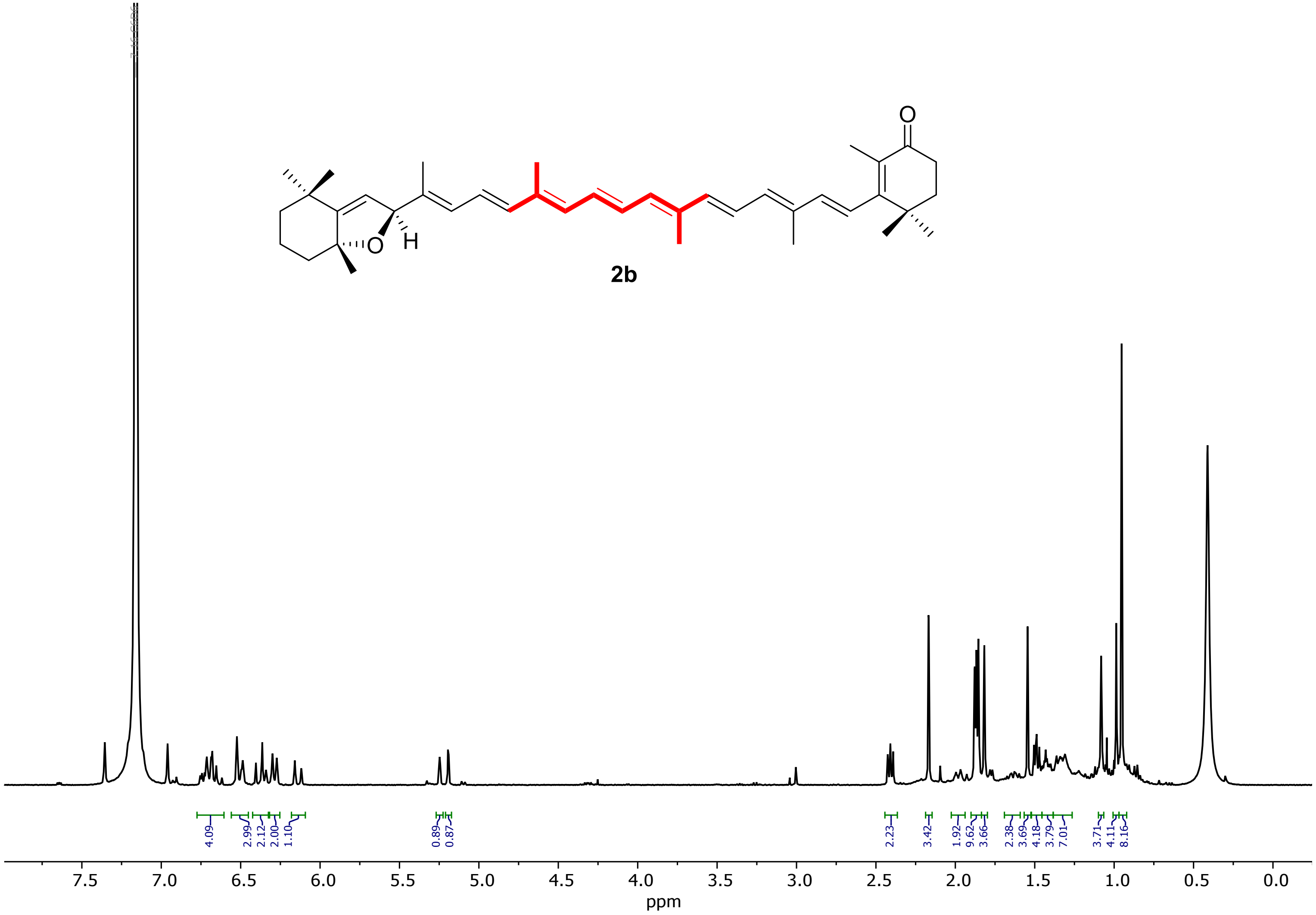

^{13}C-NMR (100.62 MHz, C_6D_6) spectrum of 2b.

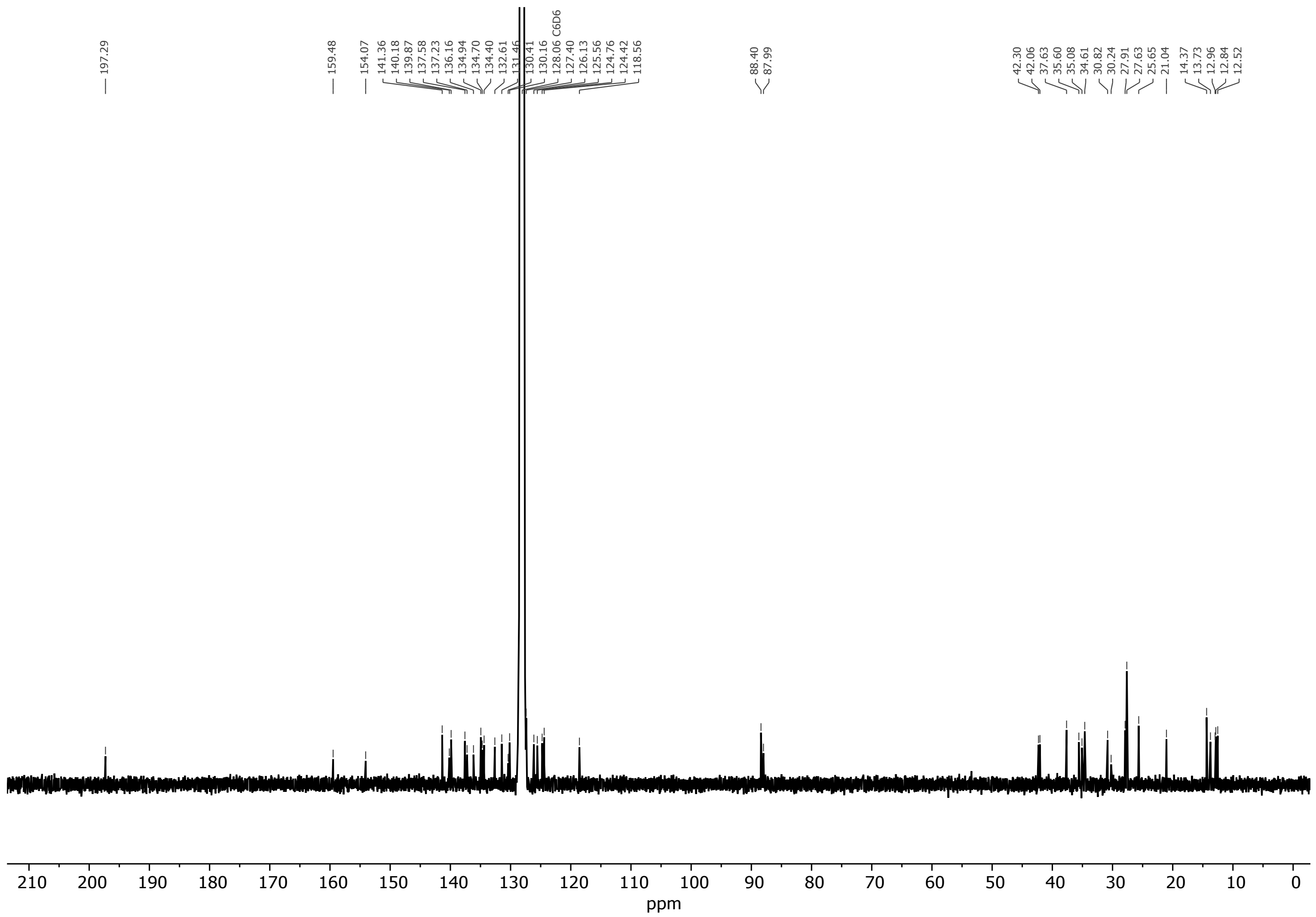

COSY (400.13 MHz, C_6D_6) spectrum of 2b.

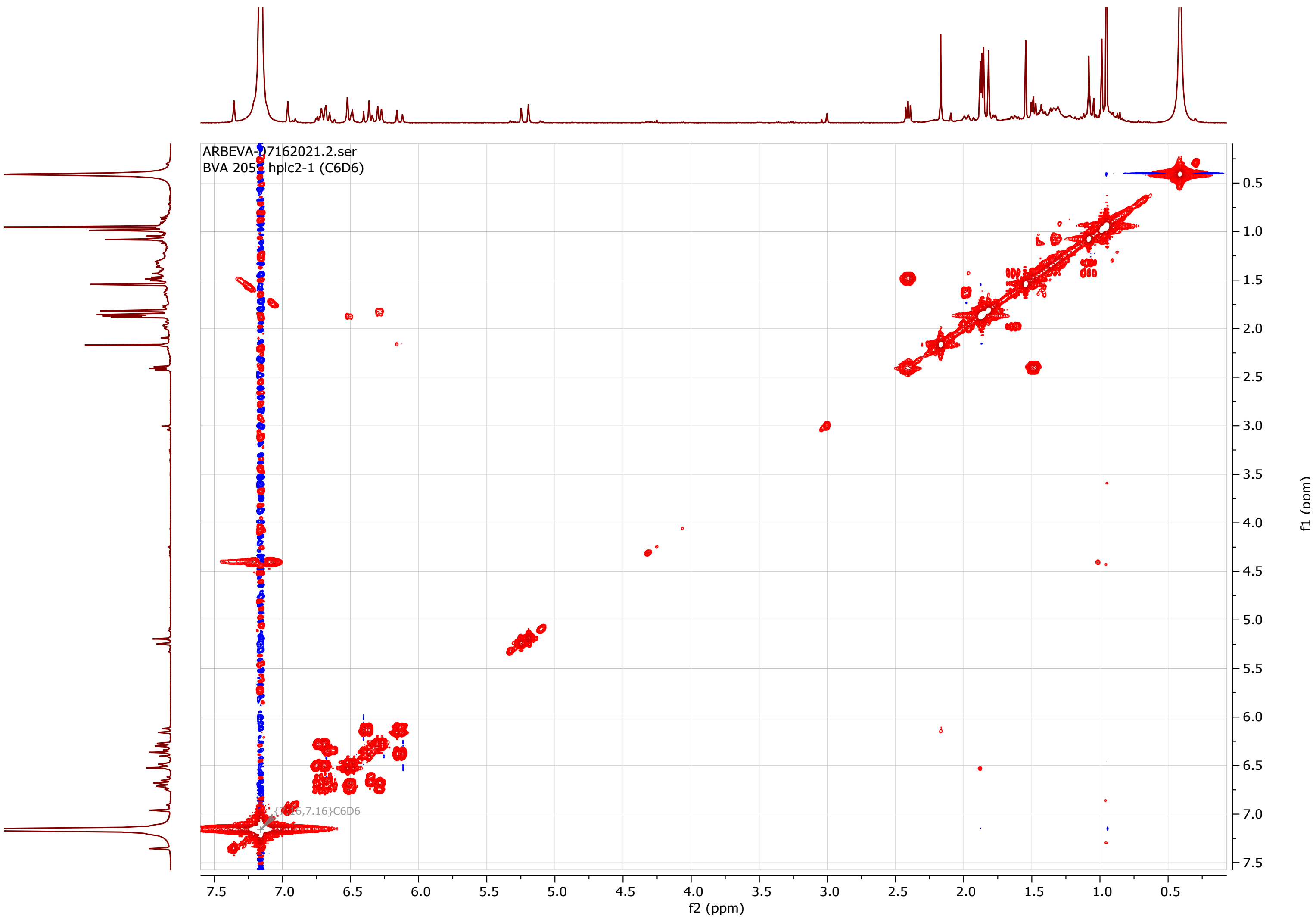

NOESY-1d (C_6D_6) spectrum of 2b

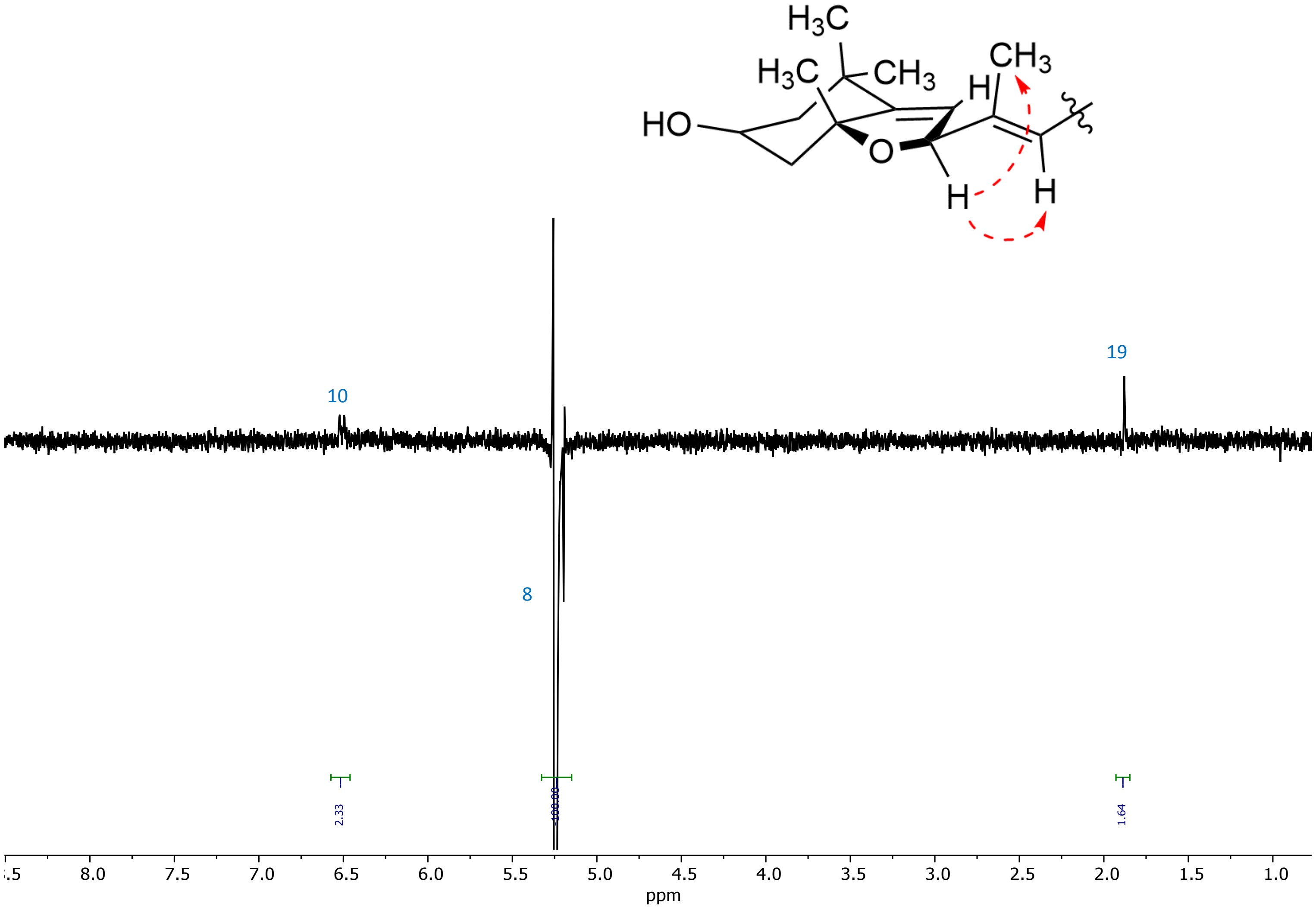

NOESY-1d (C_6D_6) spectrum of 2b

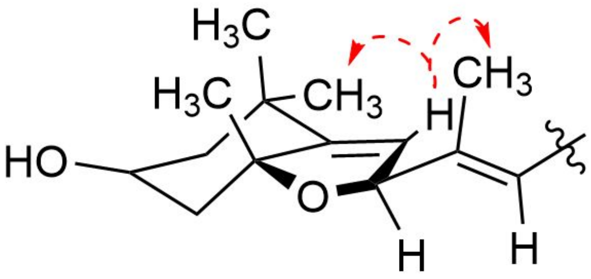

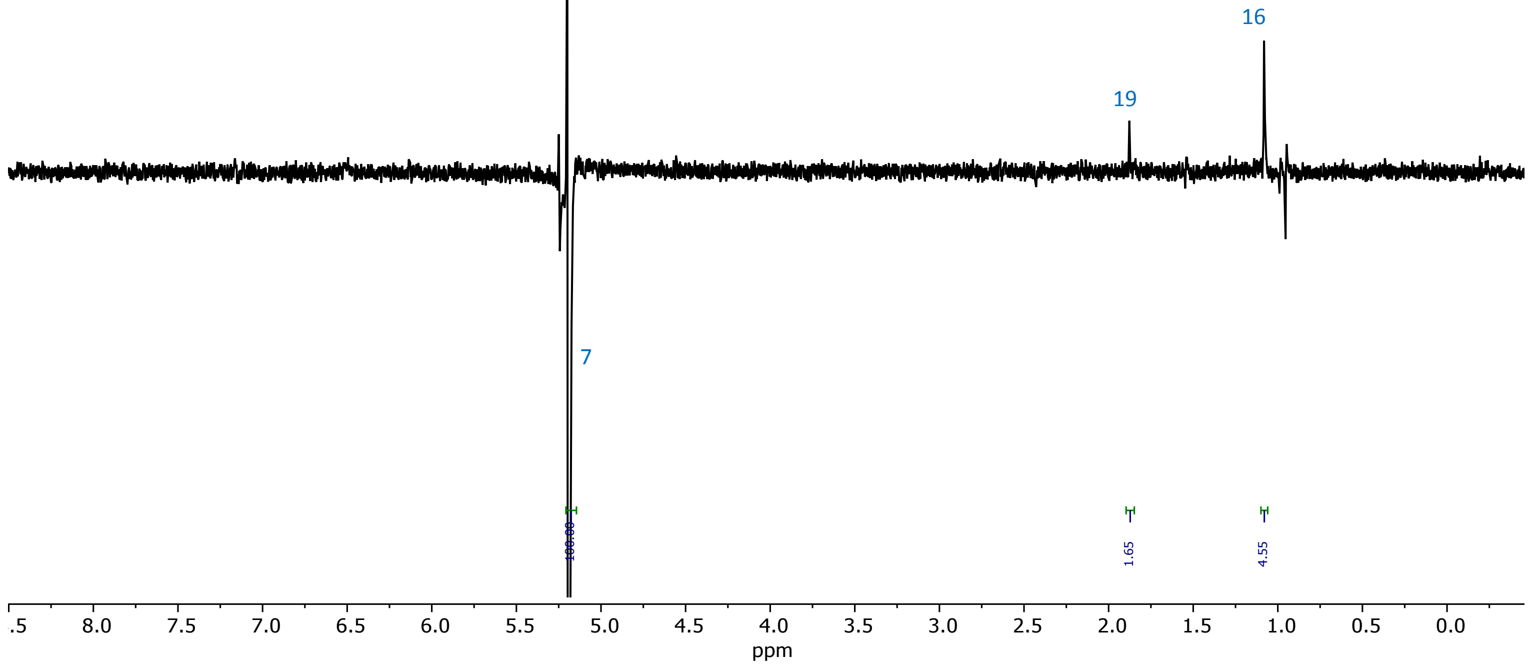

Comparison of ^{1}H-NMR spectra of 2a and 2b (400.13 MHz, $CDCl_3$).

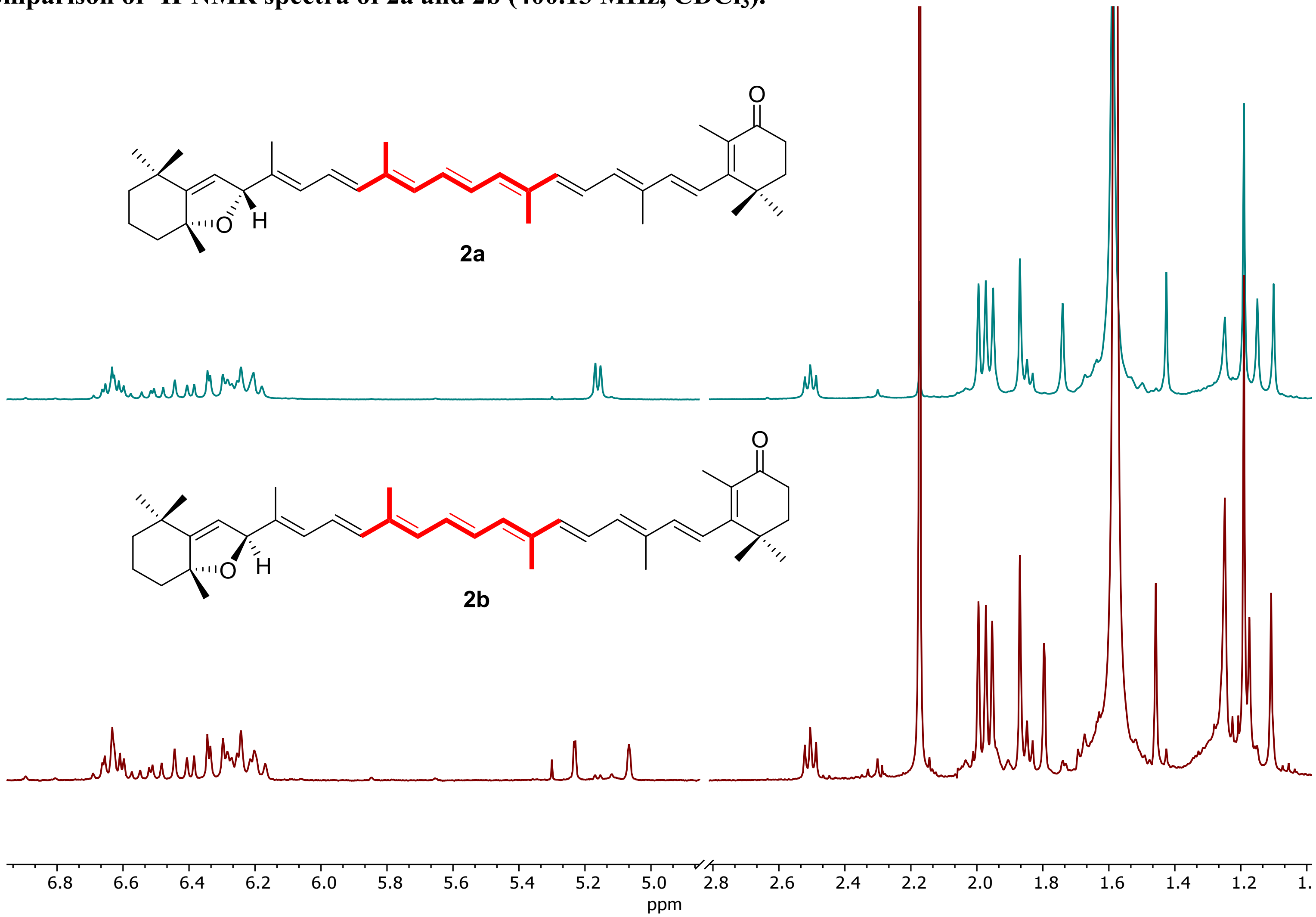

**Assignment**	**8*R* isomer 2a (Maoka *et al*)**	**8*R* isomer 2a (this work)**	**8*S* isomer 2b (Maoka et al)**	**8*S* isomer 2b (this work)**
**H11**	6.64 (dd, *J* = 15,11 Hz)	6.66 (dd, *J* = 14.4, 10.6 Hz)	6.64 (dd, *J* = 15,11 Hz)	6.66 (dd, *J* = 14.4, 11.3 Hz)
**H15; H15'**	6.63 (m)	6.63-6.57 (m)	6.63 (m)	6.63-6.57 (m)
**H11'**	6.51 (dd, *J* = 15, 11 Hz)	6.51 (dd, *J* = 15.0, 11.0 Hz)	6.51 (dd, *J* = 15, 11 Hz)	6.52 (dd, *J* = 15.0, 11.0 Hz)
**H12**	6.43 (d, *J* = 15 Hz)	6.42 (d, *J* = 14.8 Hz)	6.43 (d, *J* = 15 Hz)	6.42 (d, *J* = 14.8 Hz)
**H8**	6.37 (d, *J* = 16 Hz)	6.36 (d, *J* = 16.1 Hz)	6.37 (d, *J* = 16 Hz)	6.36 (d, *J* = 16.0 Hz)
**H14; H14'**	6.30 (d, *J* = 11 Hz)<br>6.28 (d, *J* = 11 Hz)	6.30-6.23 (m)	6.30 (d, *J* = 11 Hz)<br>6.28 (d, *J* = 11 Hz)	6.30-6.23 (m)
**H7**	6.23 (d, *J* = 16 Hz)	6.22 (d, *J* = 16.0 Hz)	6.23 (d, *J* = 16 Hz)	6.22 (d, *J* = 16.0 Hz)
**H10**	6.22 (d, *J* = 11 Hz)	6.22 (d, *J* = 10.6 Hz)	6.22 (d, *J* = 11 Hz)	6.22 (d, *J* = 11.0 Hz)
**H10'**	6.20 (d, *J* = 11 Hz)	6.19 (d, *J* = 11.0 Hz)	6.20 (d, *J* = 11 Hz)	6.18 (d, *J* = 11.0 Hz)
**H7'**	5.17 (br s)	5.17 (br s)	5.23 (d, *J* = 1.5 Hz)	5.23 (d, *J* = 1.7 Hz)
**H8'**	5.16 (br s)	5.15 (br s)	5.07 (br s)	5.07 (br s)
**H3 (2H)**	2.51 (t, *J* = 7.5 Hz)	2.50 (t, *J* = 6.8 Hz)	2.51 (t, *J* = 7.5 Hz)	2.50 (t, *J* = 6.8 Hz)
**H20 (3H)**	2.00 (s)	2.00 (s)	2.00 (s)	2.00 (s)
**H20' (3H)**	1.98 (s)	1.97 (s)	1.98 (s)	1.97 (s)
**H18 (3H)**	1.96 (s)	1.95 (s)	1.96 (s)	1.95 (s)
**H19 (3H)**	1.87 (s)	1.87 (s)	1.87 (s)	1.87 (s)
**H2 (2H)**	1.85 (t, *J* = 7.5 Hz)	1.85 (t, *J* = 6.8 Hz)	1.85 (t, *J* = 7.5 Hz)	1.85 (t, *J* = 6.8 Hz)
**H19' (3H)**	1.75 (s)	1.74 (s)	1.80 (s)	1.80 (s)
**H18' (3H)**	1.43 (s)	1.43 (s)	1.46 (s)	1.46 (s)
**H16; H17 (6H)**	1.20 (s)	1.19 (s)	1.20 (s)	1.19 (s)
**H16' (3H)**	1.15 (s),	1.15 (s)	1.18 (s)	1.17 (s)
**H17' (3H)**	1.10 (s)	1.10 (s)	1.11 (s)	1.11 (s)

Ref: T. Maoka, M. Otani, M. Z. Khan, M. Takemura, J. Hattan, N. Misawa. *Tetrahedron Letters* **2016**, *57*, 4746–4748
